# Supplementary material for: New Azaphilones from the Marine-Derived Fungus Penicillium sclerotiorum E23Y-1A with Their Anti-Inflammatory and Antitumor Activities
Source: Mar Drugs. 2023 Jan 22;21(2):75. doi: 10.3390/md21020075 (PMC9961037; doi:10.3390/md21020075)

Supporting Information

New Azaphilones from the Marine-Derived Fungus *Penicillium sclerotiorum* E23Y-1A with Their Anti-Inflammatory and Antitumor Activities

Yanbo Zeng [1,2,3,†,*], Zhi Wang [1,2,†], Wenjun Chang [1,3], Weibo Zhao [1,2], Hao Wang [1], Huiqin Chen [1], Haofu Dai [1,*], and Fang Lv [2,*]

[1] Hainan Provincial Key Laboratory for Functional Components Research and Utilization of Marine Bio-resources , Institute of Tropical Bioscience and Biotechnology, Chinese Academy of Tropical Agricultural Sciences & Key Laboratory for Biology and Genetic Resources of Tropical Crops of Hainan Province, Hainan Institute for Tropical Agricultural Resources, Haikou 571101, China; 13269326329@163.com (Z.W.); changwenjun@itbb.org.cn (W.C.); zwb1175642690@163.com (W.Z.); wanghao@itbb.org.cn (H.W.); chenhuiqin@itbb. org.cn (H.C.)

[2] Beijing Key Laboratory for Separation and Analysis in Biomedicine and Pharmaceuticals, School of Life Science, Beijing Institute of Technology, Beijing, 100081, China;

[3] Zhanjiang Experimental Station of Chinese Academy of Tropical Agricultural Sciences, Zhanjiang, 524013, China

*Corresponding authors: zengyanbo@itbb.org.cn (Y.Z.); daihaofu@itbb.org.cn (H.D.); lvfangbei jing@bit.edu.cn (F.L.)

[†] These authors contributed equally to this work.

Figure S1. The results of the DP4+ analysis of **1**

Table S1. Experimental and calculated ^{13}C NMR chemical shifts of **1**

Table S2. Experimental and calculated ^{1}H NMR chemical shifts of **1**

Figure S2. Key MOs in important transitions of most populated conformer of **1**

Figure S3. Linear regression analysis between experimental and calculated ^{13}C and ^{1}H NMR chemical shifts of isomers of **2**

Figure S4. The results of the DP4+ analysis of **2**

Table S3. Experimental and calculated ^{13}C NMR chemical shifts of **2**

Table S4. Experimental and calculated ^{1}H NMR chemical shifts of **2**

Figure S5. Linear regression analysis between experimental and calculated ^{13}C and ^{1}H NMR chemical shifts of isomers of **3**

Figure S6. The results of the DP4+ analysis of **3**

Table S5. Experimental and calculated ^{13}C NMR chemical shifts of **3**

Table S6. Experimental and calculated ^{1}H NMR chemical shifts of **3**

Figure S7. Linear regression analysis between experimental and calculated ^{13}C and ^{1}H NMR chemical shifts of isomers of **4**

Figure S8. The results of the DP4+ analysis of **4**

Table S7. Experimental and calculated ^{13}C NMR chemical shifts of **4**

Table S8. Experimental and calculated ^{1}H NMR chemical shifts of **4**

Figure S9. ^{1}H-^{1}H COSY and key HMBC correlations of compound **2**

Figure S10. The key NOESY correlations of compound **2**

Figure S11. ^{1}H-^{1}H COSY and key HMBC correlations of compound **3**

Figure S12. The key NOESY correlations of compound **3**

Figure S13. ^{1}H-^{1}H COSY and key HMBC correlations of compound **5**

Figure S14. The key NOESY correlations of compound **5**

Figure S15. ^{1}H-^{1}H COSY and key HMBC correlations of compound **7**

Figure S16. The key NOESY correlations of compound **7**

Figure S17. ^{1}H-^{1}H COSY and key HMBC correlations of compound **8**

Figure S18. The key NOESY correlations of compound **8**

Figure S19. ^{1}H NMR (600 MHz, $CDCl_3$) spectrum of compound **1**

Figure S20. ^{13}C NMR (125 MHz, $CDCl_3$) spectrum of compound **1**

Figure S21. DEPT spectrum of compound **1**

Figure S22. HSQC spectrum of compound **1**

Figure S23. HMBC spectrum of compound **1**

Figure S24. ^1H-^1H COSY spectrum of compound **1**

Figure S25. NOESY spectrum of compound **1**

Figure S26. HRESIMS spectrum of compound **1**

Figure S27. UV spectrum of compound **1**

Figure S28. IR spectrum of compound **1**

Figure S29. ^1H NMR (600 MHz, $CDCl_3$) spectrum of compound **2**

Figure S30. ^{13}C NMR (125 MHz, $CDCl_3$) spectrum of compound **2**

Figure S31. DEPT spectrum of compound **2**

Figure S32. HSQC spectrum of compound **2**

Figure S33. HMBC spectrum of compound **2**

Figure S34. ^1H-^1H COSY spectrum of compound **1**

Figure S35. NOESY spectrum of compound **2**

Figure S36. HRESIMS spectrum of compound **2**

Figure S37. UV spectrum of compound **2**

Figure S38. IR spectrum of compound **2**

Figure S39. ^1H NMR (400 MHz, $CDCl_3$) spectrum of compound **3**

Figure S40. ^{13}C NMR (125 MHz, $CDCl_3$) spectrum of compound **3**

Figure S41. DEPT spectrum of compound **3**

Figure S42. HSQC spectrum of compound **3**

Figure S43. HMBC spectrum of compound **3**

Figure S44. ^1H-^1H COSY spectrum of compound **3**

Figure S45. ROESY spectrum of compound **3**

Figure S46. HRESIMS spectrum of compound **3**

Figure S47. UV spectrum of compound **3**

Figure S48. IR spectrum of compound **3**

Figure S49. ^1H NMR (600 MHz, $CDCl_3$) spectrum of compound **4**

Figure S50. ^{13}C NMR (125 MHz, $CDCl_3$) spectrum of compound **4**

Figure S51. DEPT spectrum of compound **4**

Figure S52. HSQC spectrum of compound **4**

Figure S53. HMBC spectrum of compound **4**

Figure S54. ^{1}H-^{1}H COSY spectrum of compound **4**

Figure S55. NOESY spectrum of compound **4**

Figure S56. HRESIMS spectrum of compound **4**

Figure S57. UV spectrum of compound **4**

Figure S58. IR spectrum of compound **4**

Figure S59. ^{1}H NMR (600 MHz, $CDCl_3$) spectrum of compound **5**

Figure S60. ^{13}C NMR (125 MHz, $CDCl_3$) spectrum of compound **5**

Figure S61. DEPT spectrum of compound **5**

Figure S62. HSQC spectrum of compound **5**

Figure S63. HMBC spectrum of compound **5**

Figure S64. ^{1}H-^{1}H COSY spectrum of compound **5**

Figure S65. NOESY spectrum of compound **5**

Figure S66. HRESIMS spectrum of compound **5**

Figure S67. UV spectrum of compound **5**

Figure S68. IR spectrum of compound **5**

Figure S69. ^{1}H NMR (600 MHz, $CDCl_3$) spectrum of compound **6**

Figure S70. ^{13}C NMR (125 MHz, $CDCl_3$) spectrum of compound **6**

Figure S71. DEPT spectrum of compound **6**

Figure S72. HSQC spectrum of compound **6**

Figure S73. HMBC spectrum of compound **6**

Figure S74. ^{1}H-^{1}H COSY spectrum of compound **6**

Figure S75. NOESY spectrum of compound **6**

Figure S76. HRESIMS spectrum of compound **6**

Figure S77. UV spectrum of compound **6**

Figure S78. IR spectrum of compound **6**

Figure S79. ^{1}H NMR (600 MHz, $CDCl_3$) spectrum of compound **7**

Figure S80. ^{13}C NMR (125 MHz, $CDCl_3$) spectrum of compound **7**

Figure S81. DEPT spectrum of compound **7**

Figure S82. HSQC spectrum of compound **7**

Figure S83. HMBC spectrum of compound **7**

Figure S84. ^{1}H-^{1}H COSY spectrum of compound **7**

Figure S85. NOESY spectrum of compound **7**

Figure S86. HRESIMS spectrum of compound **7**

Figure S87. UV spectrum of compound **7**

Figure S88. IR spectrum of compound **7**

Figure S89. ^{1}H NMR (600 MHz, $CDCl_3$) spectrum of compound **8**

Figure S90. ^{13}C NMR (125 MHz, $CDCl_3$) spectrum of compound **8**

Figure S91. DEPT spectrum of compound **8**

Figure S92. HSQC spectrum of compound **8**

Figure S93. HMBC spectrum of compound **8**

Figure S94. ^{1}H-^{1}H COSY spectrum of compound **8**

Figure S95. NOESY spectrum of compound **8**

Figure S96. HRESIMS spectrum of compound **8**

Figure S97. UV spectrum of compound **8**

Figure S98. IR spectrum of compound **8**

Figure S99. ^{1}H NMR (600 MHz, $CDCl_3$) spectrum of compound **9**

Figure S100. ^{13}C NMR (125 MHz, $CDCl_3$) spectrum of compound **9**

Figure S101. DEPT spectrum of compound **9**

Figure S102. HSQC spectrum of compound **9**

Figure S103. HMBC spectrum of compound **9**

Figure S104. ^{1}H-^{1}H COSY spectrum of compound **9**

Figure S105. NOESY spectrum of compound **9**

Figure S106. HRESIMS spectrum of compound **9**

Figure S107. UV spectrum of compound **9**

Figure S108. IR spectrum of compound **9**

Figure S1. The results of the DP4+ analysis of **1**

Functional	Solvent?	Basis Set	Type of Data
mPW1PW91	PCM	6-31+G(d,p)	Unscaled Shifts

		DP4+	91.85%	8.13%	0.00%	0.01%	-
Nuclei	sp2?	Experimenta	Isomer 1	Isomer 2	Isomer 3	Isomer 4	Isomer 5
C	x	125	123.8	124.8	124.5	123.3	
C	x	139.5	143.4	146.1	142.5	143.1	
C		80.4	82.4	83.2	83.0	83.7	
C		79.5	79.6	81.0	85.5	84.9	
C		35.4	39.8	40.6	38.6	38.8	
C		29	29.7	31.1	27.4	29.1	
C		11.9	13.4	13.6	14.4	12.0	
C		16.4	16.8	20.0	18.7	15.9	
C		14	17.3	15.9	20.8	17.6	
C		50.4	50.9	52.2	51.9	50.6	
H	x	6	6.4	6.4	6.4	6.5	
H	x	6.31	7.14	7.18	7.16	6.90	
H		3.39	3.38	3.53	3.56	3.25	
H		1.53	1.53	1.46	1.16	1.47	
H		1.38	1.52	1.55	1.55	1.82	
H		1.24	1.04	1.14	1.01	1.24	
H		0.85	0.82	0.86	0.71	0.83	
H		1.29	1.27	1.47	1.25	1.25	
H		0.9	0.96	1.01	0.99	0.65	
H		3.16	3.22	3.39	3.30	3.25	

	Isomer 1	Isomer 2	Isomer 3	Isomer 4	Isomer 5
sDP4+ (H data)	32.31%	41.14%	6.83%	19.72%	-
sDP4+ (C data)	42.52%	55.10%	1.52%	0.86%	-
sDP4+ (all data)	37.45%	61.81%	0.28%	0.46%	-
uDP4+ (H data)	94.52%	0.43%	0.08%	4.96%	-
uDP4+ (C data)	7.28%	85.10%	5.95%	1.67%	-
uDP4+ (all data)	93.78%	5.03%	0.07%	1.13%	-
DP4+ (H data)	96.33%	0.56%	0.02%	3.09%	-
DP4+ (C data)	6.18%	93.61%	0.18%	0.03%	-
DP4+ (all data)	91.85%	8.13%	0.00%	0.01%	-

Table S1. Experimental and calculated ^{13}C NMR chemical shifts of **1**

No.	Experimental (δ_C, ppm)	Calculated (δ_C, ppm)			
		(11*R*, 12*R*, 13*S*)-**1**	(11*S*, 12*R*, 13*S*)-**1**	(11*R*, 12*S*, 13S)-**1**	(11*S*, 12*S*, 13S)-**1**
9	125	123.8	124.8	124.5	123.3
10	139.5	143.4	146.1	142.5	143.1
11	80.4	82.4	83.2	83.0	83.7
12	79.5	79.6	81.0	85.5	84.9
13	35.4	39.8	40.6	38.6	38.8
14	29.0	29.7	31.1	27.4	29.1
15	11.9	13.4	13.6	14.4	12.0
17	16.4	16.8	20.0	18.7	15.9
18	14.0	17.3	15.9	20.8	17.6
OCH3	50.4	50.9	52.2	51.9	50.6
**R^2**		**0.9985**	**0.9982**	**0.9966**	**0.9975**
**MAE**		**1.78**	**2.73**	**3.00**	**2.21**
**CMAE**		**1.51**	**1.44**	**1.86**	**2.01**

Table S2. Experimental and calculated ^{1}H NMR chemical shifts of **1**

No.	Experimental (δ_H, ppm)	Calculated (δ_H, ppm)			
		(11*R*, 12*R*, 13*S*)-**1**	(11*S*, 12*R*, 13*S*)-**1**	(11*R*, 12*S*, 13S)-**1**	(11*S*, 12*S*, 13S)-**1**
9	6.00	6.40	6.43	6.37	6.52
10	6.31	7.14	7.18	7.16	6.90
12	3.39	3.38	3.53	3.56	3.25
13	1.53	1.53	1.46	1.16	1.47
14a	1.38	1.52	1.55	1.55	1.82
14b	1.24	1.04	1.14	1.01	1.24
15	0.85	0.82	0.86	0.71	0.83
17	1.29	1.27	1.47	1.25	1.25
18	0.90	0.96	1.01	0.99	0.65
OCH3	3.16	3.22	3.39	3.30	3.25
**R^2**		**0.9953**	**0.996**	**0.9934**	**0.992**
**MAE**		**0.17**	**0.23**	**0.26**	**0.22**
**CMAE**		**0.114**	**0.112**	**0.130**	**0.125**

Figure S2. Key MOs in important transitions of most populated conformer of **1**

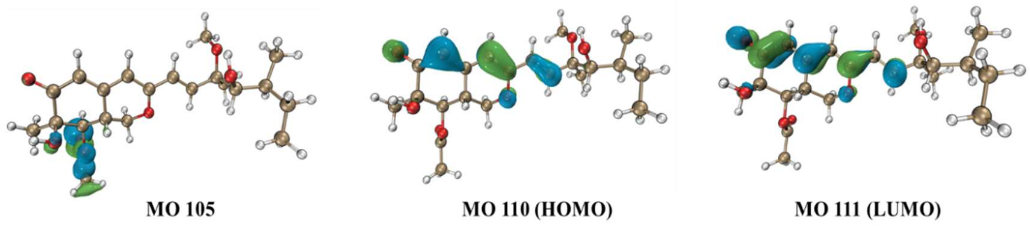

Figure S3. Linear regression analysis between experimental and calculated ^{13}C and ^{1}H NMR chemical shifts of isomers of **2**

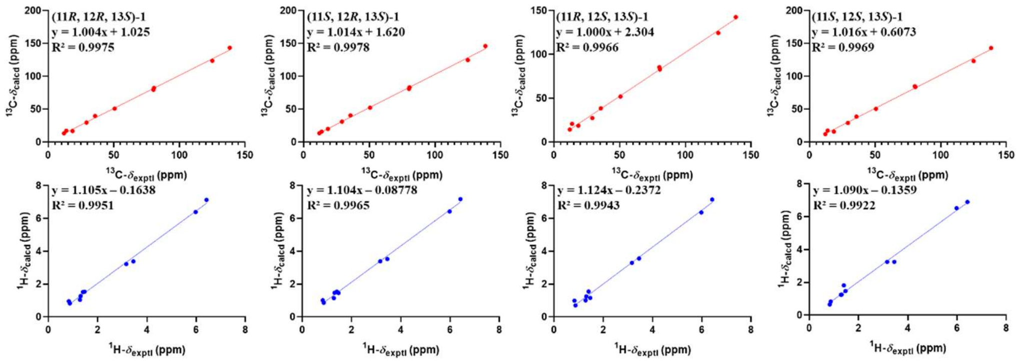

Figure S4. The results of the DP4+ analysis of **2**

Functional	Solvent?	Basis Set	Type of Data
mPW1PW91	PCM	6-31+G(d,p)	Unscaled Shifts

Nuclei	sp2?	xperimenta	Isomer 1	Isomer 2	Isomer 3	Isomer 4	Isomer 5
		DP4+	0.30%	99.67%	0.00%	0.03%	–
C	x	125	123.8	124.8	124.5	123.3	
C	x	138.4	143.4	146.1	142.5	143.1	
C		80.6	82.4	83.2	83.0	83.7	
C		80.2	79.6	81.0	85.5	84.9	
C		35.7	39.8	40.6	38.6	38.8	
C		29.2	29.7	31.1	27.4	29.1	
C		12	13.4	13.6	14.4	12.0	
C		18.5	16.8	20.0	18.7	15.9	
C		13.8	17.3	15.9	20.8	17.6	
C		50.6	50.9	52.2	51.9	50.6	
H	x	5.98	6.4	6.4	6.4	6.5	
H	x	6.42	7.14	7.18	7.16	6.90	
H		3.45	3.38	3.53	3.56	3.25	
H		1.47	1.53	1.46	1.16	1.47	
H		1.4	1.52	1.55	1.55	1.82	
H		1.28	1.04	1.14	1.01	1.24	
H		0.87	0.82	0.86	0.71	0.83	
H		1.31	1.27	1.47	1.25	1.25	
H		0.83	0.96	1.01	0.99	0.65	
H		3.16	3.22	3.39	3.30	3.25	

	Isomer 1	Isomer 2	Isomer 3	Isomer 4	Isomer 5
sDP4+ (H data)	13.30%	53.60%	6.40%	26.70%	–
sDP4+ (C data)	4.32%	94.48%	0.79%	0.41%	–
sDP4+ (all data)	1.12%	98.57%	0.10%	0.22%	–
uDP4+ (H data)	64.29%	3.10%	0.33%	32.28%	–
uDP4+ (C data)	1.22%	94.12%	3.53%	1.13%	–
uDP4+ (all data)	19.23%	71.52%	0.29%	8.96%	–
DP4+ (H data)	45.36%	8.80%	0.11%	45.72%	–
DP4+ (C data)	0.06%	99.90%	0.03%	0.01%	–
DP4+ (all data)	0.30%	99.67%	0.00%	0.03%	–

Table S3. Experimental and calculated ^{13}C NMR chemical shifts of **2**

No.	Experimental (δ_C, ppm)	Calculated (δ_C, ppm)			
		(11*R*, 12*R*, 13*S*)-**2**	(11*S*, 12*R*, 13*S*)-**2**	(11*R*, 12*S*, 13S)-**2**	(11*S*, 12*S*, 13S)-**2**
9	125	123.8	124.8	124.5	123.3
10	138.4	143.4	146.1	142.5	143.1
11	80.6	82.4	83.2	83.0	83.7
12	80.2	79.6	81.0	85.5	84.9
13	35.7	39.8	40.6	38.6	38.8
14	29.2	29.7	31.1	27.4	29.1
15	12	13.4	13.6	14.4	12.0
17	18.5	16.8	20.0	18.7	15.9
18	13.8	17.3	15.9	20.8	17.6
OCH3	50.6	50.9	52.2	51.9	50.6
**R^2**		**0.9975**	**0.9978**	**0.9966**	**0.9969**
**MAE**		**2.00**	**2.48**	**2.79**	**2.39**
**CMAE**		**1.85**	**1.41**	**2.02**	**2.17**

Table S4. Experimental and calculated ^{1}H NMR chemical shifts of **2**

No.	Experimental (δ_H, ppm)	Calculated (δ_H, ppm)			
		(11*R*, 12*R*, 13*S*)-**2**	(11*S*, 12*R*, 13*S*)-**2**	(11*R*, 12*S*, 13S)-**2**	(11*S*, 12*S*, 13S)-**2**
9	5.98	6.40	6.43	6.37	6.52
10	6.42	7.14	7.18	7.16	6.90
12	3.45	3.38	3.53	3.56	3.25
13	1.47	1.53	1.46	1.16	1.47
14a	1.4	1.52	1.55	1.55	1.82
14b	1.28	1.04	1.14	1.01	1.24
15	0.87	0.82	0.86	0.71	0.83
17	1.31	1.27	1.47	1.25	1.25
18	0.83	0.96	1.01	0.99	0.65
OCH3	3.16	3.22	3.39	3.30	3.25
**R^2**		**0.9951**	**0.9965**	**0.9943**	**0.9922**
**MAE**		**0.19**	**0.27**	**0.25**	**0.21**
**CMAE**		**0.116**	**0.102**	**0.124**	**0.114**

Figure S5. Linear regression analysis between experimental and calculated ^{13}C and ^{1}H NMR chemical shifts of isomers of **3**

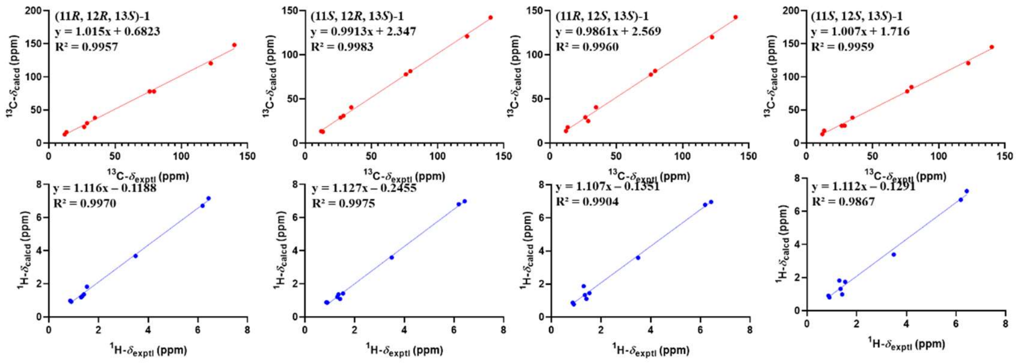

Figure S6. The results of the DP4+ analysis of **3**

Functional		Solvent?		Basis Set		Type of Data	
mPW1PW91		PCM		6-31+G(d,p)		Unscaled Shifts	

		DP4+	0.00%	100.00%	0.00%	0.00%	-
Nuclei	sp2?	xperimenta	Isomer 1	Isomer 2	Isomer 3	Isomer 4	Isomer 5
C	x	122.3	120.5	121.0	120.0	120.8	
C	x	140	148.2	142.3	142.8	145.2	
C		76.1	78.0	78.0	77.7	78.2	
C		79.4	78.1	81.6	81.9	84.9	
C		34.8	38.3	40.6	40.7	38.6	
C		28.9	30.3	31.1	25.1	26.4	
C		12	13.3	13.6	13.8	13.7	
C		26.7	24.5	29.0	29.3	26.3	
C		13.4	16.8	12.9	18.0	18.9	
H	x	6.19	6.7	6.8	6.8	6.7	
H	x	6.43	7.17	6.99	6.97	7.23	
H		3.49	3.68	3.59	3.59	3.40	
H		1.53	1.83	1.42	1.45	1.75	
H		1.41	1.36	1.09	1.10	0.99	
H		1.3	1.19	1.21	1.87	1.82	
H		0.9	0.92	0.87	0.77	0.81	
H		1.35	1.26	1.36	1.33	1.32	
H		0.86	0.98	0.88	0.87	0.91	

	Isomer 1	Isomer 2	Isomer 3	Isomer 4	Isomer 5
sDP4+ (H data)	12.54%	86.17%	1.27%	0.02%	-
sDP4+ (C data)	0.04%	99.73%	0.20%	0.03%	-
sDP4+ (all data)	0.01%	99.99%	0.00%	0.00%	-
uDP4+ (H data)	4.50%	93.53%	1.86%	0.11%	-
uDP4+ (C data)	0.09%	98.26%	1.56%	0.09%	-
uDP4+ (all data)	0.00%	99.96%	0.03%	0.00%	-
DP4+ (H data)	0.70%	99.28%	0.03%	0.00%	-
DP4+ (C data)	0.00%	100.00%	0.00%	0.00%	-
DP4+ (all data)	0.00%	100.00%	0.00%	0.00%	-

Table S5. Experimental and calculated ^{13}C NMR chemical shifts of **3**

No.	Experimental (δ_C, ppm)	Calculated (δ_C, ppm)			
		(11*R*, 12*R*, 13*S*)-**3**	(11*S*, 12*R*, 13*S*)-**3**	(11*R*, 12*S*, 13S)-**3**	(11*S*, 12*S*, 13S)-**3**
9	122.3	120.5	121.0	120.0	120.8
10	140	148.2	142.3	142.8	145.2
11	76.1	78.0	78.0	77.7	78.2
12	79.4	78.1	81.6	81.9	84.9
13	34.8	38.3	40.6	40.7	38.6
14	28.9	30.3	31.1	25.1	26.4
15	12	13.3	13.6	13.8	13.7
17	26.7	24.5	29.0	29.3	26.3
18	13.4	16.8	12.9	18.0	18.9
**R^2**		**0.9957**	**0.9983**	**0.996**	**0.9959**
**MAE**		**2.76**	**2.22**	**3.08**	**3.11**
**CMAE**		**2.37**	**1.32**	**2.19**	**2.45**

Table S6. Experimental and calculated ^{1}H NMR chemical shifts of **3**

No.	Experimental (δ_H, ppm)	Calculated (δ_H, ppm)			
		(11*R*, 12*R*, 13*S*)-**3**	(11*S*, 12*R*, 13*S*)-**3**	(11*R*, 12*S*, 13S)-**3**	(11*S*, 12*S*, 13S)-**3**
9	6.19	6.72	6.81	6.80	6.70
10	6.43	7.17	6.99	6.97	7.23
12	3.49	3.68	3.59	3.59	3.40
13	1.53	1.83	1.42	1.45	1.75
14a	1.41	1.36	1.09	1.10	0.99
14b	1.3	1.19	1.21	1.87	1.82
15	0.9	0.92	0.87	0.77	0.81
17	1.35	1.26	1.36	1.33	1.32
18	0.86	0.98	0.88	0.87	0.91
**R^2**		**0.997**	**0.9975**	**0.9904**	**0.9867**
**MAE**		**0.24**	**0.20**	**0.26**	**0.30**
**CMAE**		**0.106**	**0.083**	**0.142**	**0.225**

Figure S7. Linear regression analysis between experimental and calculated ^{13}C and ^{1}H NMR chemical shifts of isomers of **4**

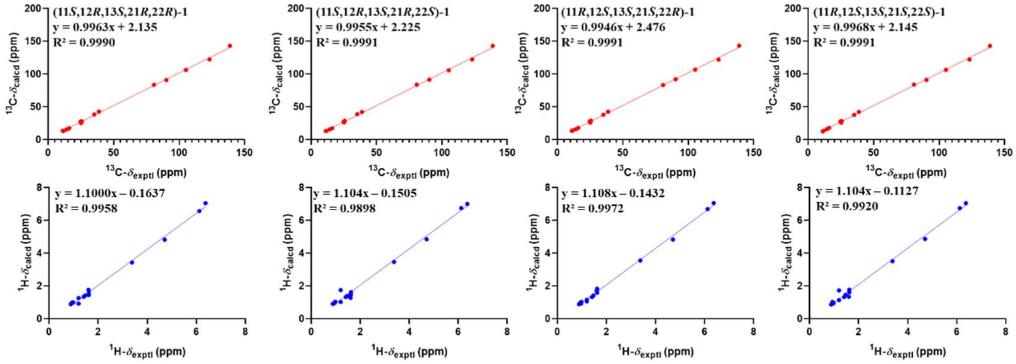

Figure S8. The results of the DP4+ analysis of **4**

Functional	Solvent?	Basis Set	Type of Data
mPW1PW91	PCM	6-31+G(d,p)	Unscaled Shifts

		DP4+	0.17%	0.00%	99.83%	0.00%	-
Nuclei	sp2?	xperimenta	Isomer 1	Isomer 2	Isomer 3	Isomer 4	Isomer 5
C	x	123.2	122.4	122.5	122.0	122.4	
C	x	138.9	143.0	142.9	143.2	143.0	
C		80.8	83.5	83.8	83.2	83.9	
C		90.4	91.0	91.4	92.0	91.1	
C		35.2	38.5	38.8	38.1	37.7	
C		25.6	28.1	28.8	28.9	28.5	
C		11.2	13.6	13.4	13.5	12.6	
C		25.2	25.3	26.3	25.8	25.9	
C		16	17.5	17.3	18.0	17.8	
C		105.3	106.3	105.8	106.8	106.4	
C		38.8	42.8	42.0	42.6	42.3	
C		24.8	27.55	26.94	27.95	27.96	
C		11.6	13.17	13.29	14.08	13.44	
C		14.1	15.63	15.59	15.72	15.73	
H	x	6.12	6.57	6.75	6.69	6.74	
H	x	6.37	7.05	7.00	7.06	7.05	
H		3.38	3.43	3.46	3.55	3.51	
H		1.47	1.42	1.40	1.42	1.46	
H		1.62	1.45	1.46	1.83	1.77	
H		1.2	0.92	1.02	1.06	1.14	
H		0.88	0.89	0.91	0.89	0.87	
H		1.41	1.34	1.32	1.32	1.32	
H		0.97	1.00	0.99	0.93	0.94	
H		4.71	4.82	4.85	4.83	4.87	
H		1.62	1.57	1.62	1.59	1.61	
H		1.6	1.761162544	1.266526707	1.707151765	1.339702434	
H		1.2	1.262498262	1.751178568	1.157815444	1.725450987	
H		0.93	0.984253303	0.949861895	0.968849965	1.020140488	
H		0.98	0.982969465	1.038283163	1.027742854	0.973494884	

	Isomer 1	Isomer 2	Isomer 3	Isomer 4	Isomer 5
sDP4+ (H data)	3.86%	0.03%	95.02%	1.08%	-
sDP4+ (C data)	8.23%	23.71%	42.59%	25.48%	-
sDP4+ (all data)	0.77%	0.02%	98.54%	0.67%	-
uDP4+ (H data)	72.36%	0.16%	26.96%	0.51%	-
uDP4+ (C data)	5.34%	10.45%	67.53%	16.69%	-
uDP4+ (all data)	17.41%	0.08%	82.13%	0.38%	-
DP4+ (H data)	9.83%	0.00%	90.15%	0.02%	-
DP4+ (C data)	1.22%	6.89%	80.05%	11.83%	-
DP4+ (all data)	0.17%	0.00%	99.83%	0.00%	-

Table S7. Experimental and calculated ^{13}C NMR chemical shifts of **4**

No.	Experimental (δ_C, ppm)	Calculated (δ_C, ppm)			
		**(11*S*,12*R*,13*S*,21*R*,22*R*)-4**	**(11*S*,12*R*,13*S*,21*R*,22*S*)-4**	**(11*R*,12*S*,13*S*,21*S*,22*R*)-4**	**(11*R*,12*S*,13*S*,21*S*,22*S*)-4**
9	123.2	122.4	122.5	122.0	122.4
10	138.9	143.0	142.9	143.2	143.0
11	80.8	83.5	83.8	83.2	83.9
12	90.4	91.0	91.4	92.0	91.1
13	35.2	38.5	38.8	38.1	37.7
14	25.6	28.1	28.8	28.9	28.5
15	11.2	13.6	13.4	13.5	12.6
17	25.2	25.3	26.3	25.8	25.9
18	16.0	17.5	17.3	18.0	17.8
21	105.3	106.3	105.8	106.8	106.4
22	38.8	42.8	42.0	42.6	42.3
23	24.8	27.6	26.9	28.0	28.0

24	11.6	13.2	13.3	14.1	13.4
25	14.1	15.6	15.6	15.7	15.7
**R^2**		**0.9990**	**0.9991**	**0.9991**	**0.9991**
**MAE**		**2.06**	**2.09**	**2.36**	**2.10**
**CMAE**		**1.23**	**1.10**	**1.21**	**1.16**

Table S8. Experimental and calculated ^{1}H NMR chemical shifts of **4**

No.	Experimental (δ_H, ppm)	Calculated (δ_H, ppm)			
		**(11*S*,12*R*,13*S*,21*R*,22*R*)-4**	**(11*S*,12*R*,13*S*,21*R*,22*S*)-4**	**(11*R*,12*S*,13*S*,21*S*,22*R*)-4**	**(11*R*,12*S*,13*S*,21*S*,22*S*)-4**
9	6.12	6.57	6.75	6.69	6.74
10	6.37	7.05	7.00	7.06	7.05
12	3.38	3.43	3.46	3.55	3.51
13	1.47	1.42	1.40	1.42	1.46
14a	1.62	1.45	1.46	1.83	1.77
14b	1.20	0.92	1.02	1.06	1.14
15	0.88	0.89	0.91	0.89	0.87
17	1.41	1.34	1.32	1.32	1.32
18	0.97	1.00	0.99	0.93	0.94
21	4.71	4.82	4.85	4.83	4.87
22	1.62	1.57	1.62	1.59	1.61
23a	1.60	1.76	1.27	1.71	1.34
23b	1.20	1.26	1.75	1.16	1.73
24	0.93	0.98	0.95	0.97	1.02
25	0.98	0.98	1.04	1.03	0.97
**R^2**		**0.9958**	**0.9898**	**0.9972**	**0.9820**
**MAE**		**0.15**	**0.20**	**0.16**	**0.19**
**CMAE**		**0.11**	**0.15**	**0.10**	**0.12**

Figure S9. ^{1}H-^{1}H COSY (blue bold lines) and key HMBC (red arrows) correlations of compound **2**

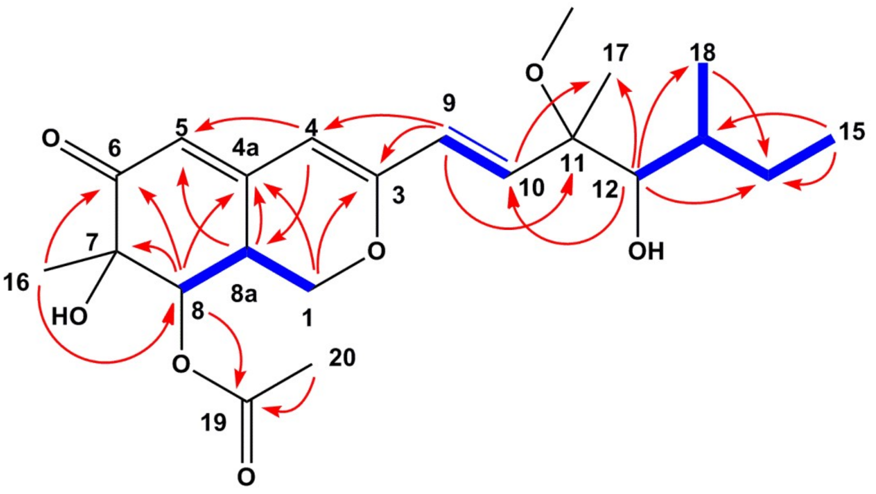

Figure S10. The key NOESY correlations of compound **2**

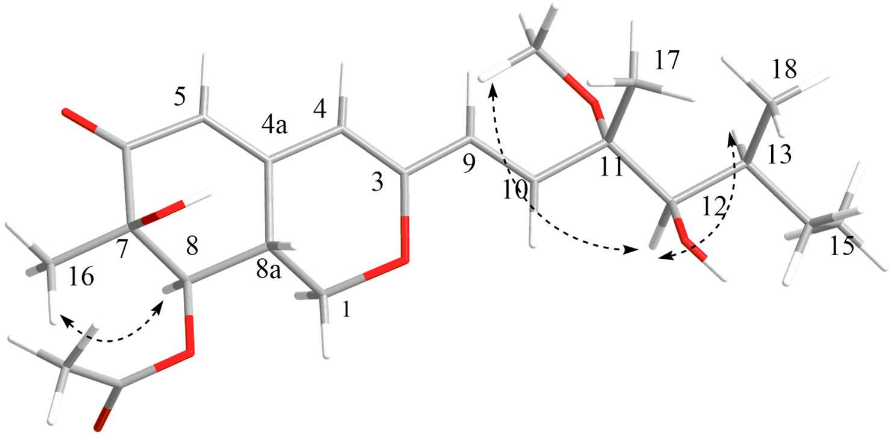

Figure S11. ^{1}H-^{1}H COSY (blue bold lines) and key HMBC (red arrows) correlations of compound **3**

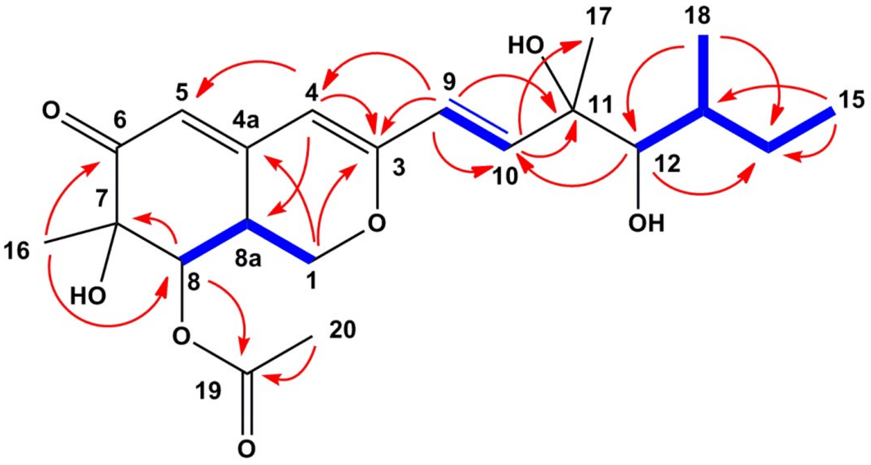

Figure S12. The key NOESY correlations of compound **3**

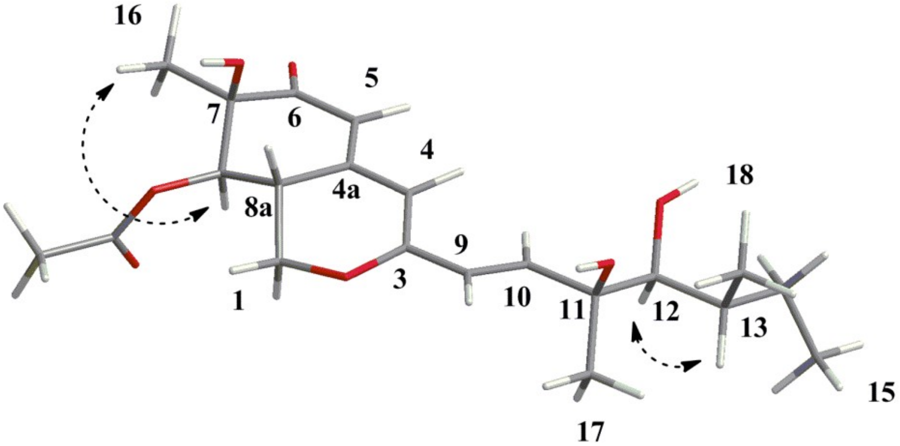

Figure S13 ^{1}H-^{1}H COSY (blue bold lines) and key HMBC (red arrows) correlations of compound **5**

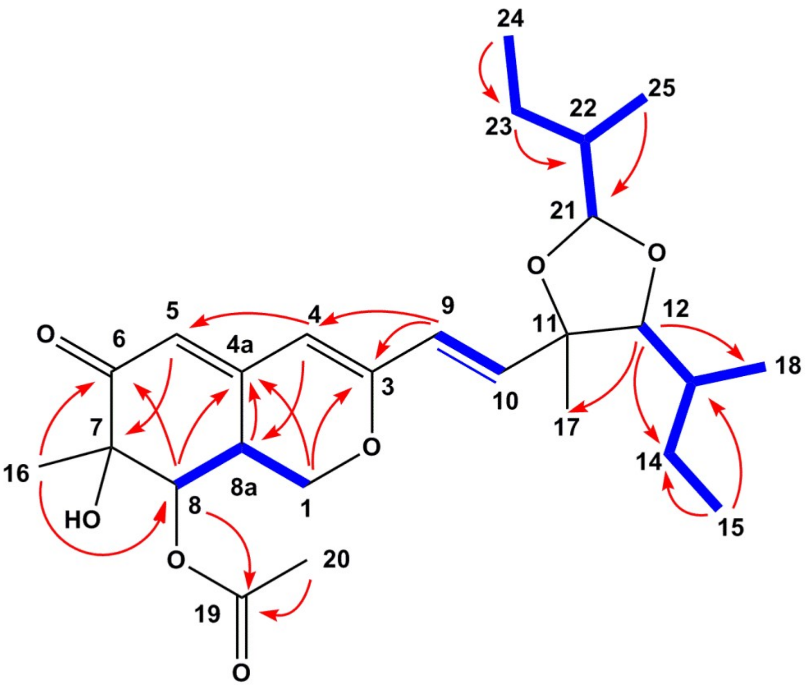

Figure S14. The key NOESY correlations of compound **5**

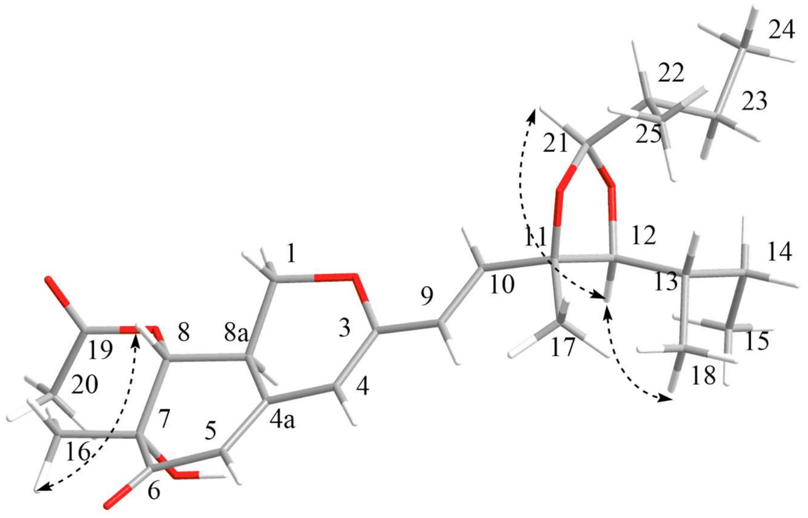

Figure S15. ^{1}H-^{1}H COSY (blue bold lines) and key HMBC (red arrows) correlations of compound **7**

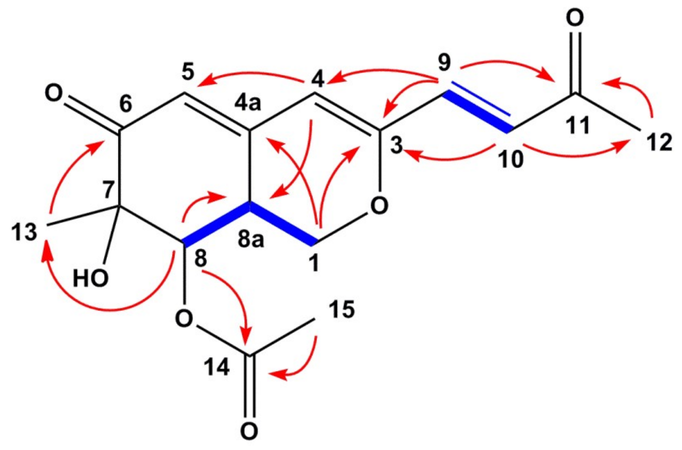

Figure S16. The key NOESY correlations of compound **7**

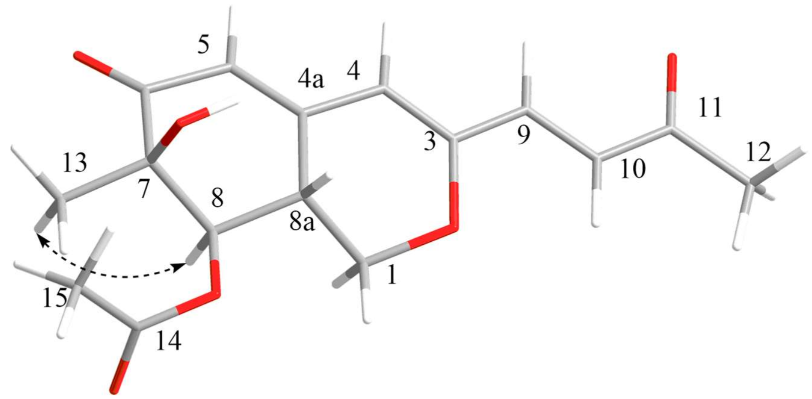

Figure S17. ^{1}H-^{1}H COSY (blue bold lines) and key HMBC (red arrows) correlations of compound **8**

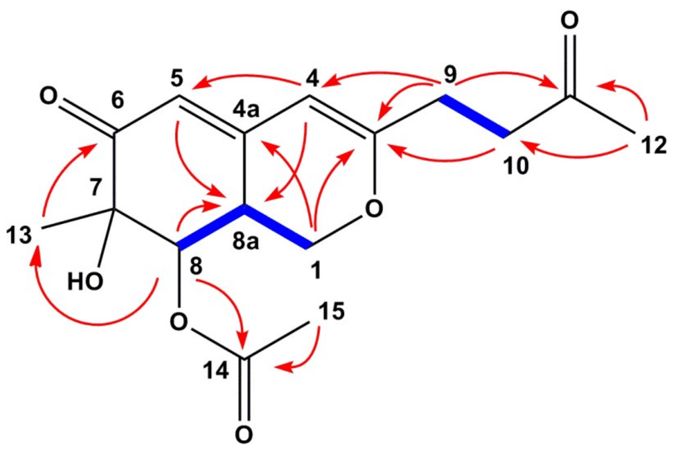

Figure S18. The key NOESY correlations of compound **8**

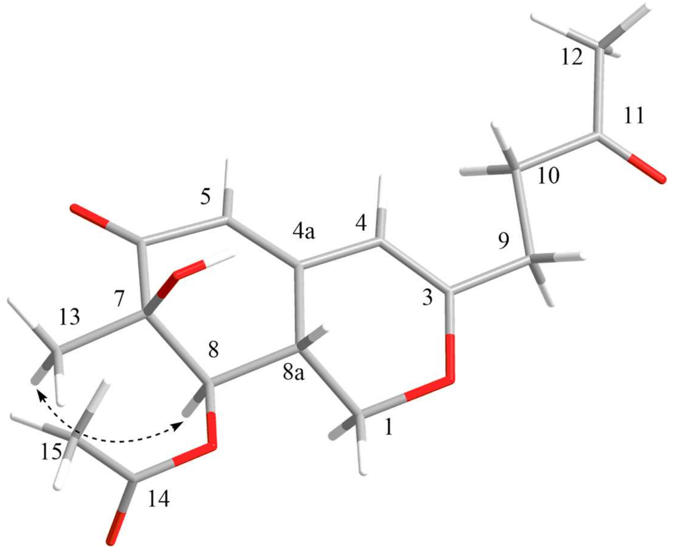

Figure S19. ^{1}H NMR (600 MHz, $CDCl_3$) spectrum of compound **1**

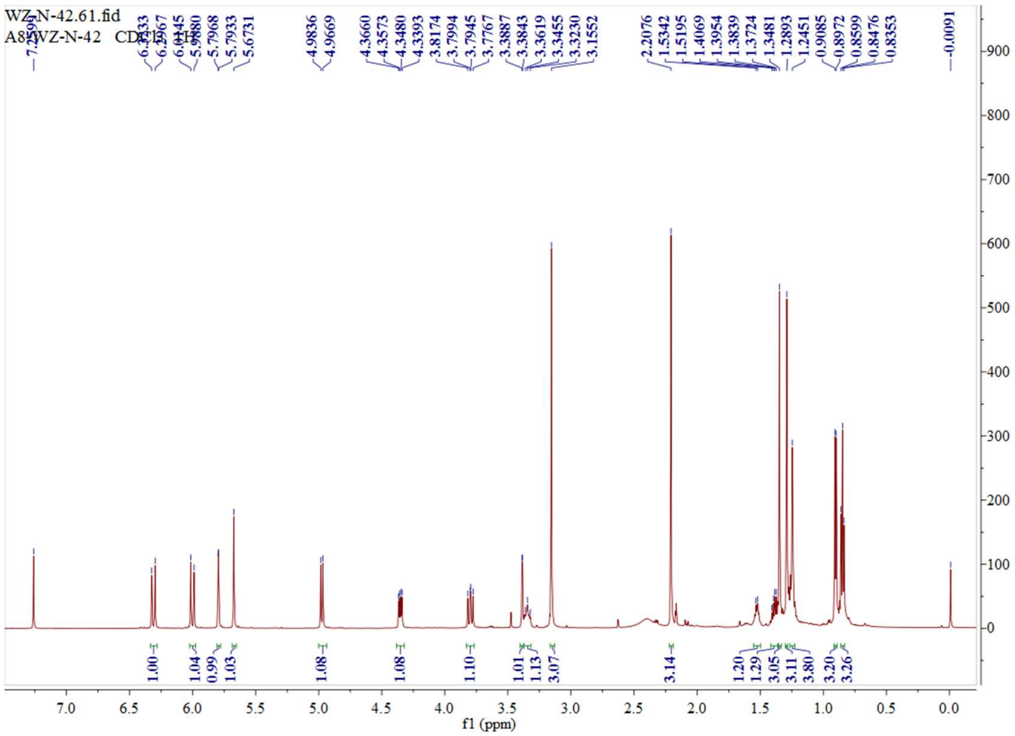

Figure S20. ^{13}C NMR (125 MHz, $CDCl_3$) spectrum of compound **1**

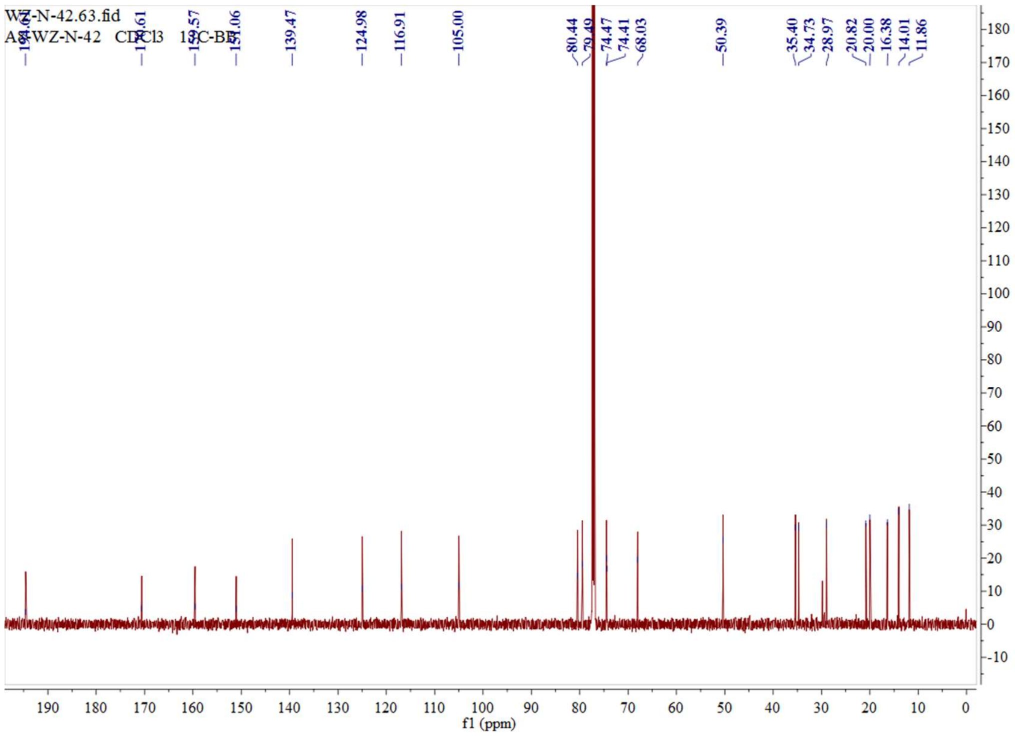

Figure S21. DEPT spectrum of compound **1**

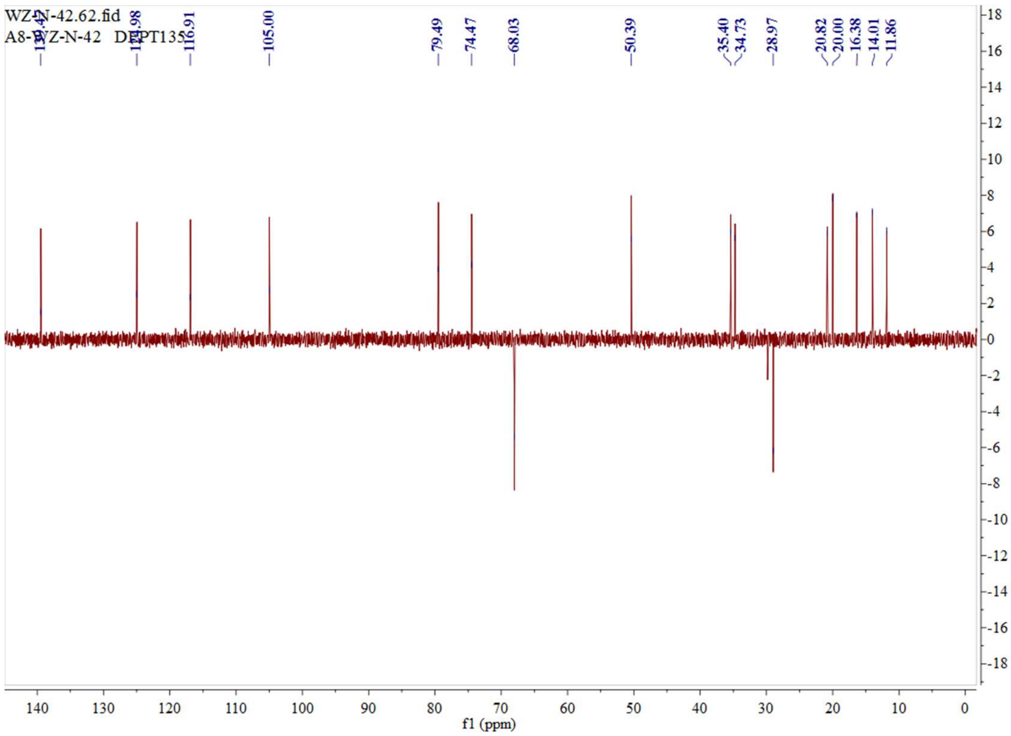

Figure S22. HSQC spectrum of compound **1**

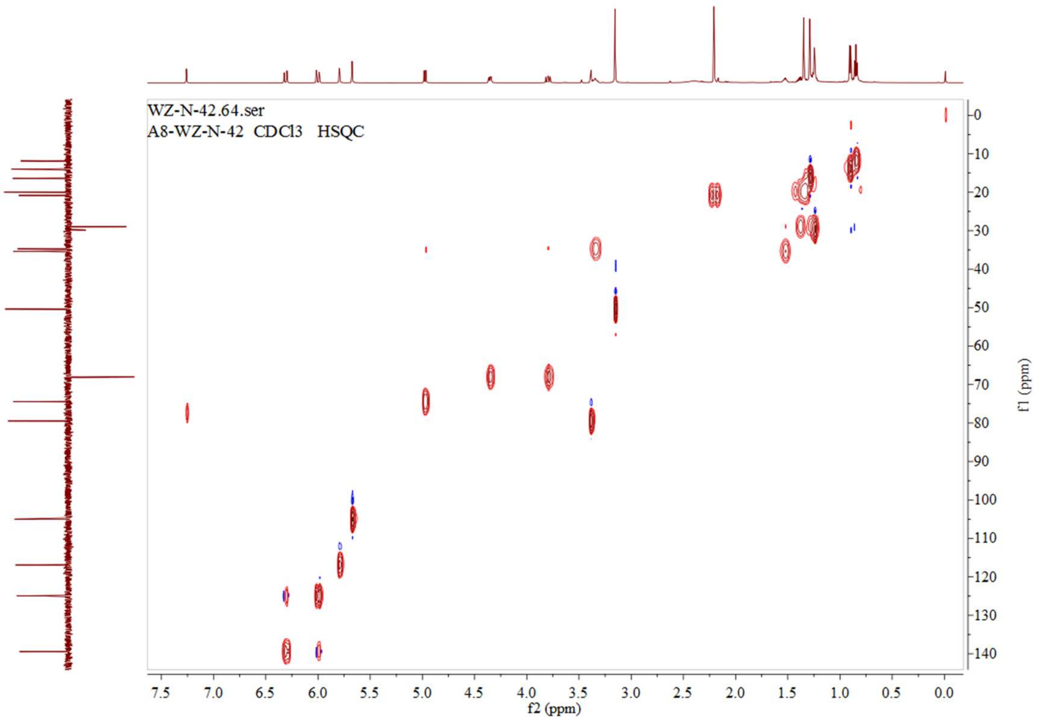

Figure S23. HMBC spectrum of compound **1**

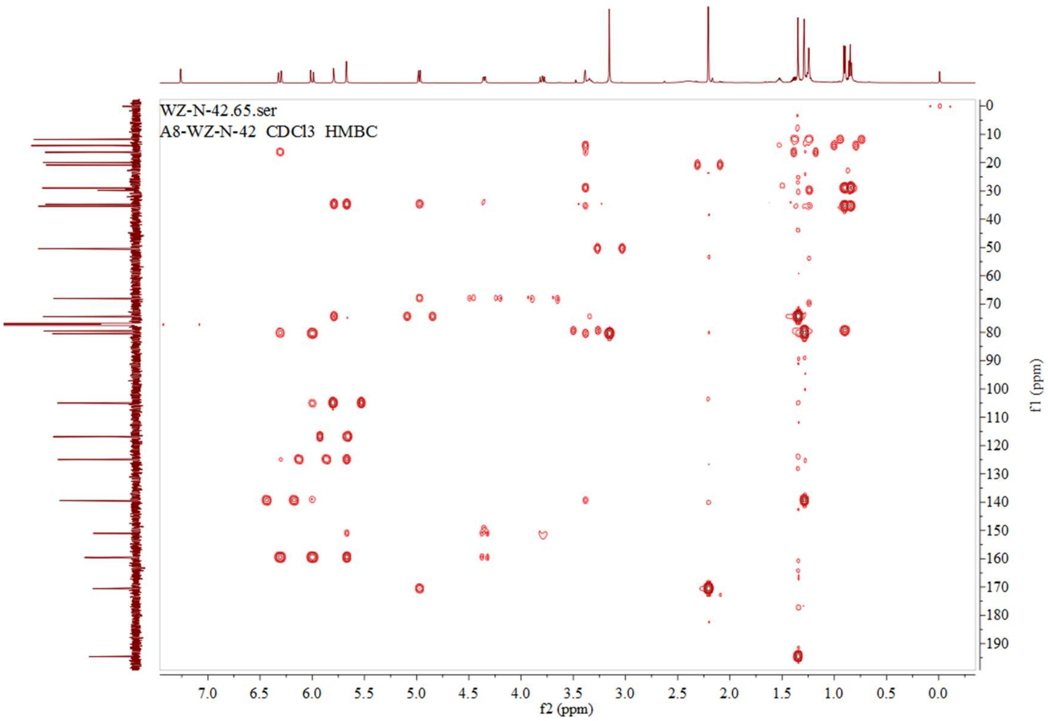

Figure S24. ^{1}H-^{1}H COSY spectrum of compound **1**

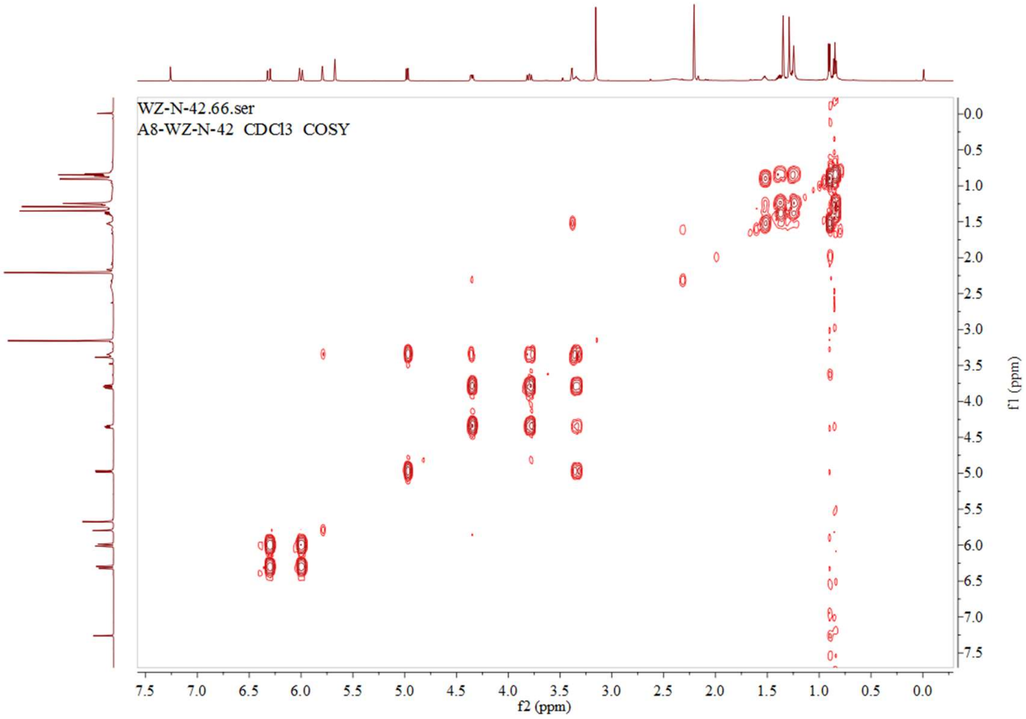

Figure S25. NOESY spectrum of compound **1**

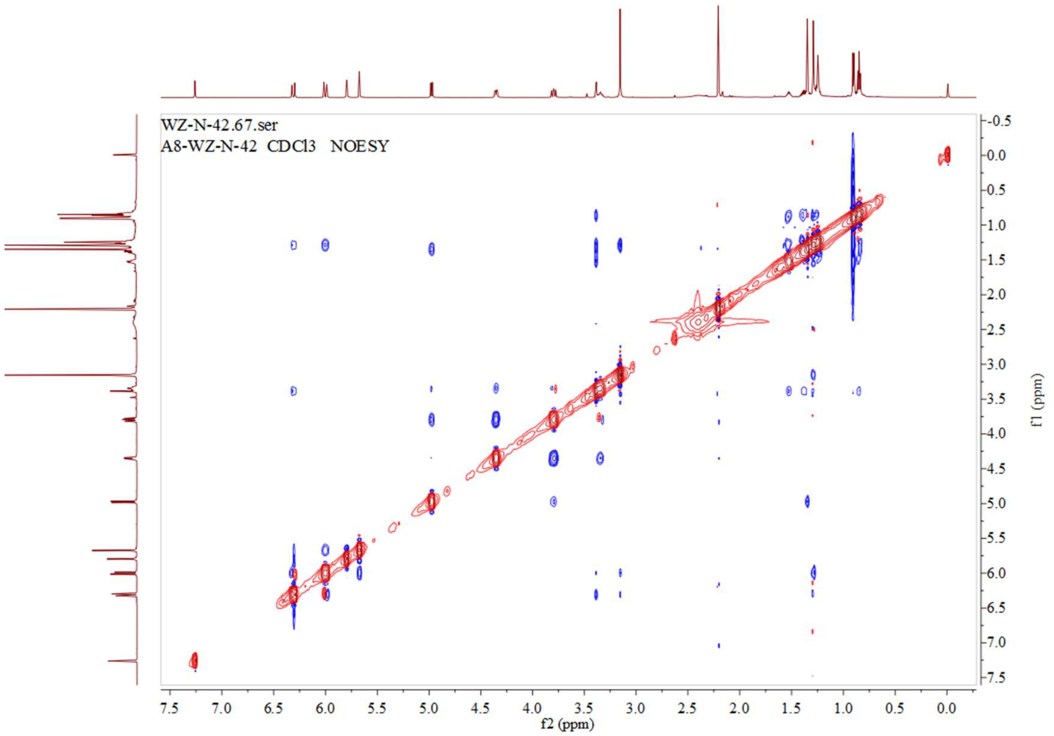

Figure S26. HRESIMS spectrum of compound **1**

Qualitative Analysis Report

**Data Filename**	ESIH202104664.d	**Sample Name**	A8-A8-WZ-N-42
**Sample ID**		**Position**	P1-D2
**Instrument Name**	Agilent G6520 Q-TOF	**Acq Method**	20160322_MS_ESIH_POS_1min.m
**Acquired Time**	10/20/2021 20:32:53	**IRM Calibration Status**	Success
**DA Method**	small molecular data analysis method.m	**Comment**	ESIH by zhuzhenyun

User Spectra

Fragmentor Voltage	Collision Energy	Ionization Mode
175	0	ESI

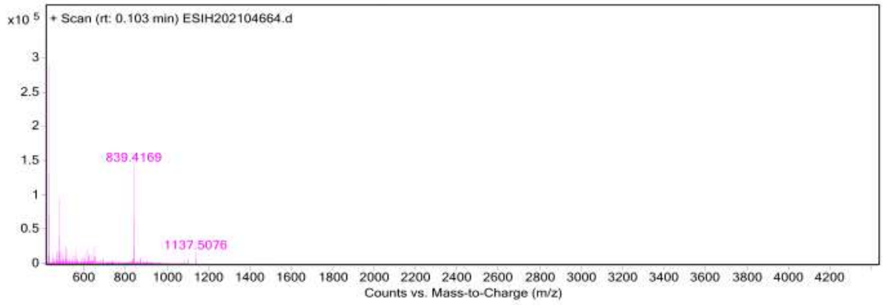

Formula Calculator Results

m/z	Calc m/z	Diff (mDa)	Diff (ppm)	Ion Formula	Ion
431.2038	431.204	0.24	0.55	C22 H32 Na O7	(M+Na)+

--- End Of Report ---

Figure S27. UV spectrum of compound **1**

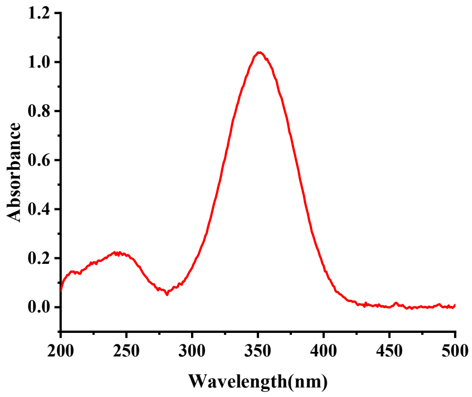

Figure S28. IR spectrum of compound **1**

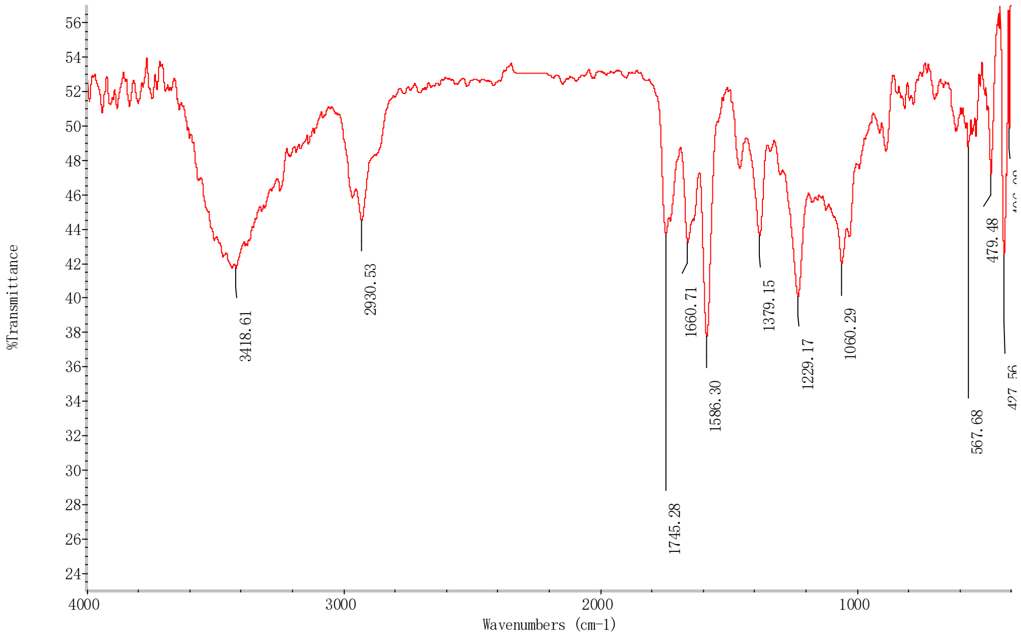

Figure S29. ^{1}H NMR (600 MHz, $CDCl_3$) spectrum of compound **2**

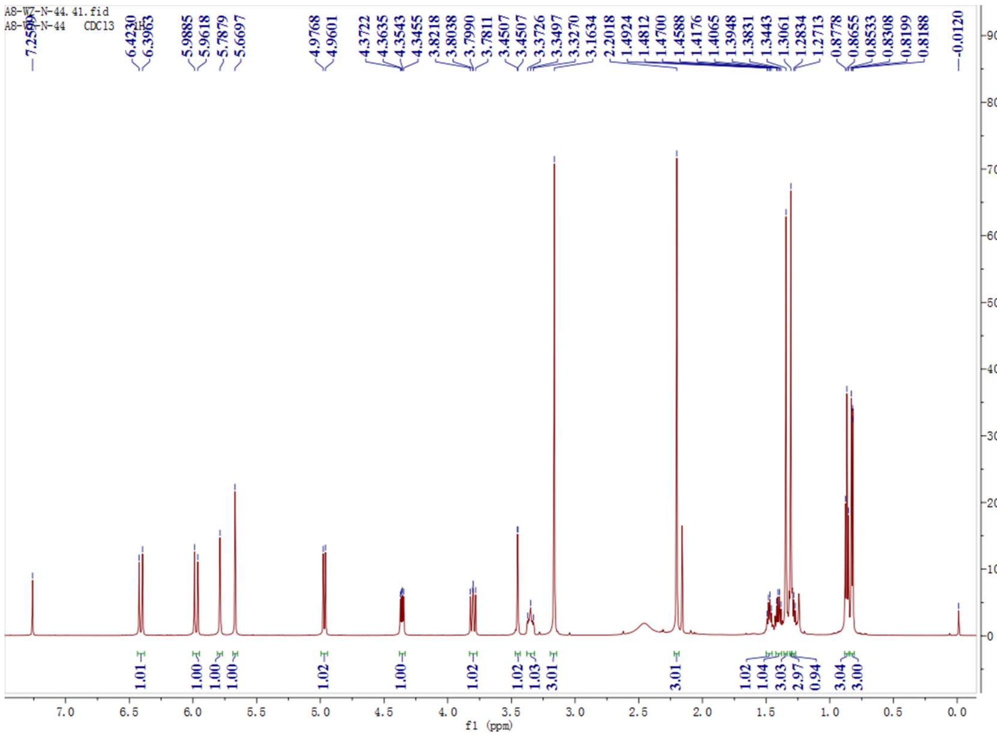

Figure S30. ^{13}C NMR (125 MHz, $CDCl_3$) spectrum of compound **2**

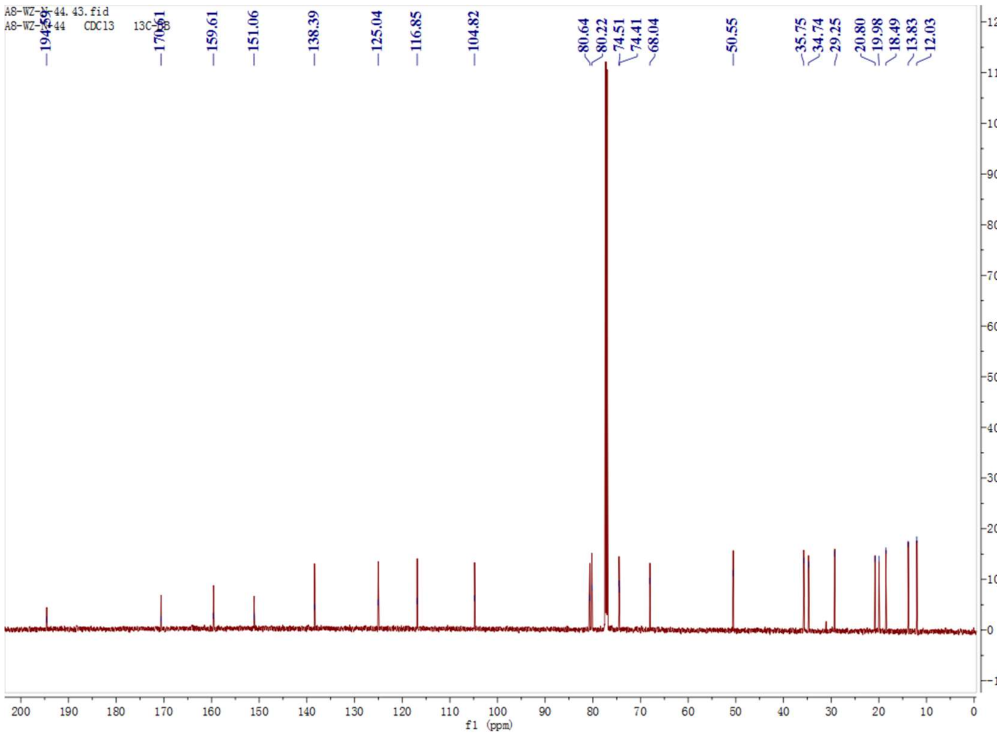

Figure S30. DEPT spectrum of compound **2**

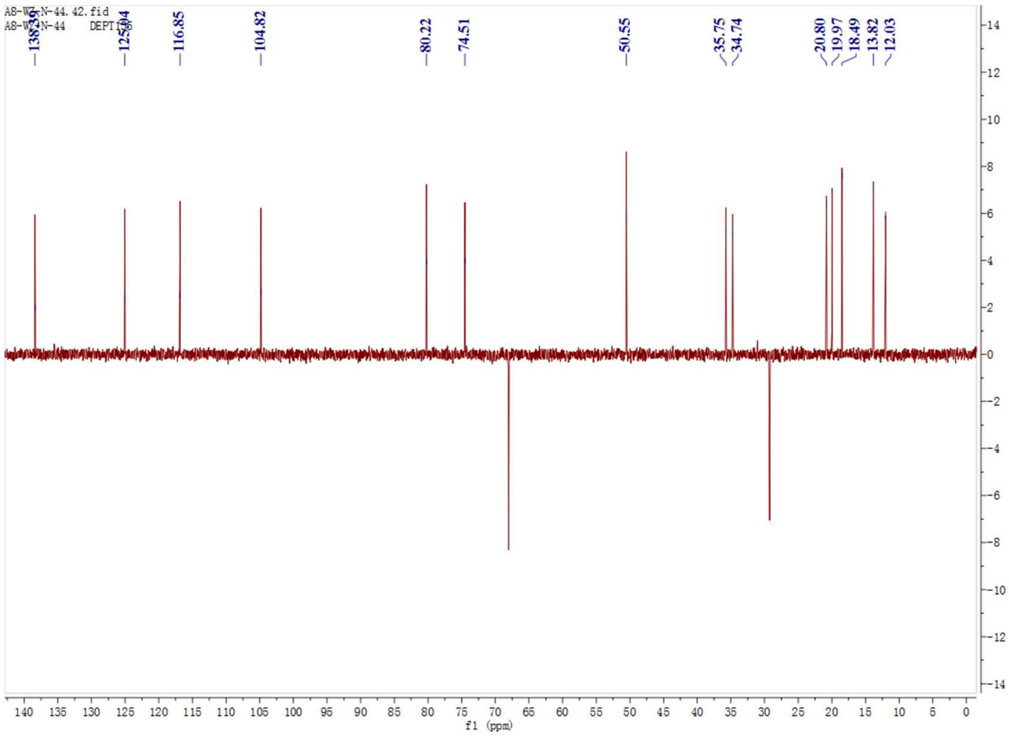

Figure S32. HSQC spectrum of compound **2**

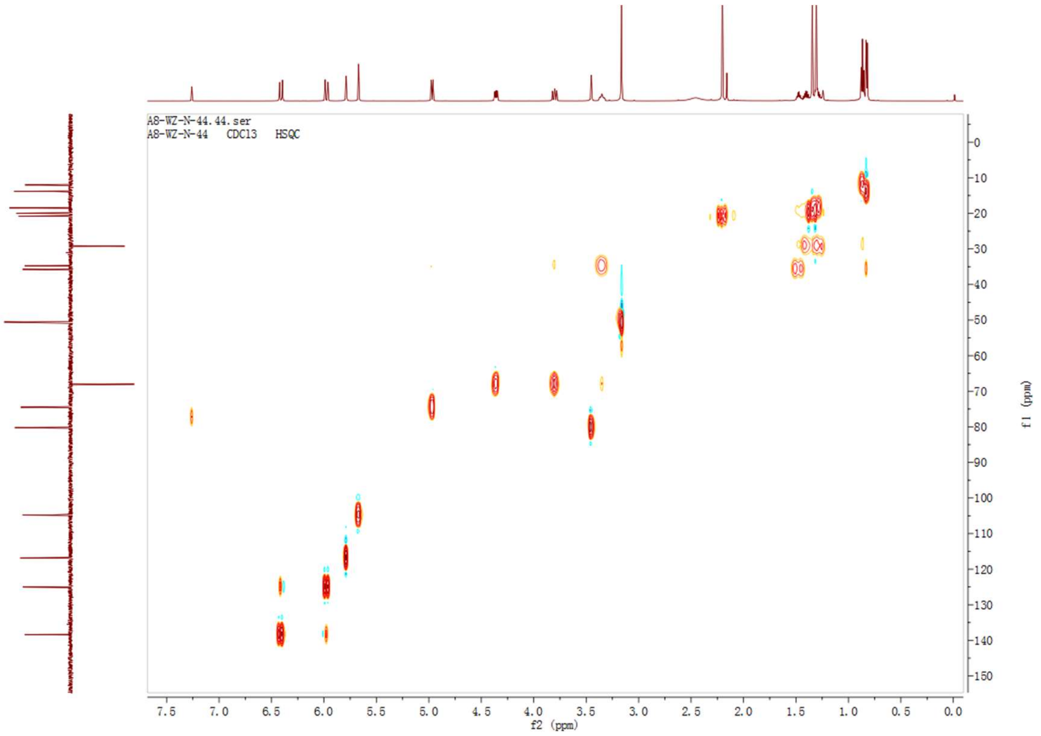

Figure S33. HMBC spectrum of compound **2**

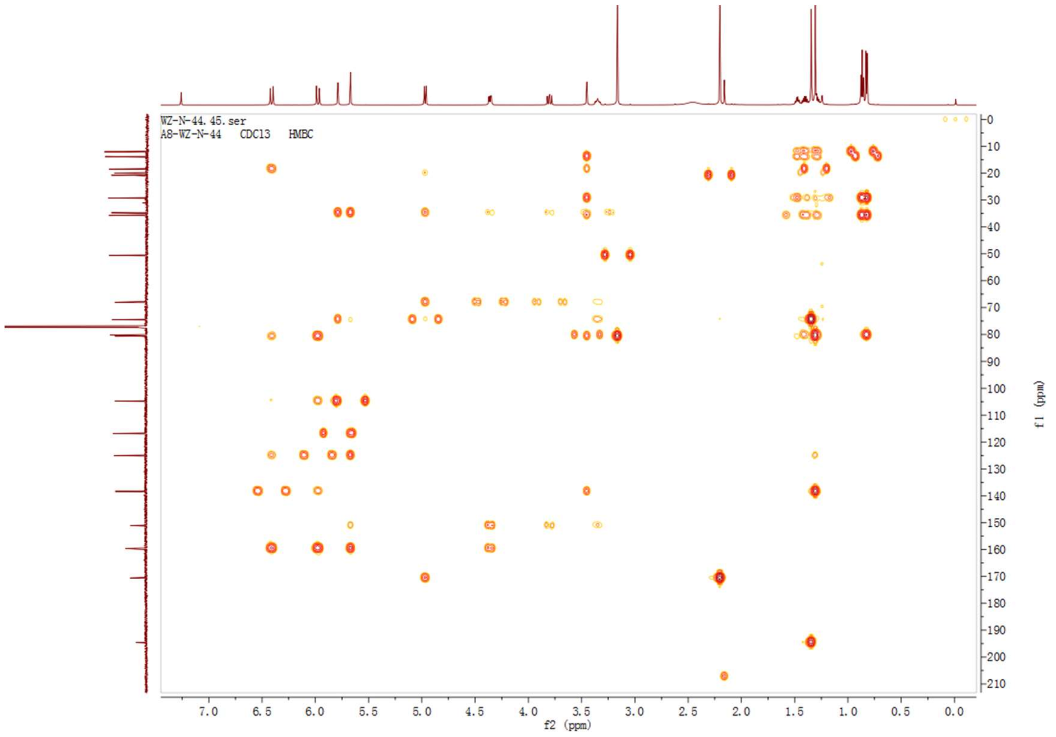

Figure S34. ^{1}H-^{1}H COSY spectrum of compound **1**

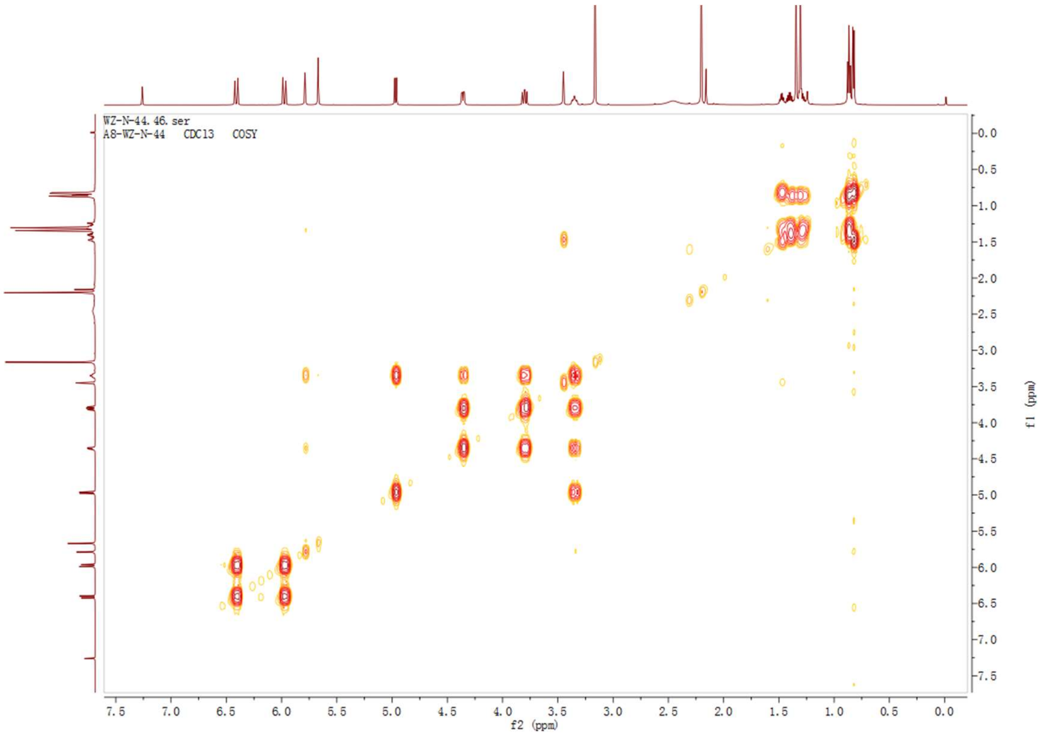

Figure S35. NOESY spectrum of compound **2**

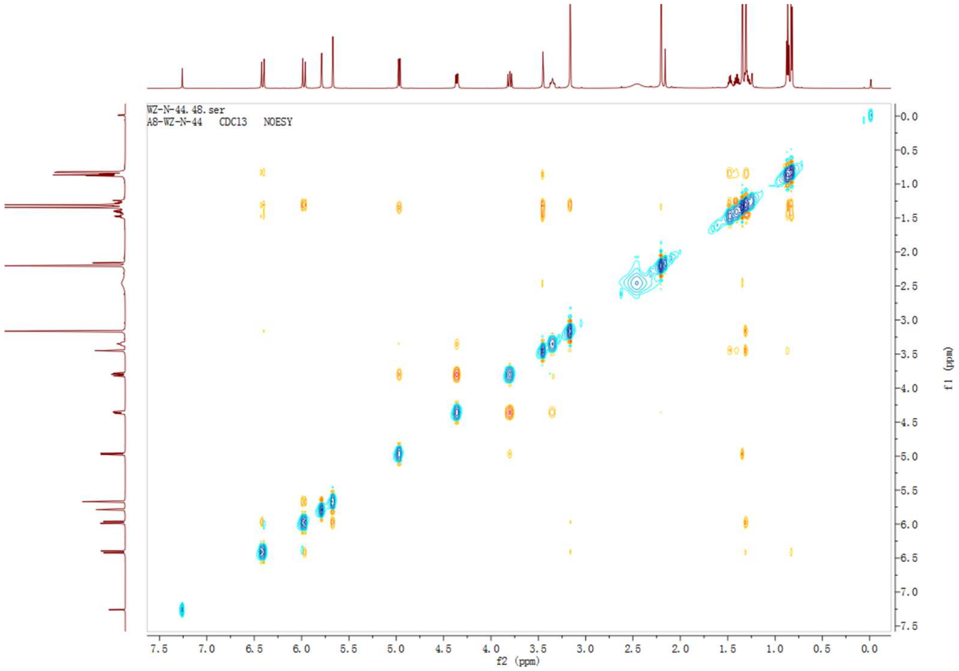

Figure S36. HRESIMS spectrum of compound **2**

Mass Spectrum SmartFormula Report

Analysis Info

Analysis Name	D:\Data\A501\WYL\2022\20220517\B502-WZ-N-44_GB5_01_4300.d	Acquisition Date	2022-05-17 10:15:12
Method	lc-ms_as_ms-0.5MIN.m	Operator	Demo User
Sample Name	B502-WZ-N-44	Instrument	compact 8255754.20156
Comment			

Acquisition Parameter

Source Type	ESI	Ion Polarity	Positive	Set Nebulizer	1.8 Bar
Focus	Not active	Set Capillary	3500 V	Set Dry Heater	220 °C
Scan Begin	50 m/z	Set End Plate Offset	-500 V	Set Dry Gas	4.0 l/min
Scan End	2500 m/z	Set Charging Voltage	2000 V	Set Divert Valve	Waste
		Set Corona	0 nA	Set APCI Heater	0 °C

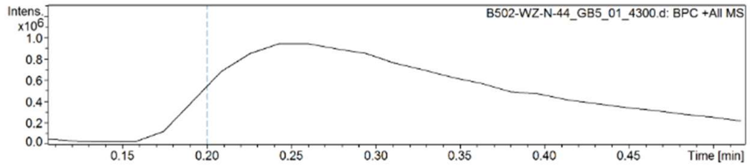

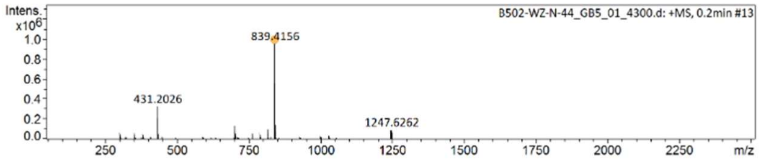

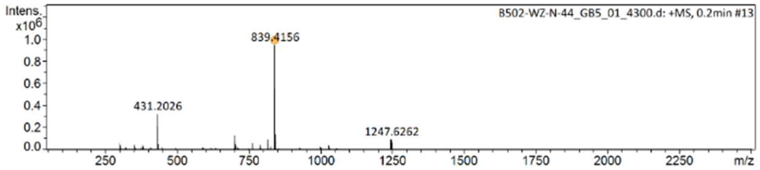

Meas. m/z	#	Ion Formula	m/z	err [ppm]	mSigma	# mSigma	Score	rdb	e⁻ Conf	N-Rule	Adduct
839.4156	1	C44H64NaO14	839.4188	3.8	10.4	1	100.00	13.0	even	ok	2M+Na

Figure S37. UV spectrum of compound **2**

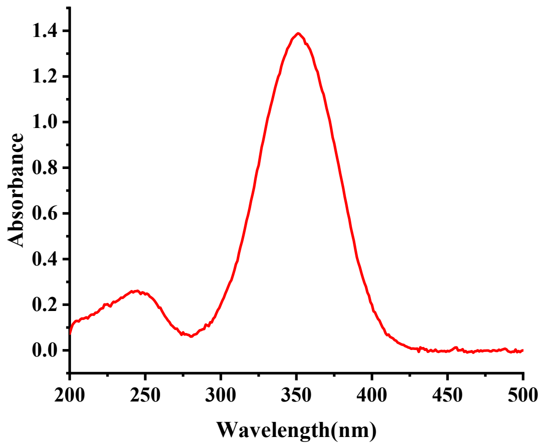

Figure S38. IR spectrum of compound **2**

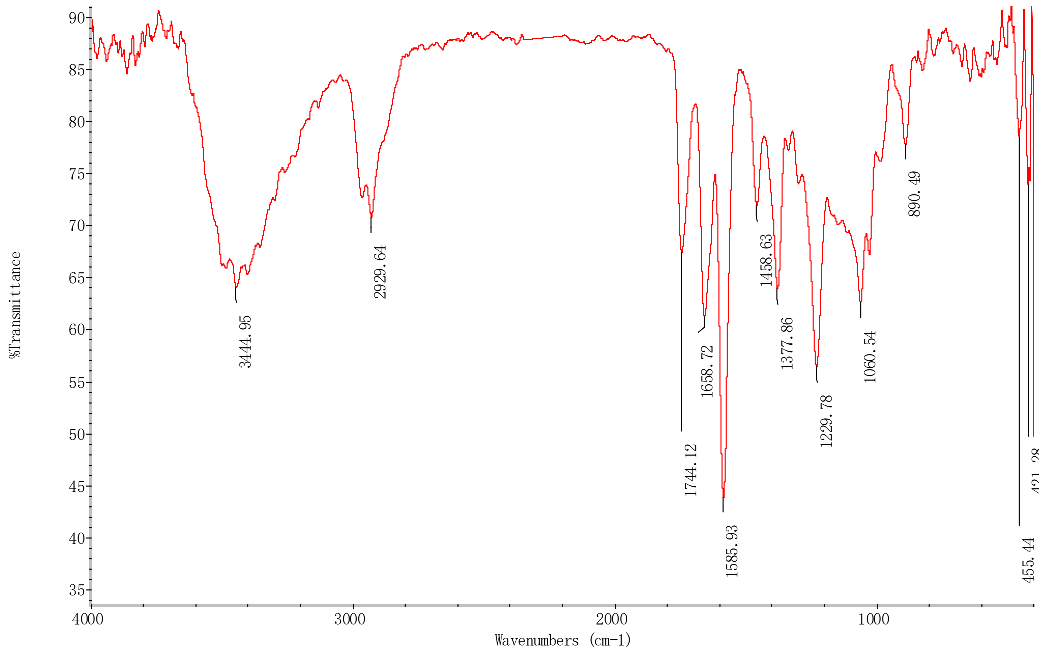

Figure S39. ^{1}H NMR (400 MHz, $CDCl_3$) spectrum of compound **3**

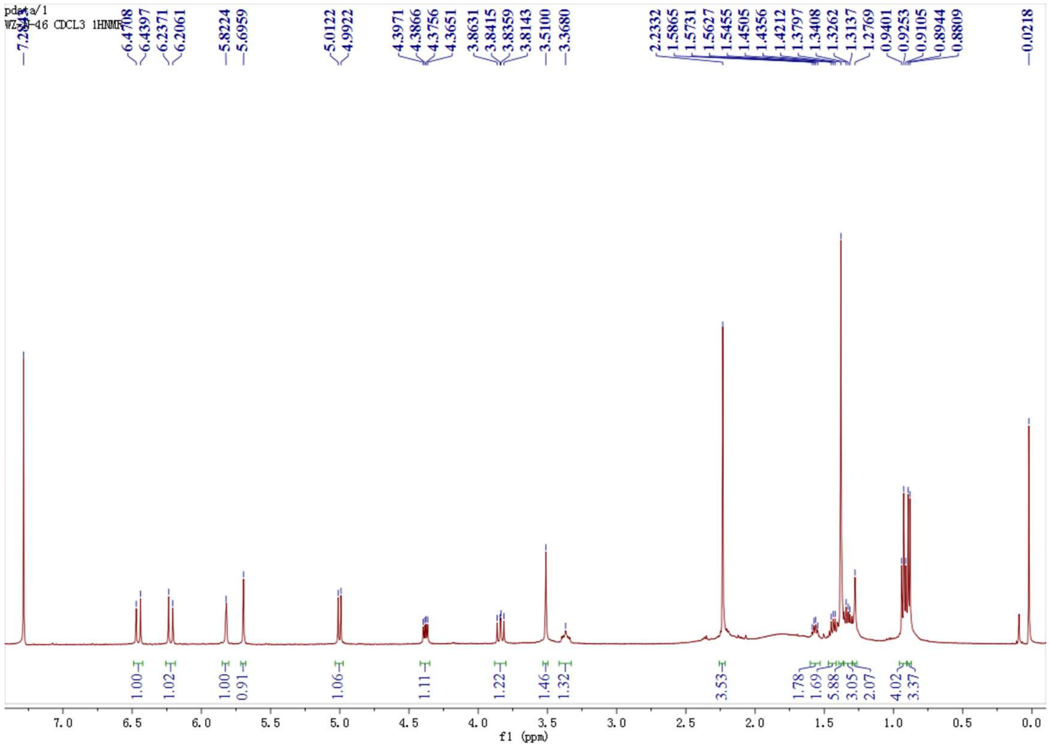

Figure S40. ^{13}C NMR (125 MHz, $CDCl_3$) spectrum of compound **3**

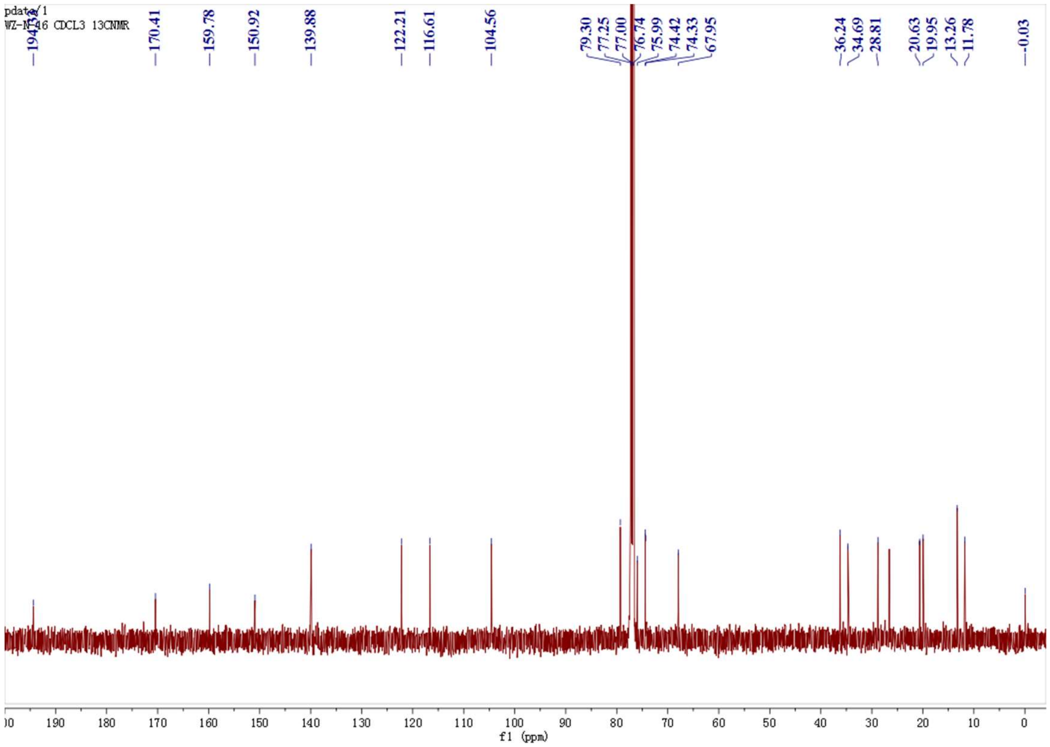

Figure S41. DEPT spectrum of compound **3**

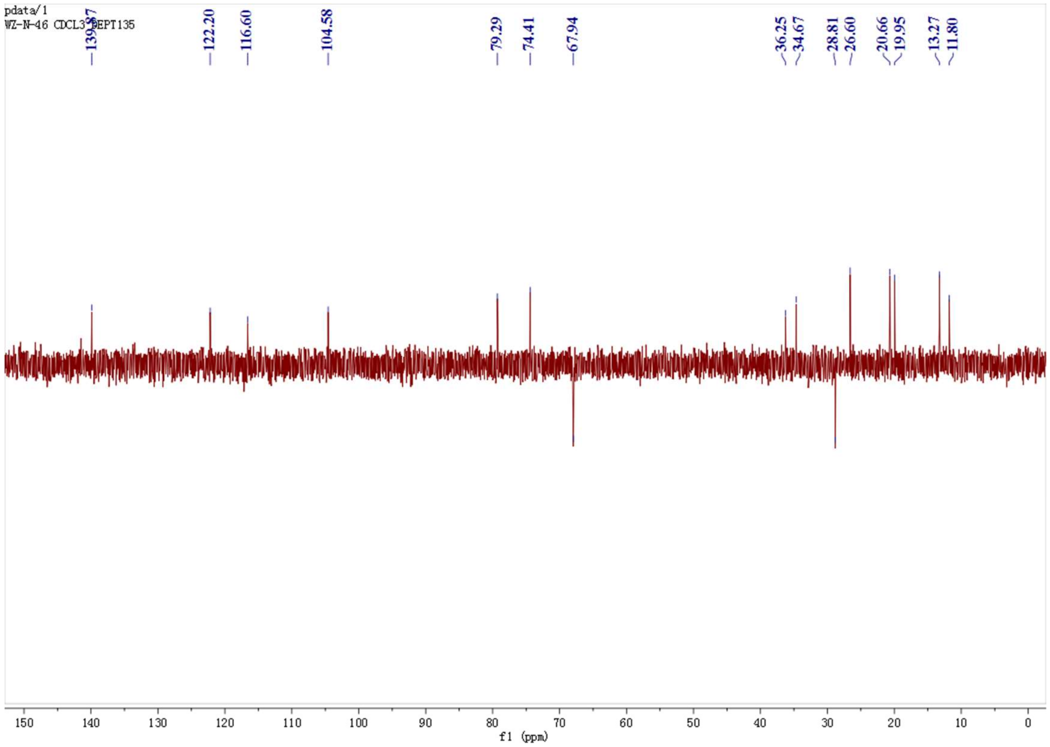

Figure S42. HSQC spectrum of compound **3**

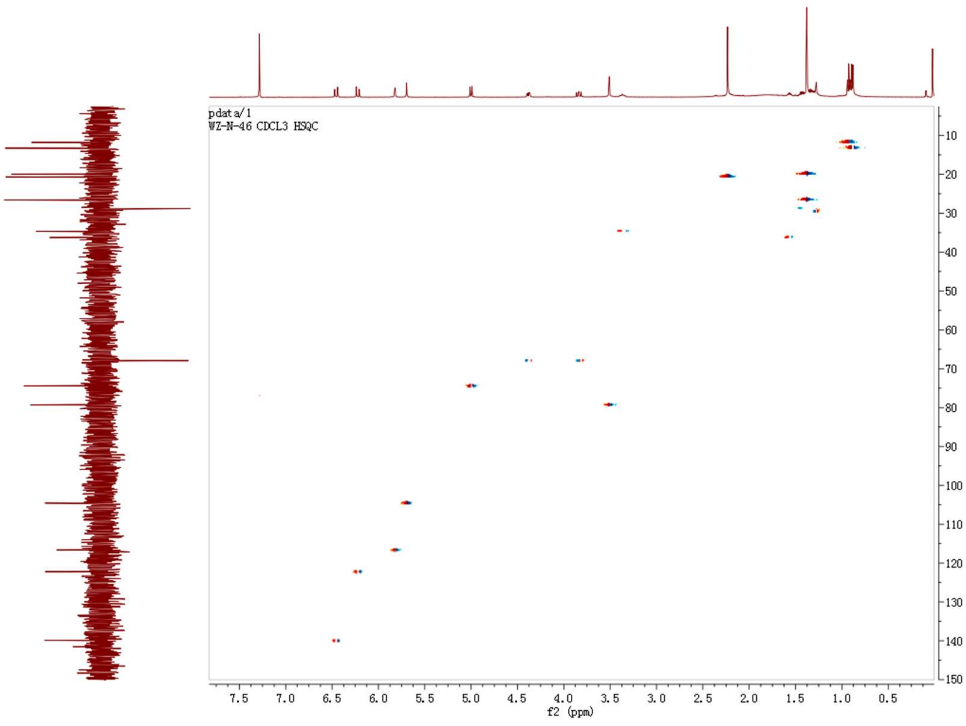

Figure S43. HMBC spectrum of compound **3**

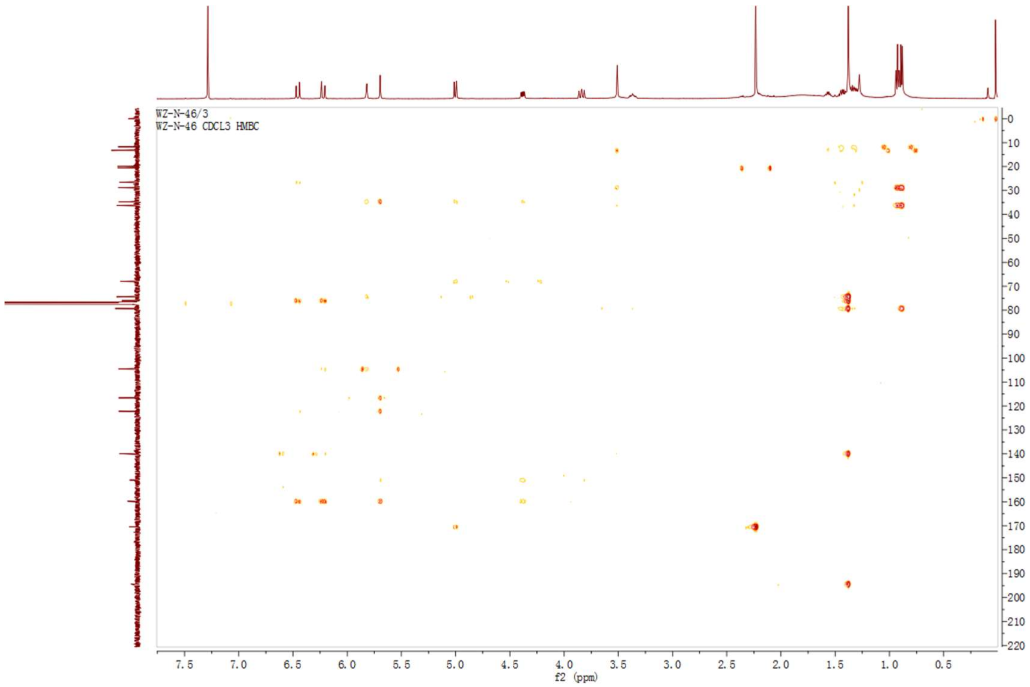

Figure S44. ^{1}H-^{1}H COSY spectrum of compound **3**

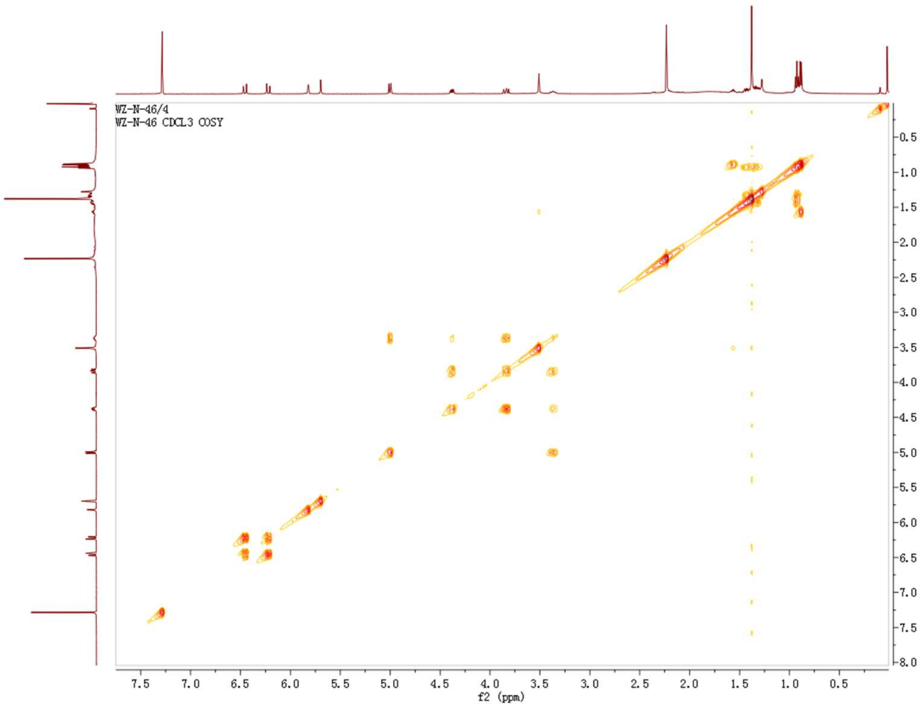

Figure S45. ROESY spectrum of compound **3**

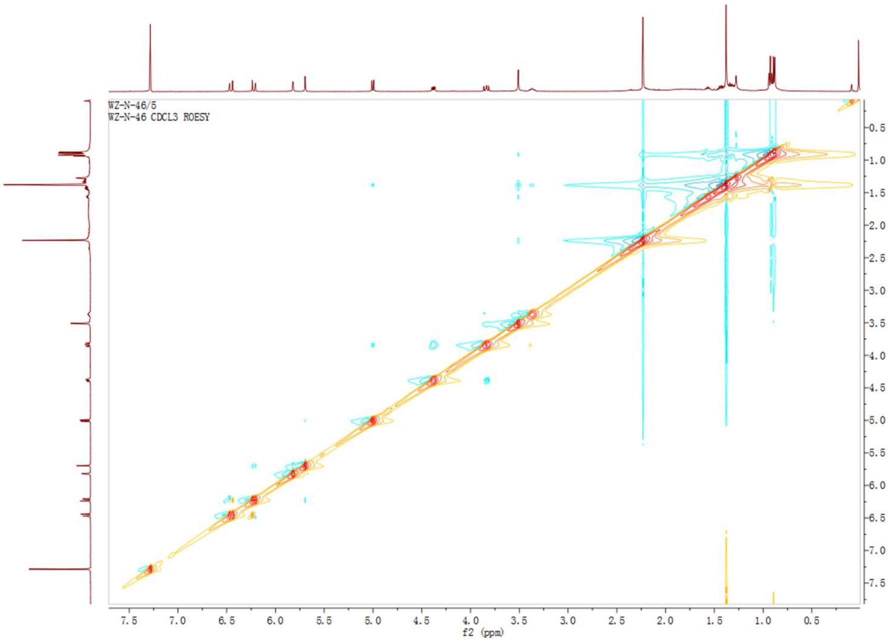

Figure S46. HRESIMS spectrum of compound **3**

Mass Spectrum SmartFormula Report

Analysis Info

Analysis Name	D:\Data\A501\WYL\2022\20220915\B502-WZ-N-46_BB5_01_4639.d	Acquisition Date	2022-09-16 16:56:48
Method	lc-ms_as_ms-0.5MIN.m	Operator	Demo User
Sample Name	B502-WZ-N-46	Instrument	compact 8255754.20156
Comment			

Acquisition Parameter

Source Type	ESI	Ion Polarity	Positive	Set Nebulizer	1.8 Bar
Focus	Not active	Set Capillary	3500 V	Set Dry Heater	220 °C
Scan Begin	50 m/z	Set End Plate Offset	-500 V	Set Dry Gas	4.0 l/min
Scan End	2500 m/z	Set Charging Voltage	2000 V	Set Divert Valve	Waste
		Set Corona	0 nA	Set APCI Heater	0 °C

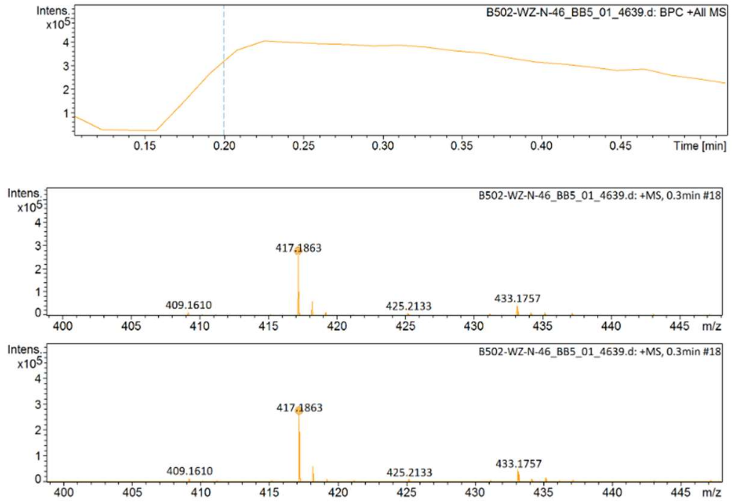

Meas. m/z	#	Ion Formula	m/z	err [ppm]	mSigma	# mSigma	Score	rdb	e⁻ Conf	N-Rule	Adduct
417.1863	1	C21H30NaO7	417.1884	5.1	5.8	1	100.00	7.0	even	ok	M+Na

Figure S47. UV spectrum of compound **3**

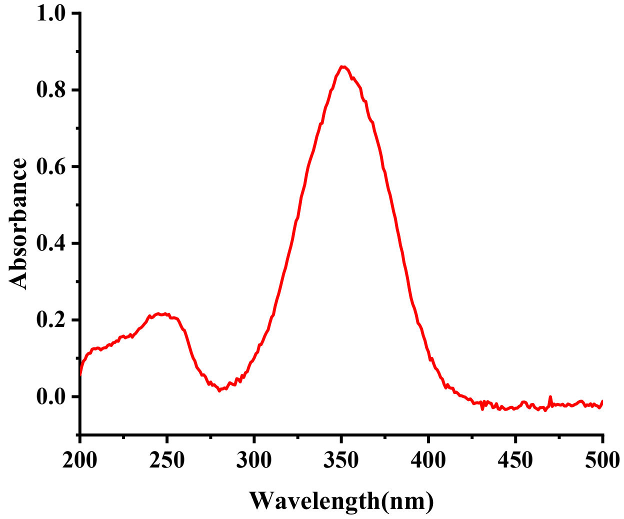

Figure S48. IR spectrum of compound **3**

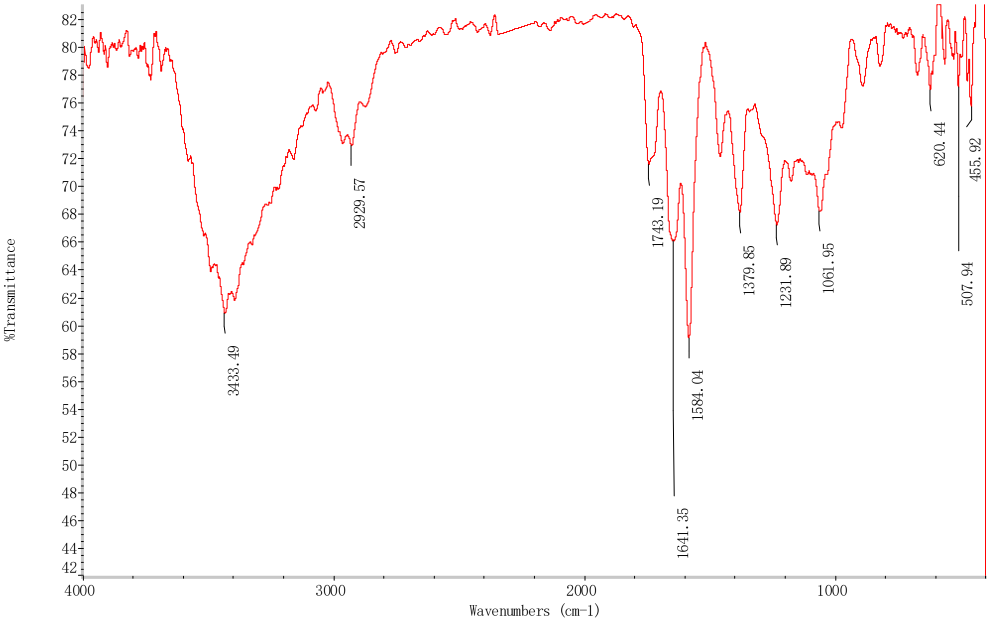

Figure S49. ^{1}H NMR (600 MHz, $CDCl_3$) spectrum of compound **4**

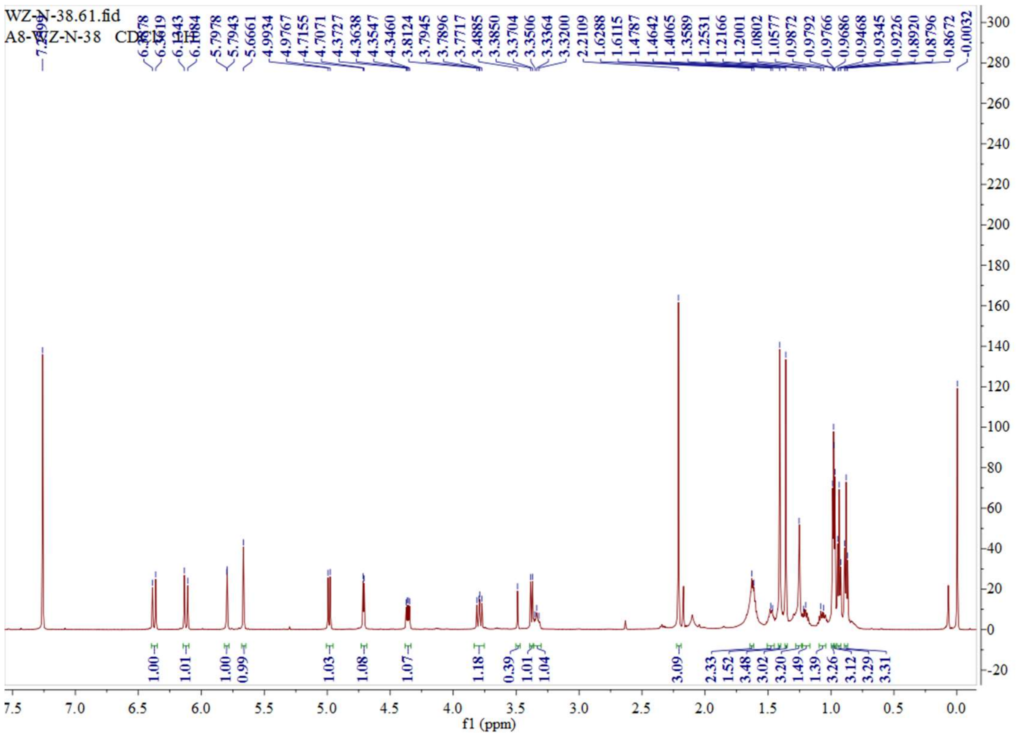

Figure S50. ^{13}C NMR (125 MHz, $CDCl_3$) spectrum of compound **4**

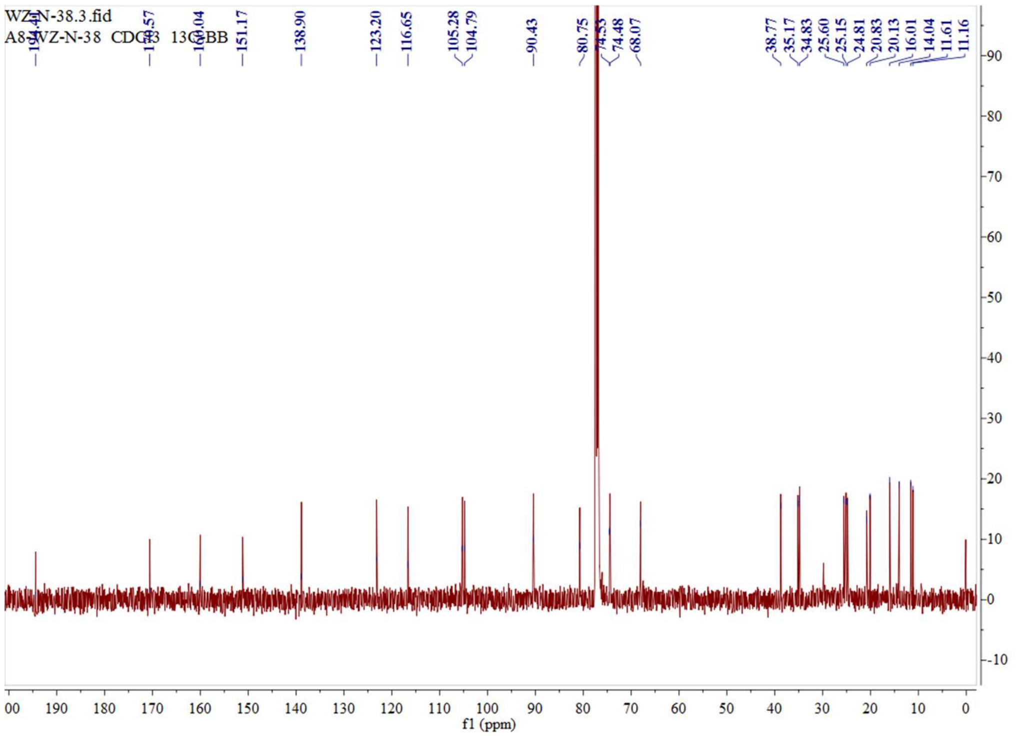

Figure S51. DEPT spectrum of compound **4**

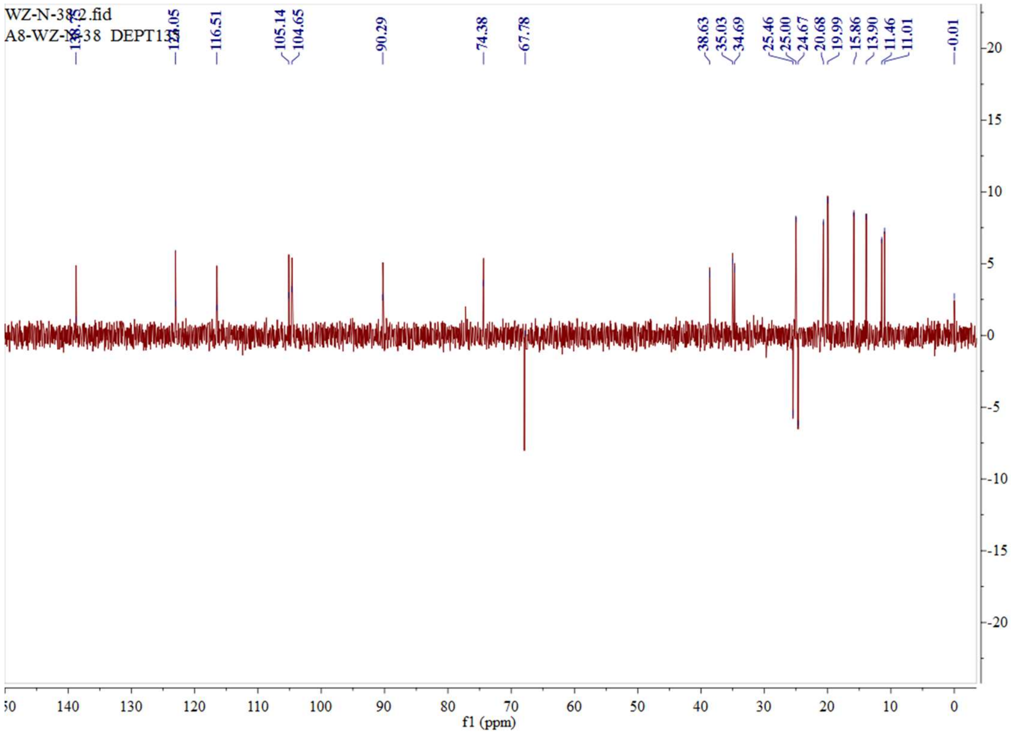

Figure S52. HSQC spectrum of compound **4**

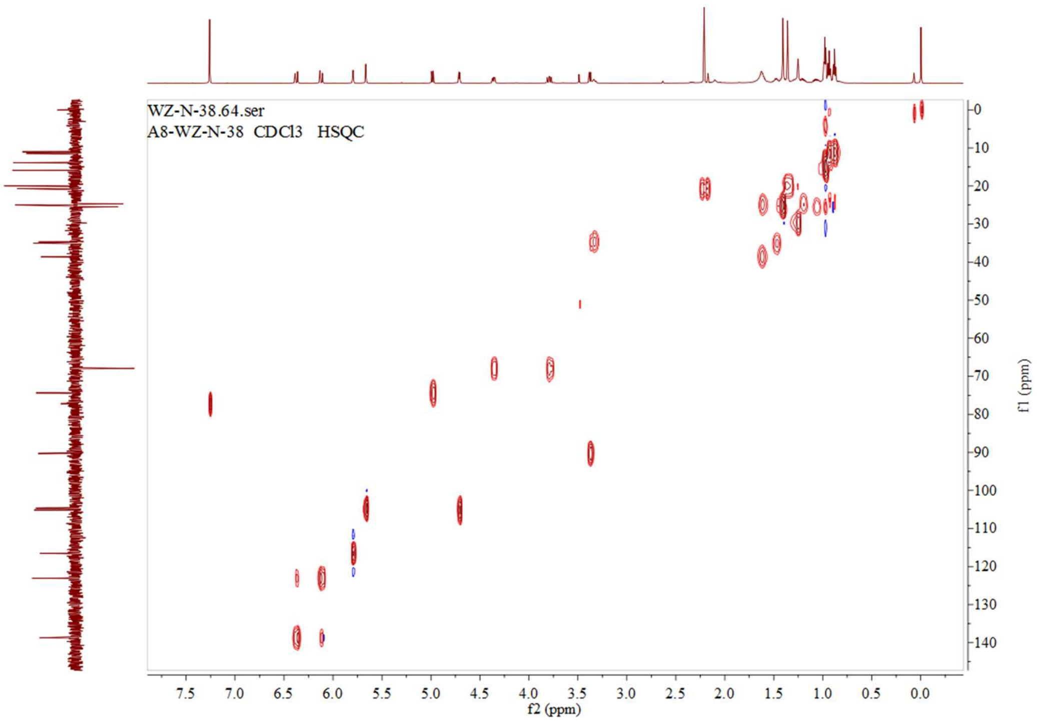

Figure S53. HMBC spectrum of compound **4**

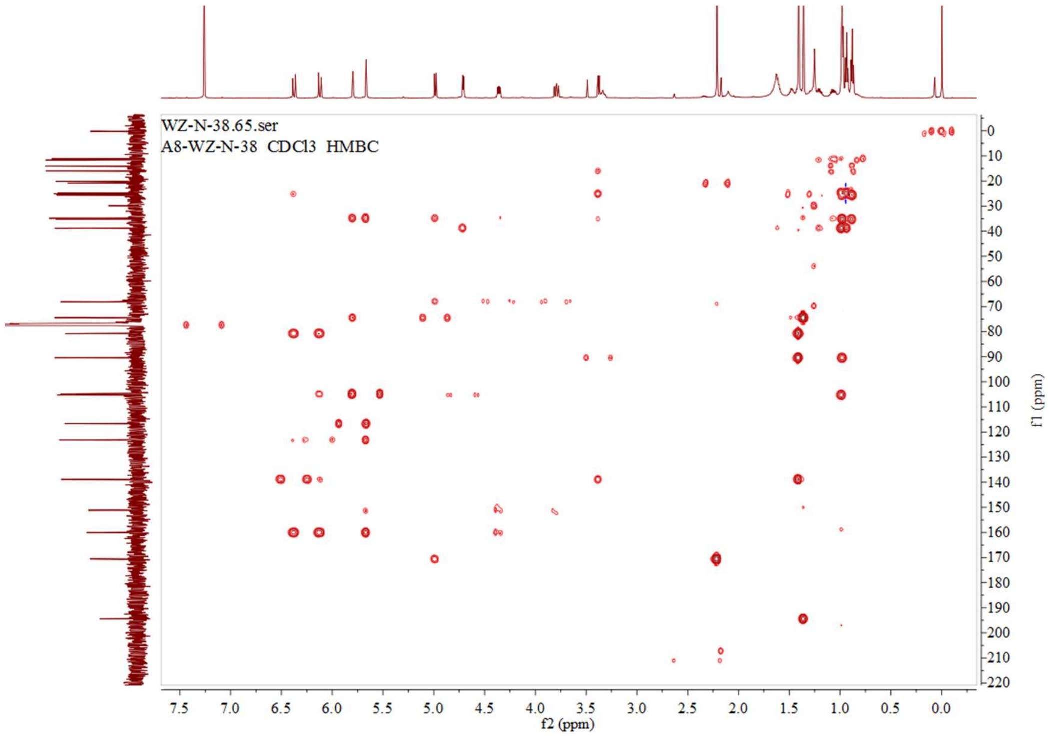

Figure S54. ^{1}H-^{1}H COSY spectrum of compound **4**

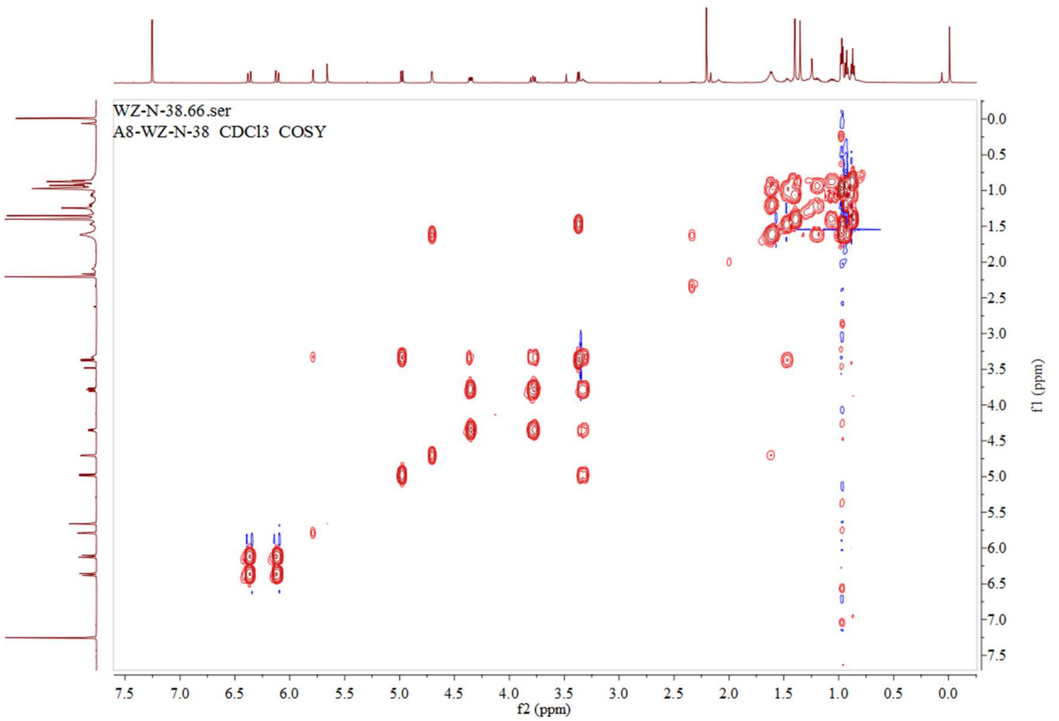

Figure S55. NOESY spectrum of compound **4**

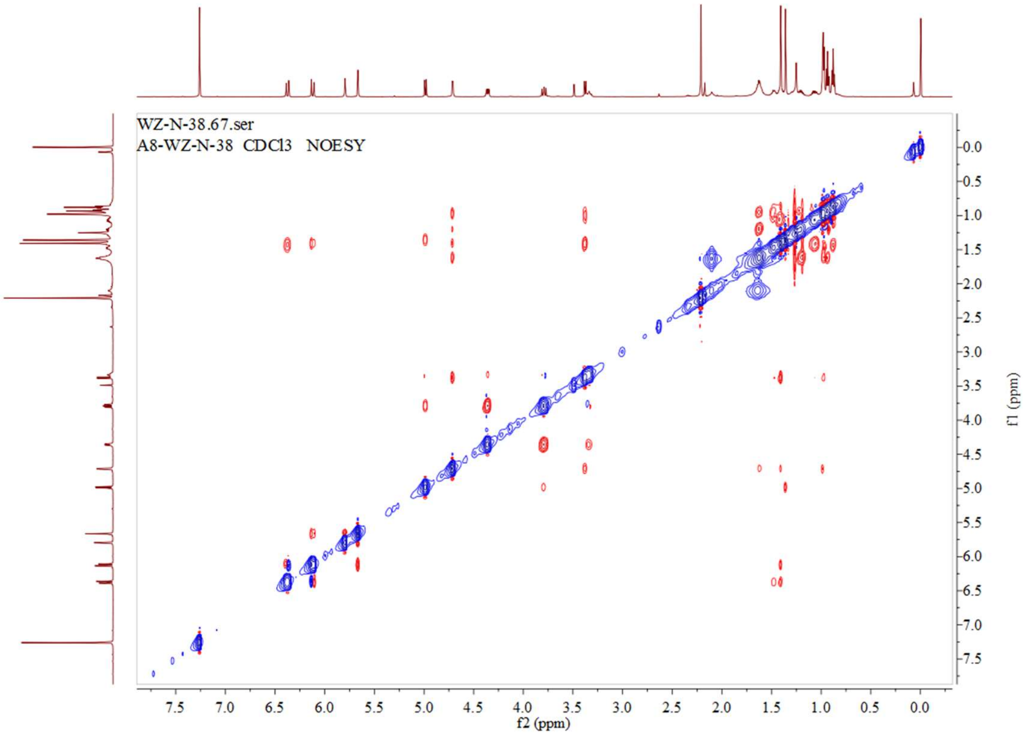

Figure S56. HRESIMS spectrum of compound **4**

Qualitative Analysis Report

**Data Filename**	ESIH202104665.d	**Sample Name**	A8-A8-WZ-N-38
**Sample ID**		**Position**	P1-D3
**Instrument Name**	Agilent G6520 Q-TOF	**Acq Method**	20160322_MS_ESIH_POS_1min.m
**Acquired Time**	10/20/2021 20:34:10	**IRM Calibration Status**	Success
**DA Method**	small molecular data analysis method.m	**Comment**	ESIH by zhuzhenyun

User Spectra

Fragmentor Voltage	Collision Energy	Ionization Mode
175	0	ESI

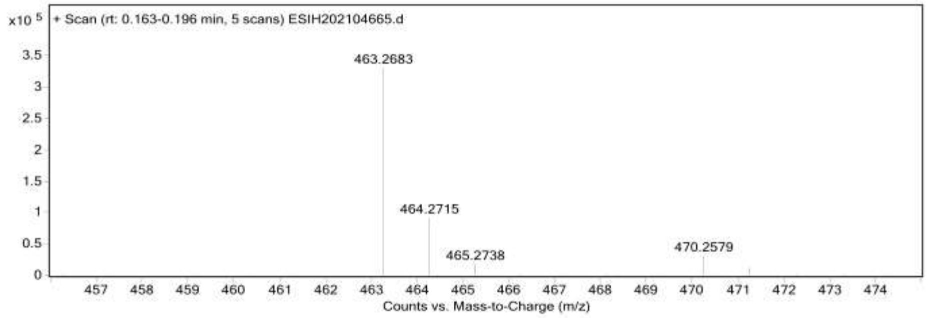

Formula Calculator Results

m/z	Calc m/z	Diff (mDa)	Diff (ppm)	Ion Formula	Ion
463.2683	463.269	0.68	1.47	C26 H39 O7	(M+H)+

--- End Of Report ---

Figure S57. UV spectrum of compound **4**

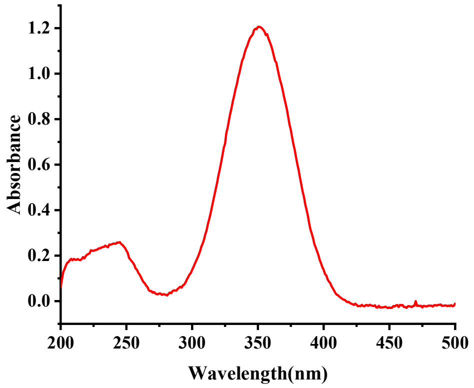

Figure S58. IR spectrum of compound **4**

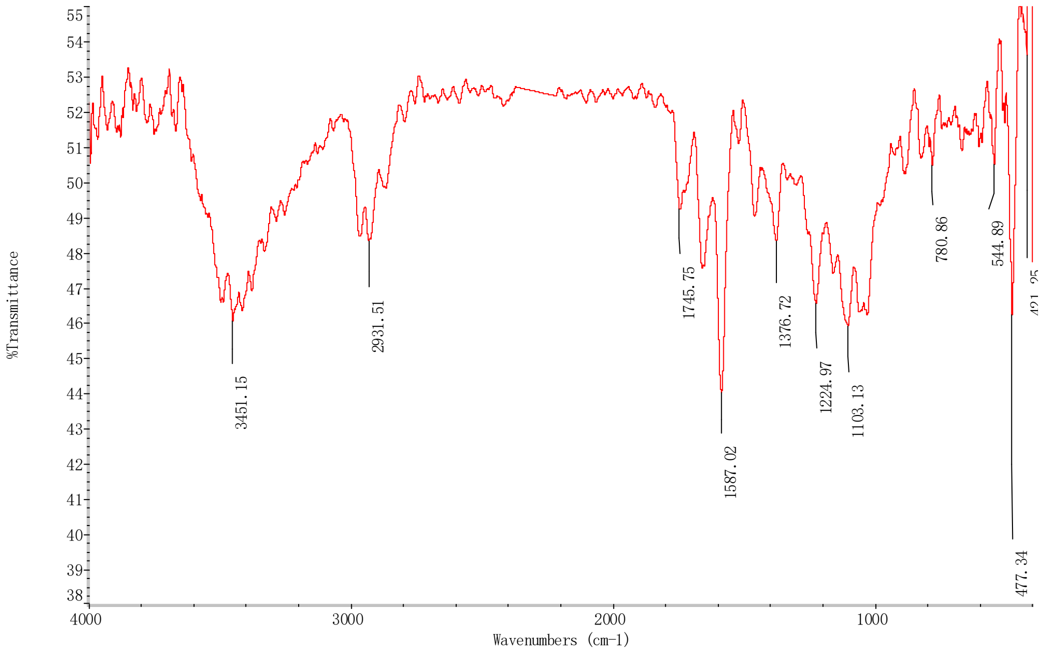

Figure S59. ^{1}H NMR (600 MHz, $CDCl_3$) spectrum of compound **5**

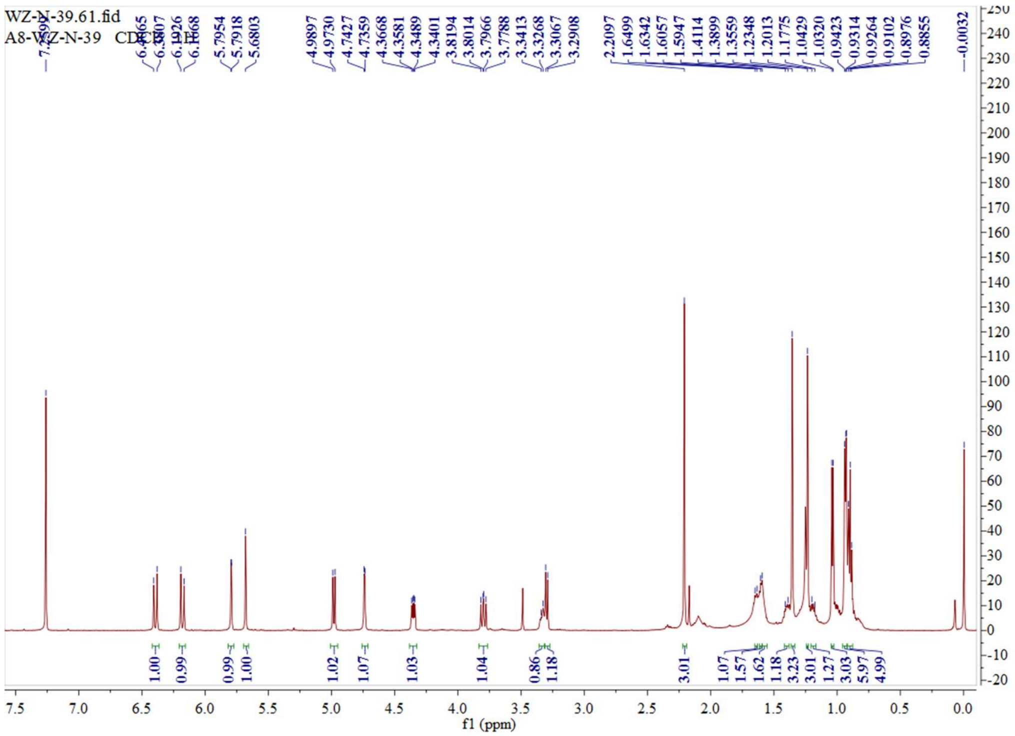

Figure S60. ^{13}C NMR (125 MHz, $CDCl_3$) spectrum of compound **5**

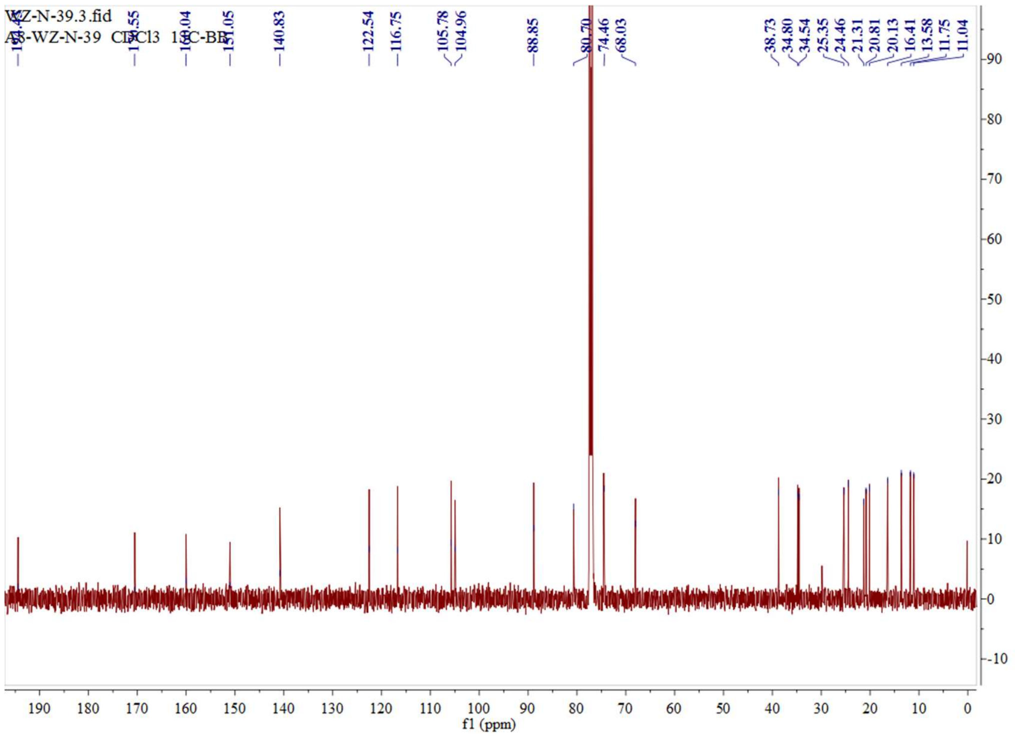

Figure S61. DEPT spectrum of compound **5**

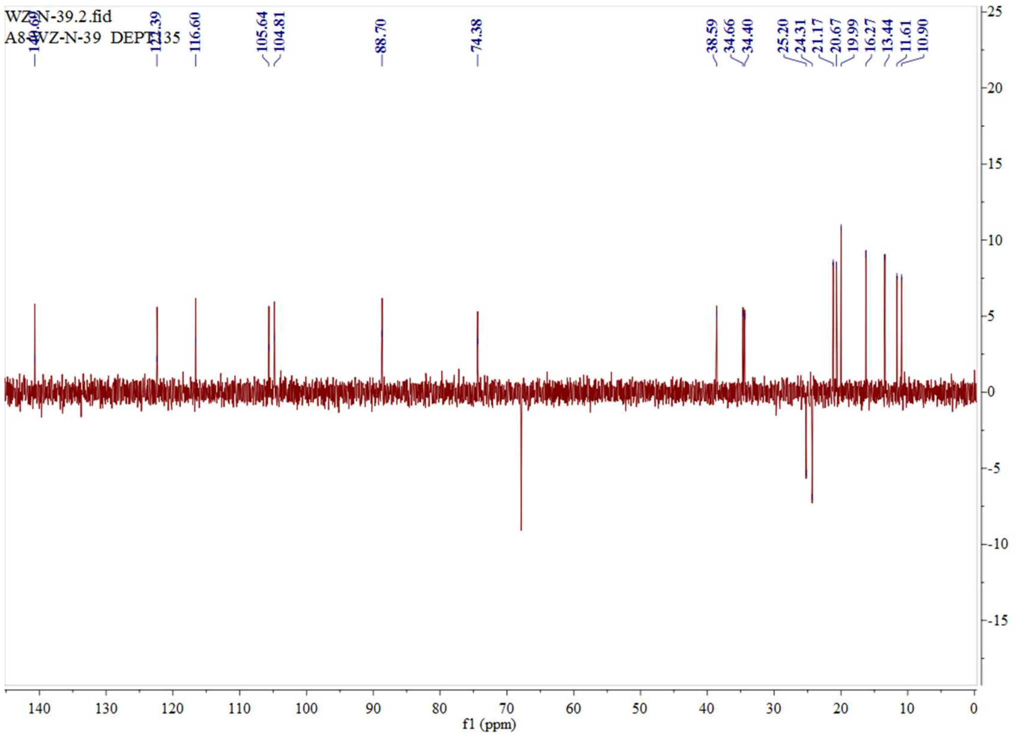

Figure S62. HSQC spectrum of compound **5**

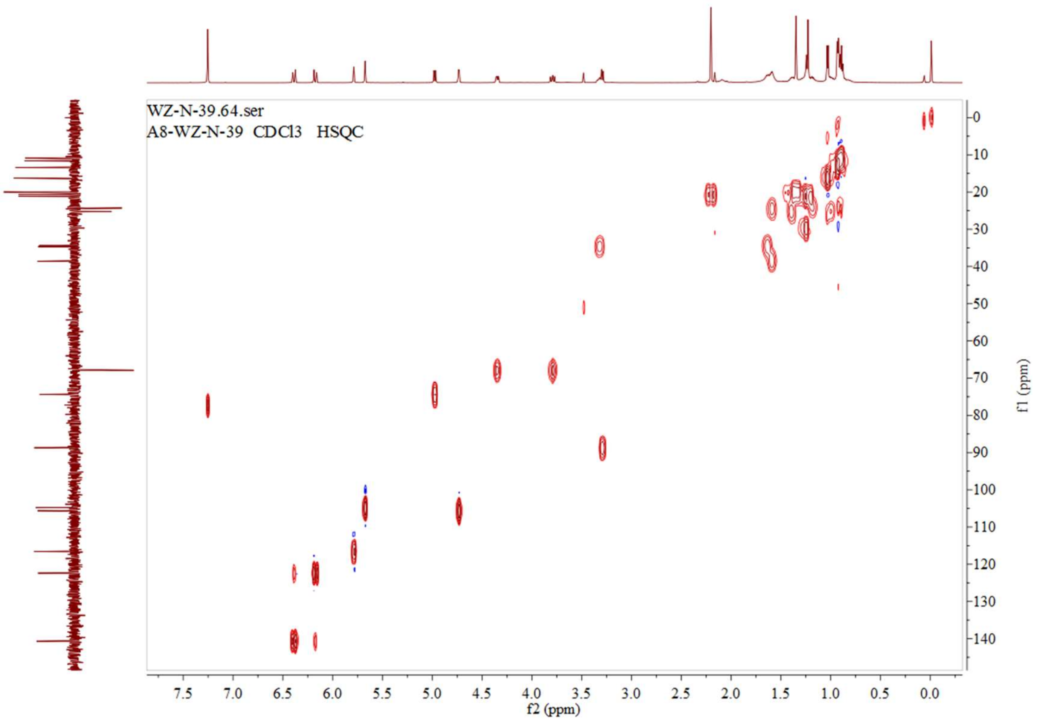

Figure S63. HMBC spectrum of compound **5**

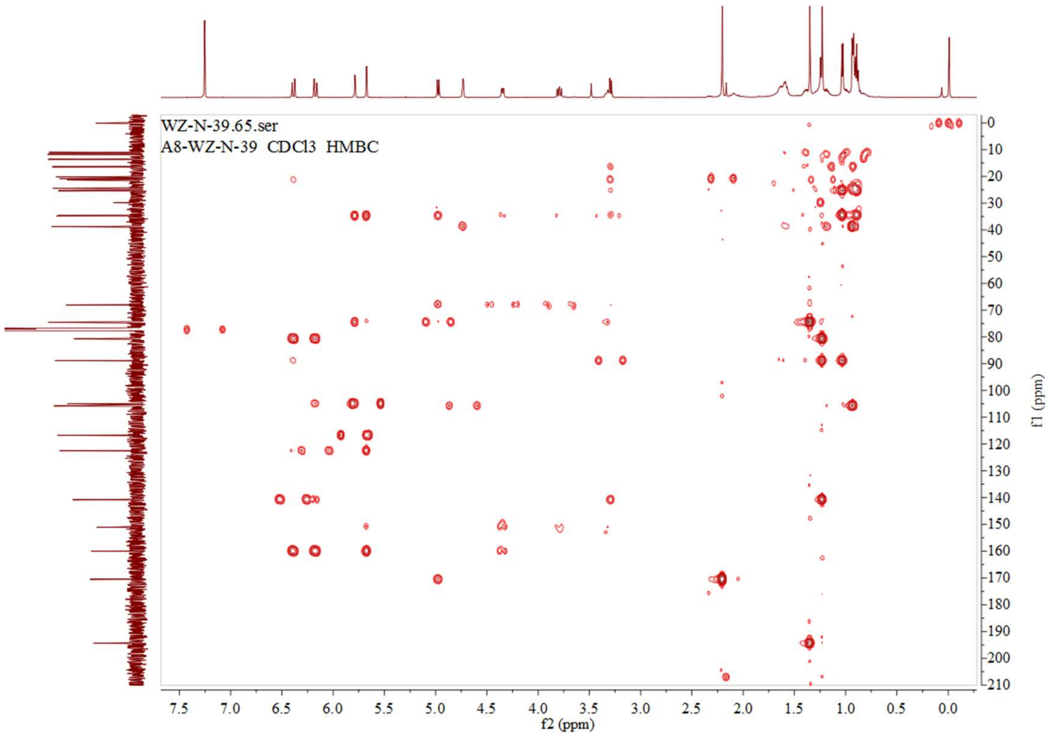

Figure S64. ^{1}H-^{1}H COSY spectrum of compound **5**

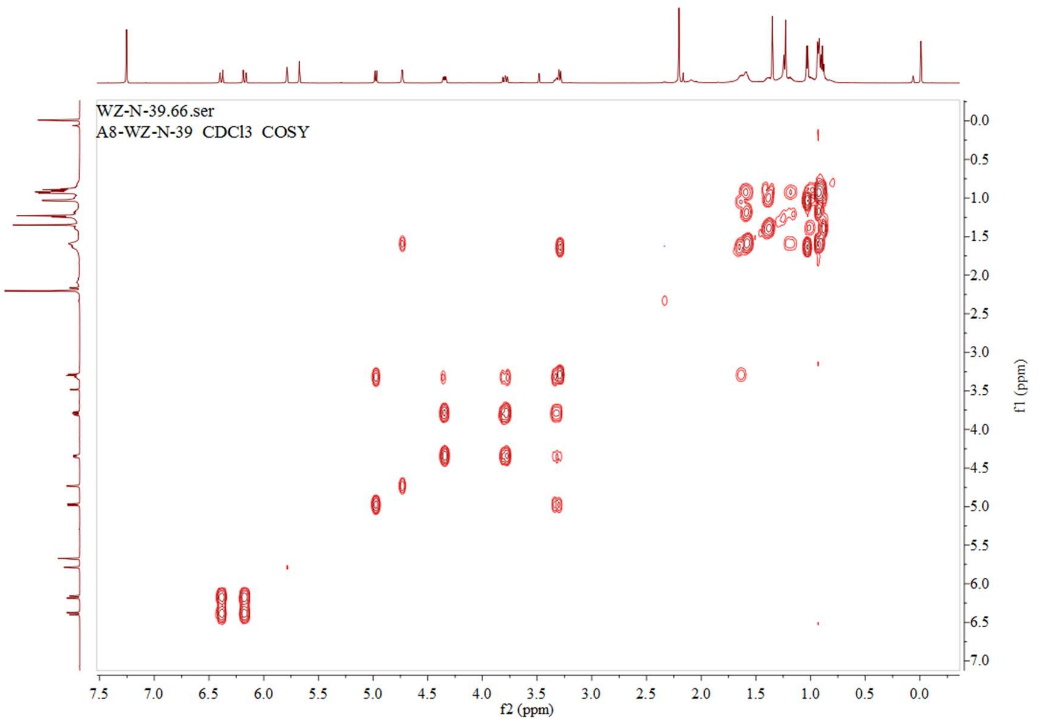

Figure S65. NOESY spectrum of compound **5**

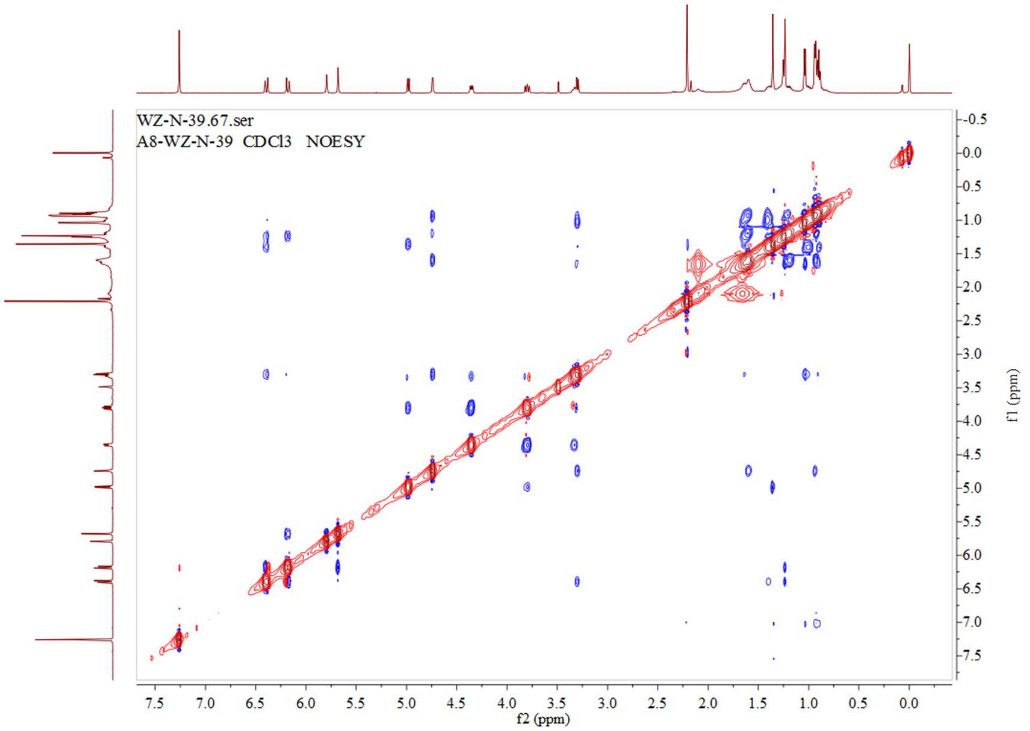

Figure S66. HRESIMS spectrum of compound **5**

Qualitative Analysis Report

**Data Filename**	ESIH202104666.d	**Sample Name**	A8-A8-WZ-N-39
**Sample ID**		**Position**	P1-D4
**Instrument Name**	Agilent G6520 Q-TOF	**Acq Method**	20160322_MS_ESIH_POS_1min.m
**Acquired Time**	10/20/2021 20:35:27	**IRM Calibration Status**	Success
**DA Method**	small molecular data analysis method.m	**Comment**	ESIH by zhuzhenyun

User Spectra

Fragmentor Voltage	Collision Energy	Ionization Mode
175	0	ESI

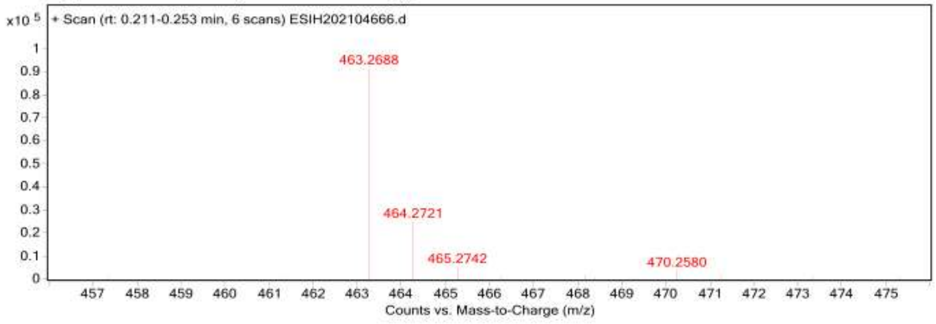

Formula Calculator Results

m/z	Calc m/z	Diff (mDa)	Diff (ppm)	Ion Formula	Ion
463.2688	463.269	0.19	0.41	C26 H39 O7	(M+H)+

--- End Of Report ---

Figure S67. UV spectrum of compound **5**

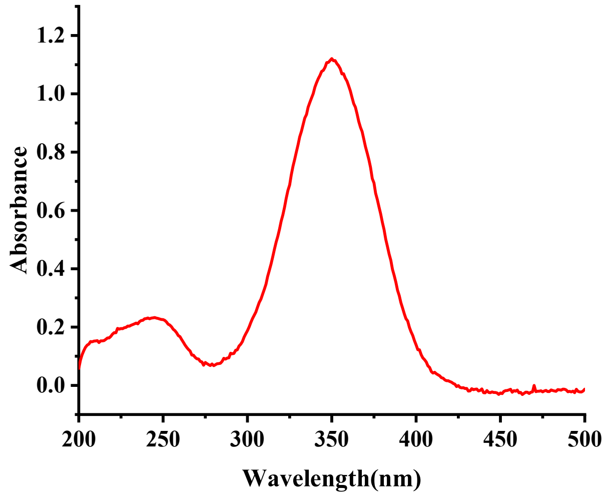

Figure S68. IR spectrum of compound **5**

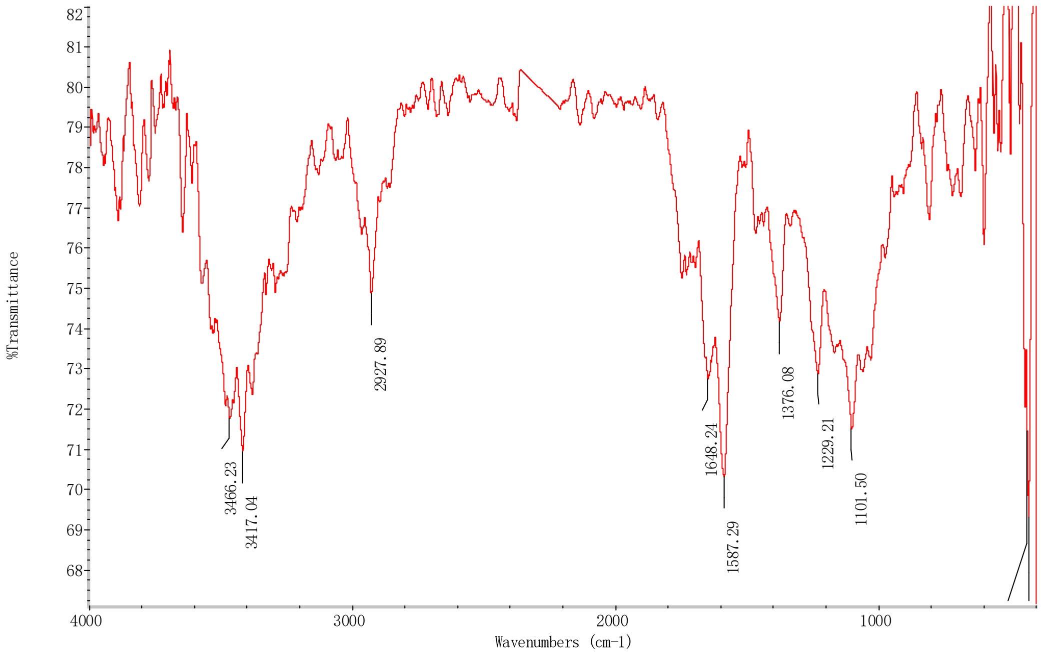

Figure S69. ^{1}H NMR (600 MHz, $CDCl_3$) spectrum of compound **6**

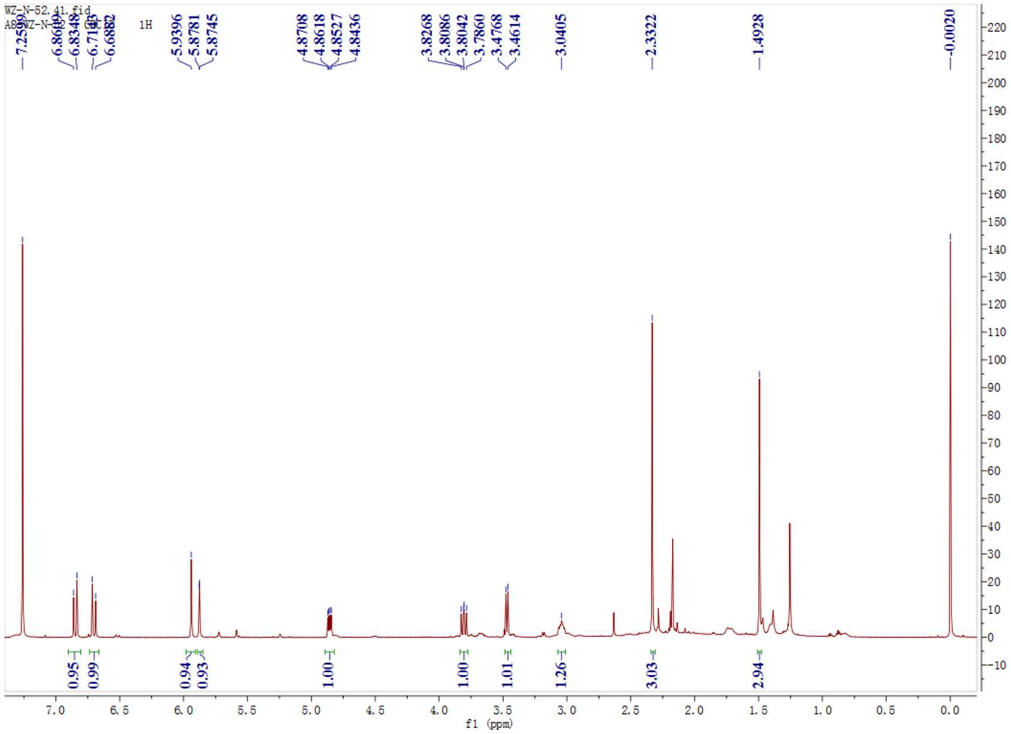

Figure S70. ^{13}C NMR (125 MHz, $CDCl_3$) spectrum of compound **6**

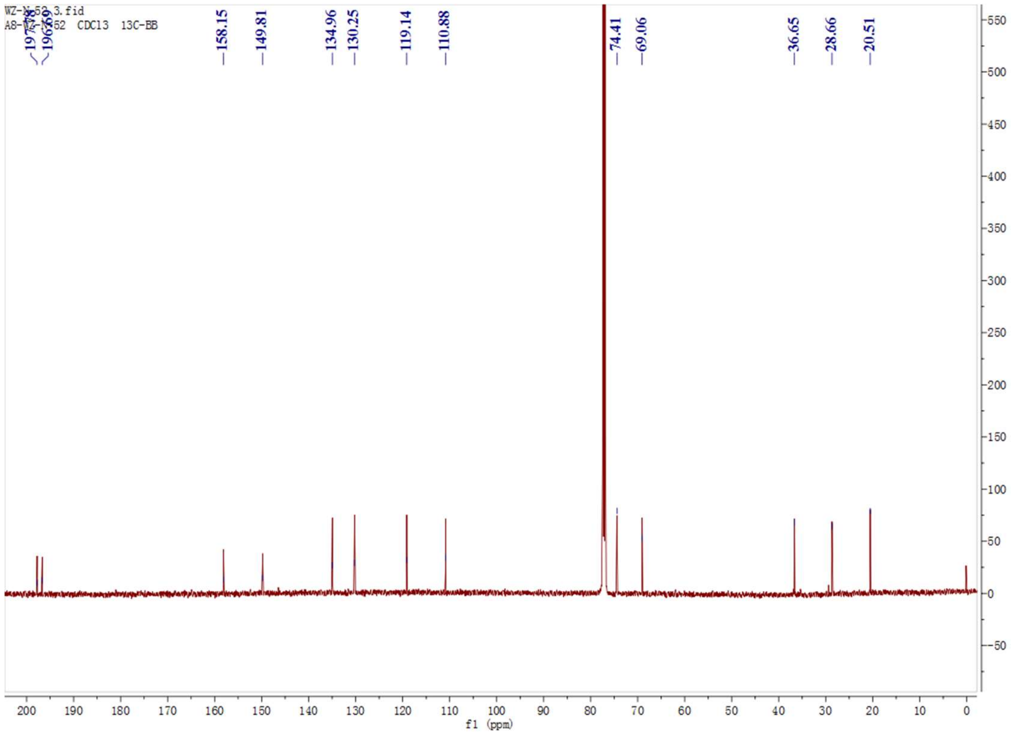

Figure S71. DEPT spectrum of compound **6**

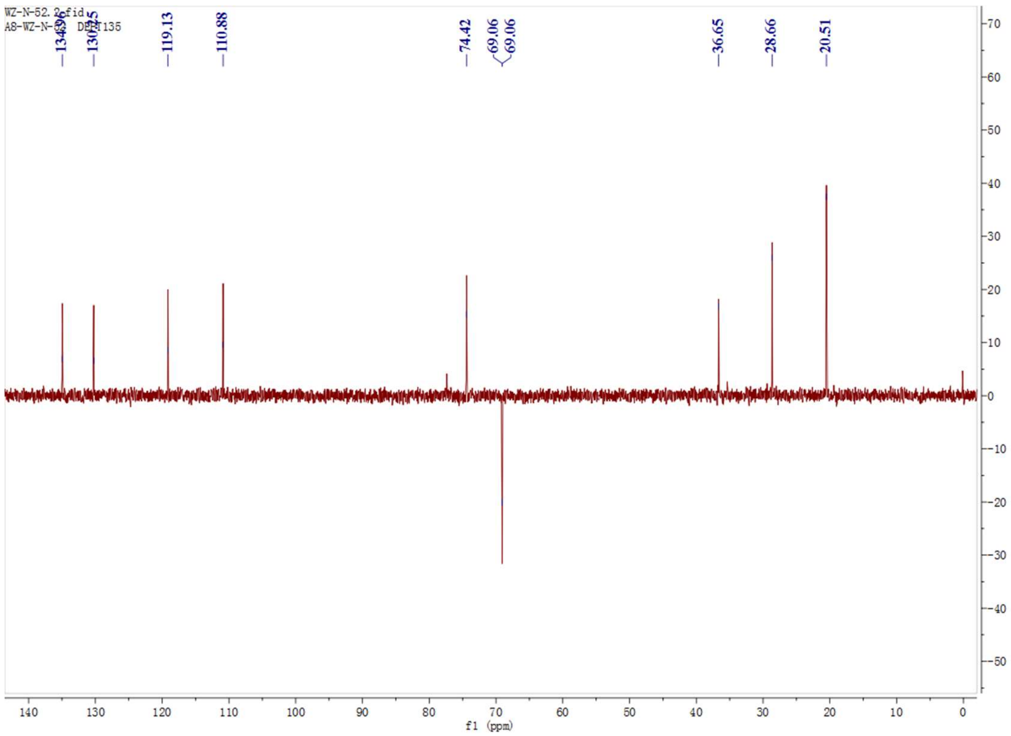

Figure S72. HSQC spectrum of compound **6**

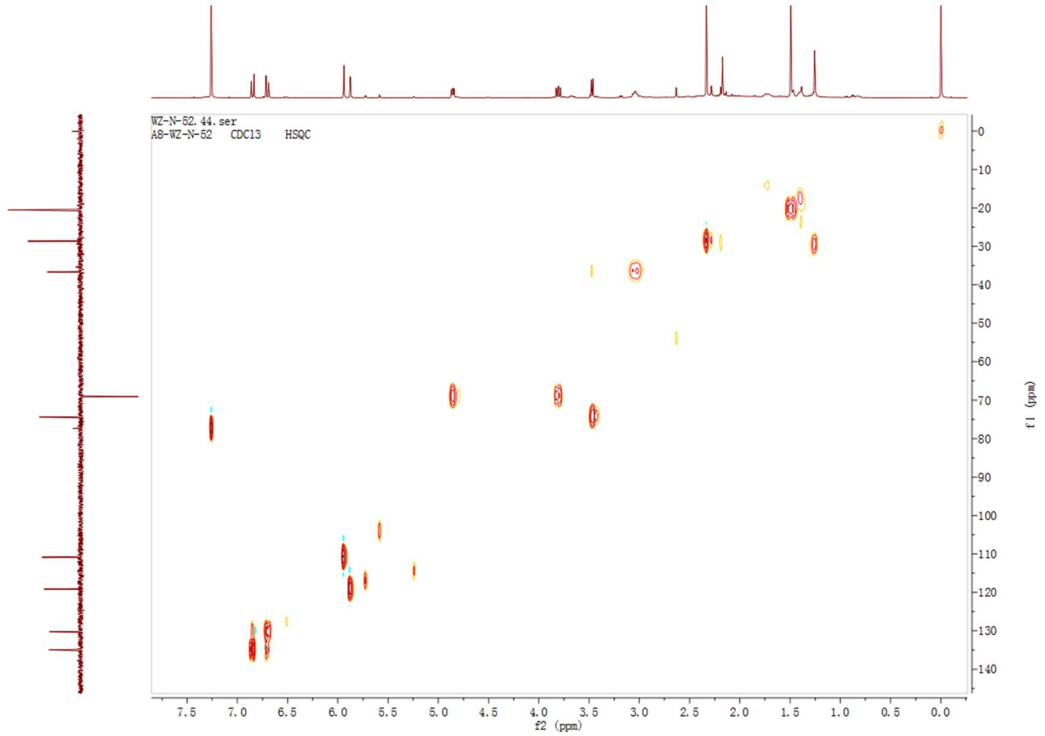

Figure S73. HMBC spectrum of compound **6**

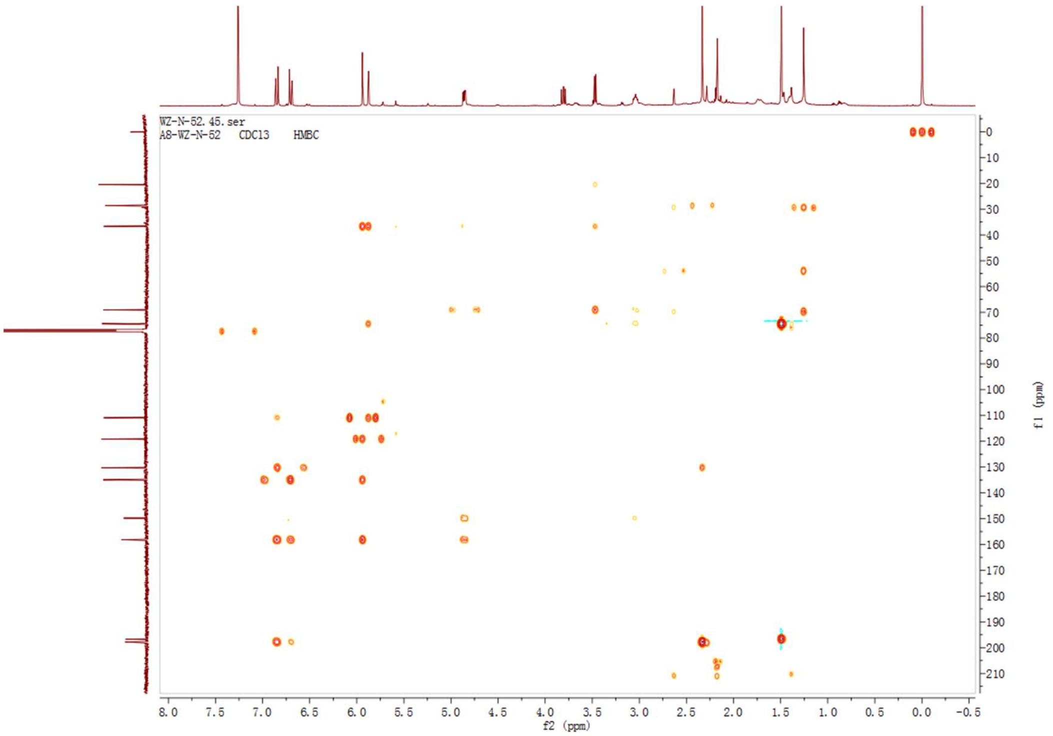

Figure S74. ^{1}H-^{1}H COSY spectrum of compound **6**

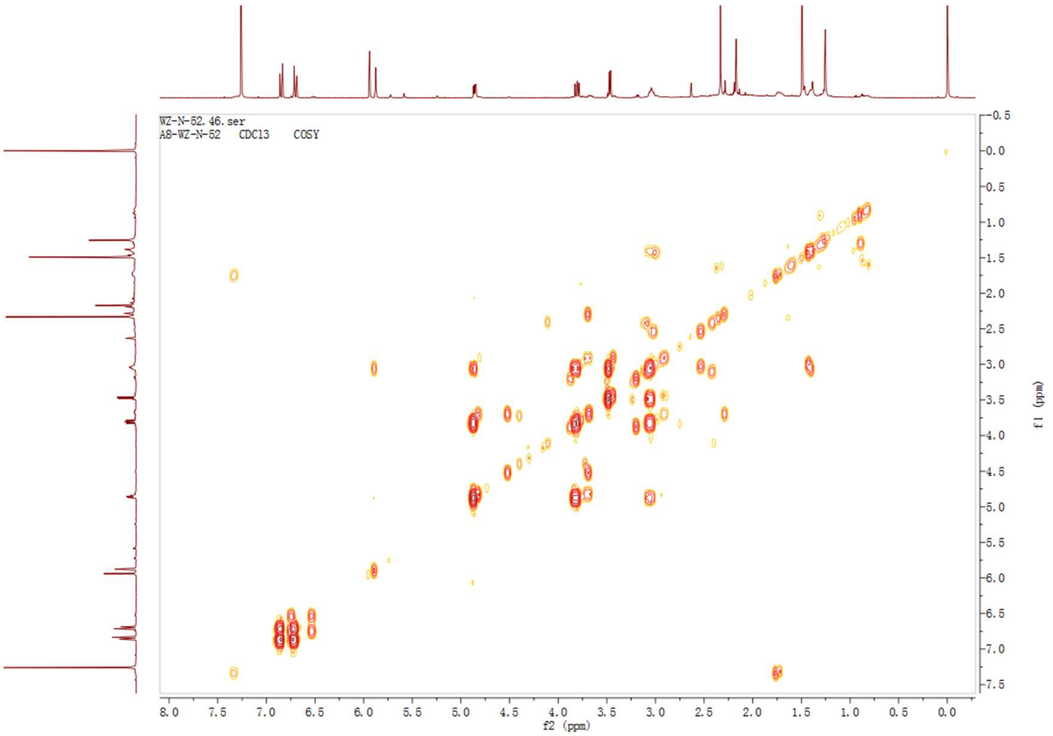

Figure S75. NOESY spectrum of compound **6**

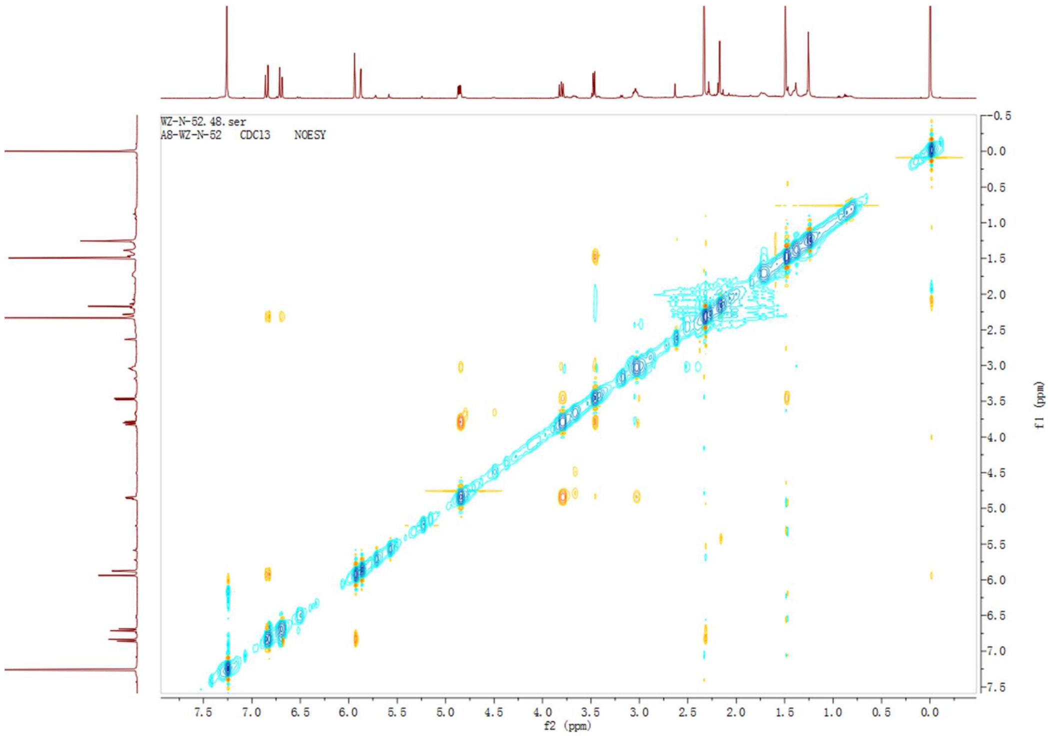

Figure S76. HRESIMS spectrum of compound **6**

Qualitative Analysis Report

**Data Filename**	ESIH202105122.d	**Sample Name**	A8-WZ-N-52
**Sample ID**		**Position**	P1-A2
**Instrument Name**	Agilent G6520 Q-TOF	**Acq Method**	20160322_MS_ESIH_POS_1min.m
**Acquired Time**	11/24/2021 13:55:43	**IRM Calibration Status**	Success
**DA Method**	small molecular data analysis method.m	**Comment**	ESIH by fangsu

User Spectra

**Fragmentor Voltage**	**Collision Energy**	**Ionization Mode**
175	0	ESI

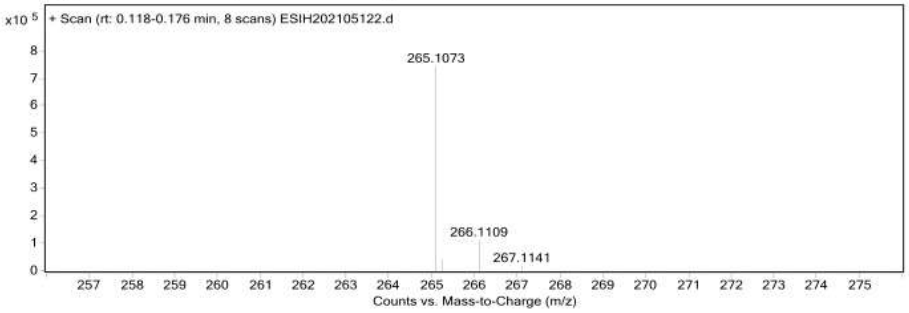

Formula Calculator Results

m/z	Calc m/z	Diff (mDa)	Diff (ppm)	Ion Formula	Ion
265.1073	265.1071	-0.25	-0.93	C14 H17 O5	(M+H)+

--- End Of Report ---

Figure S77. UV spectrum of compound **6**

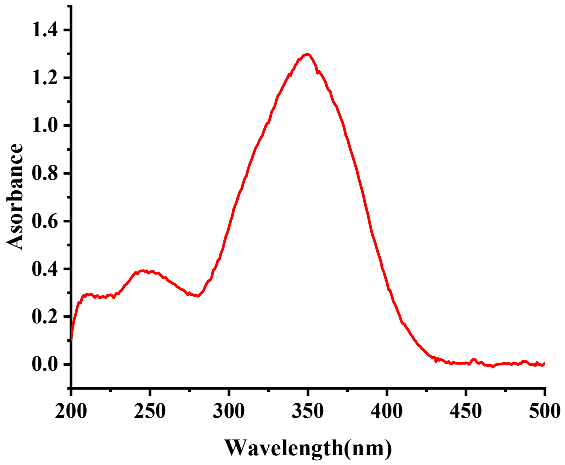

Figure S78. IR spectrum of compound **6**

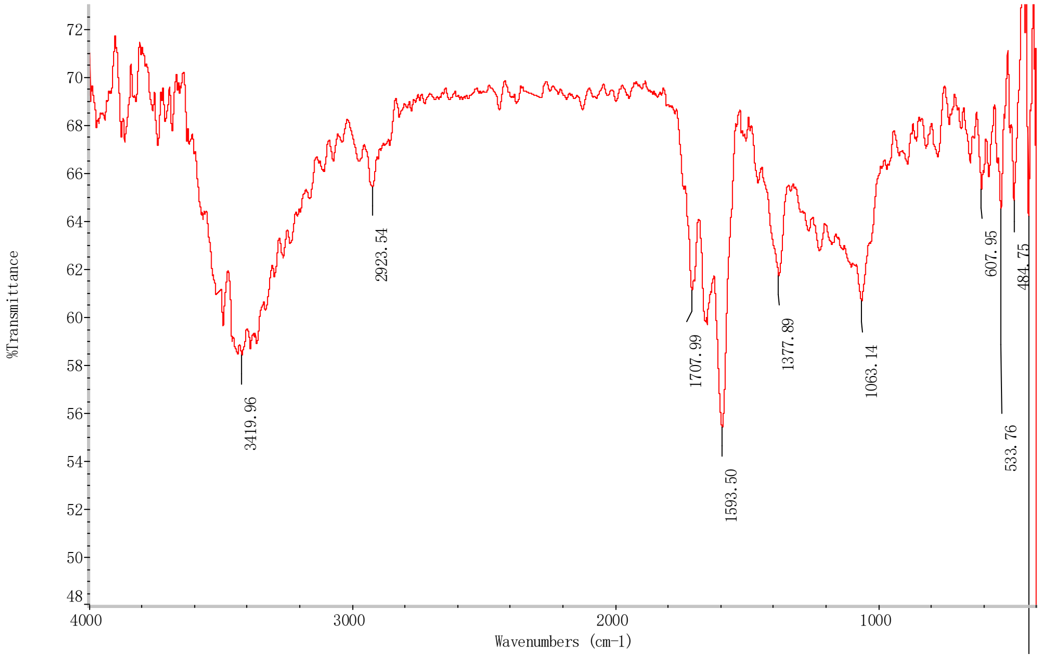

Figure S79. ^{1}H NMR (600 MHz, $CDCl_3$) spectrum of compound **7**

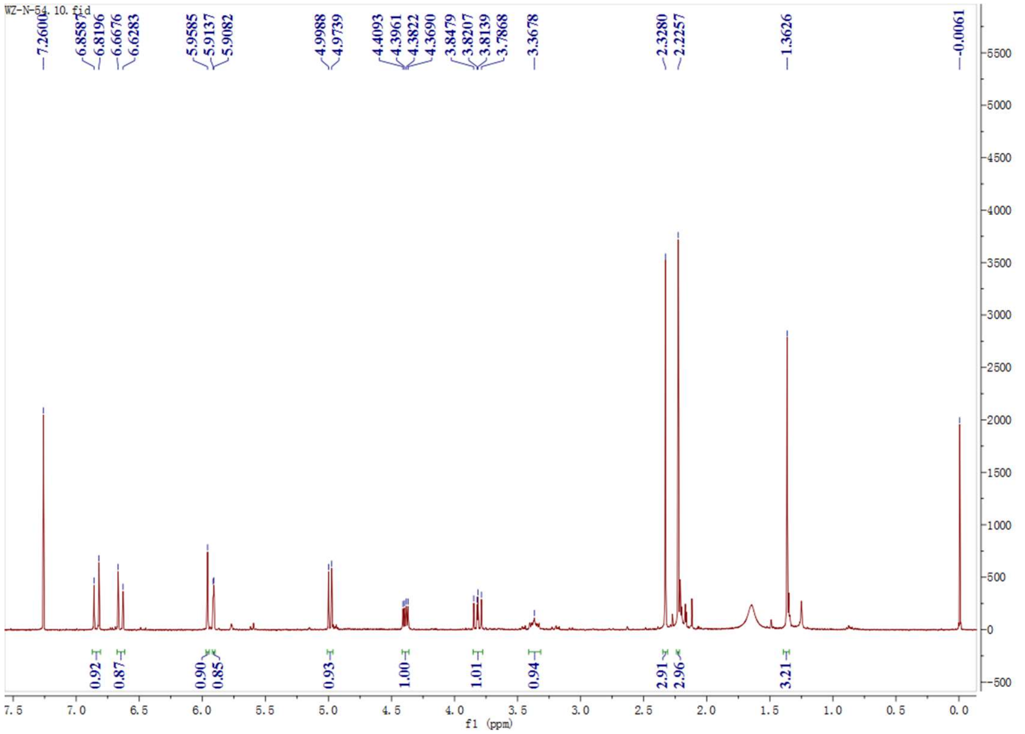

Figure S80. ^{13}C NMR (125 MHz, $CDCl_3$) spectrum of compound **7**

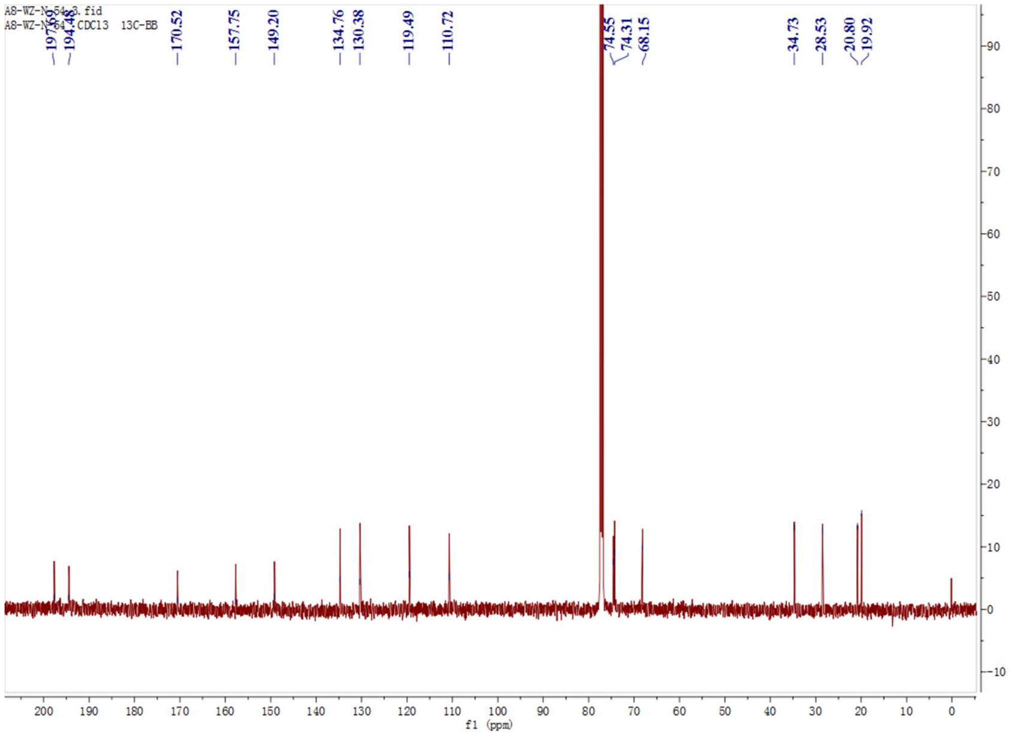

Figure S81. DEPT spectrum of compound **7**

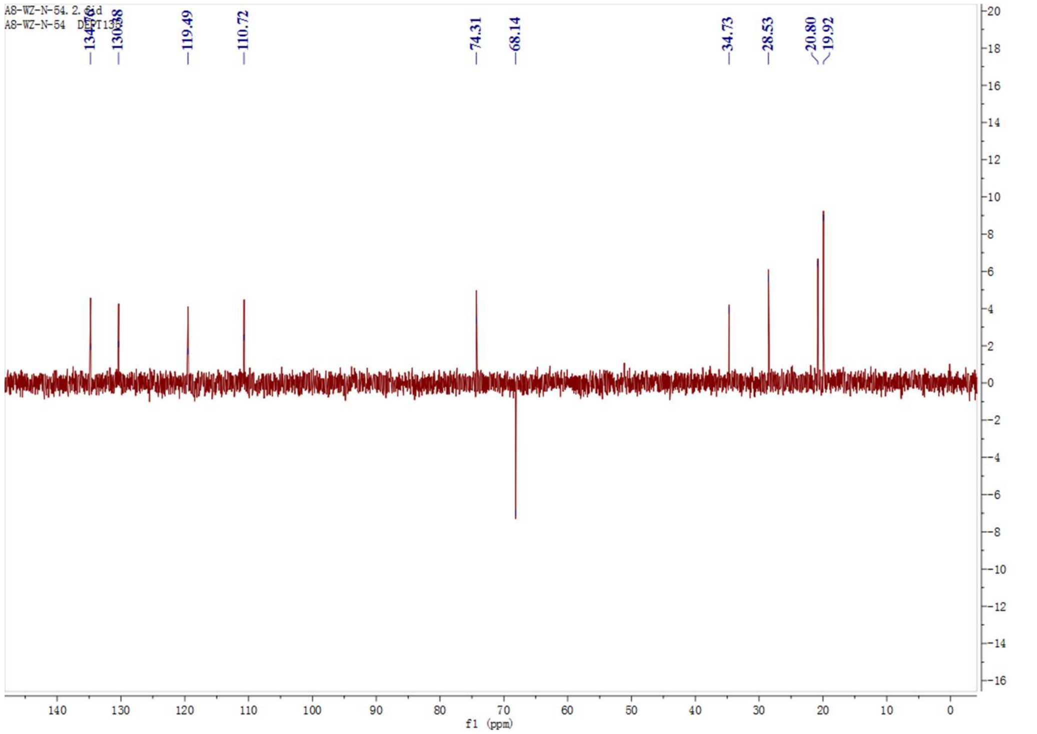

Figure S82. HSQC spectrum of compound **7**

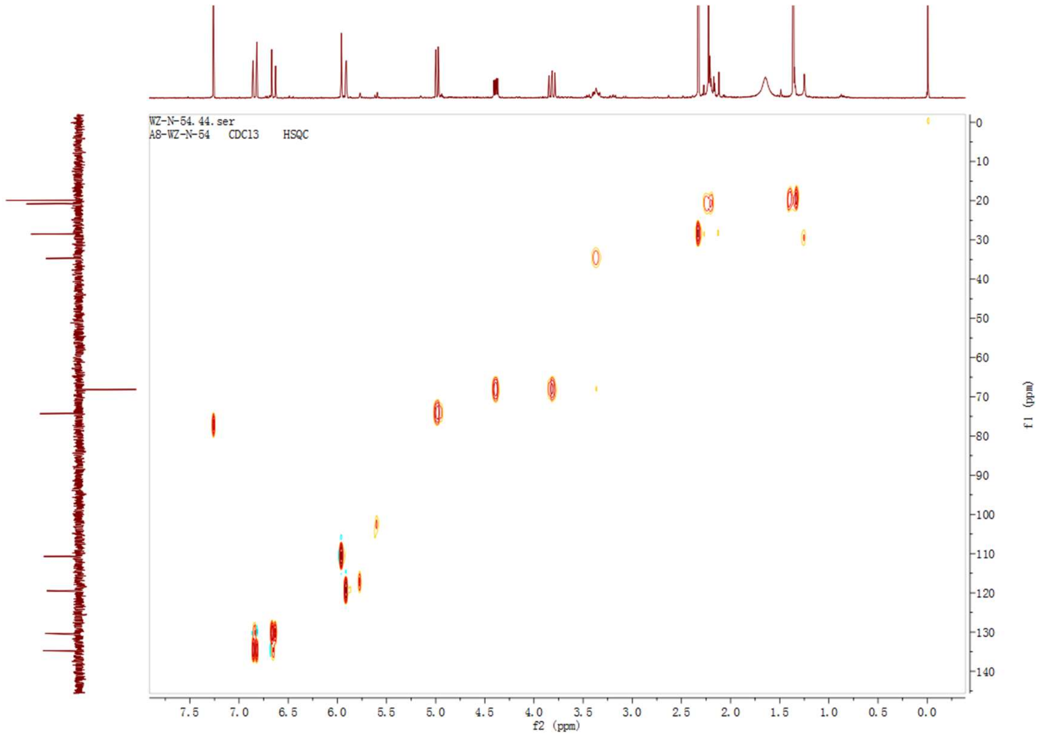

Figure S83. HMBC spectrum of compound **7**

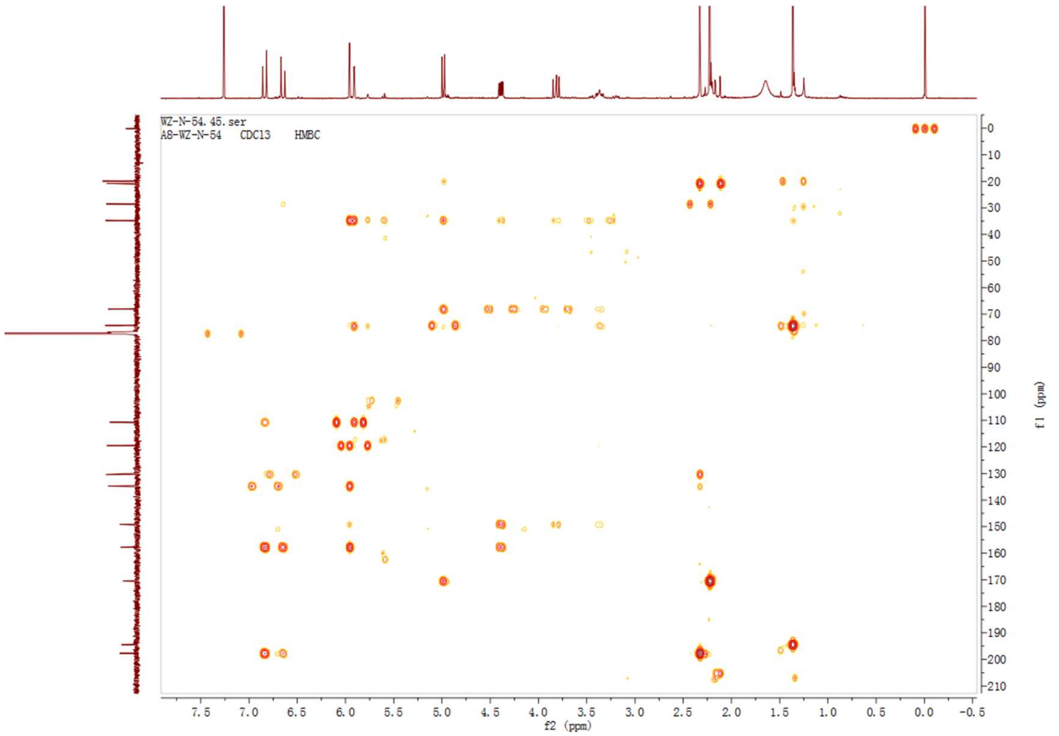

Figure S84. ^{1}H-^{1}H COSY spectrum of compound **7**

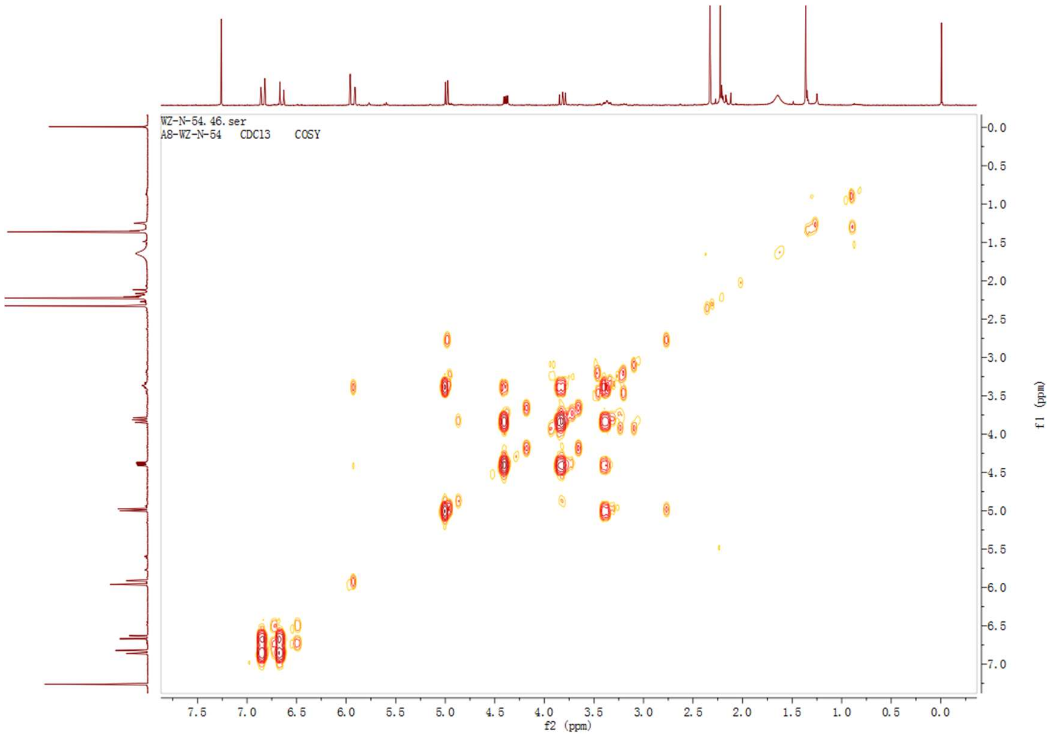

Figure S85. NOESY spectrum of compound **7**

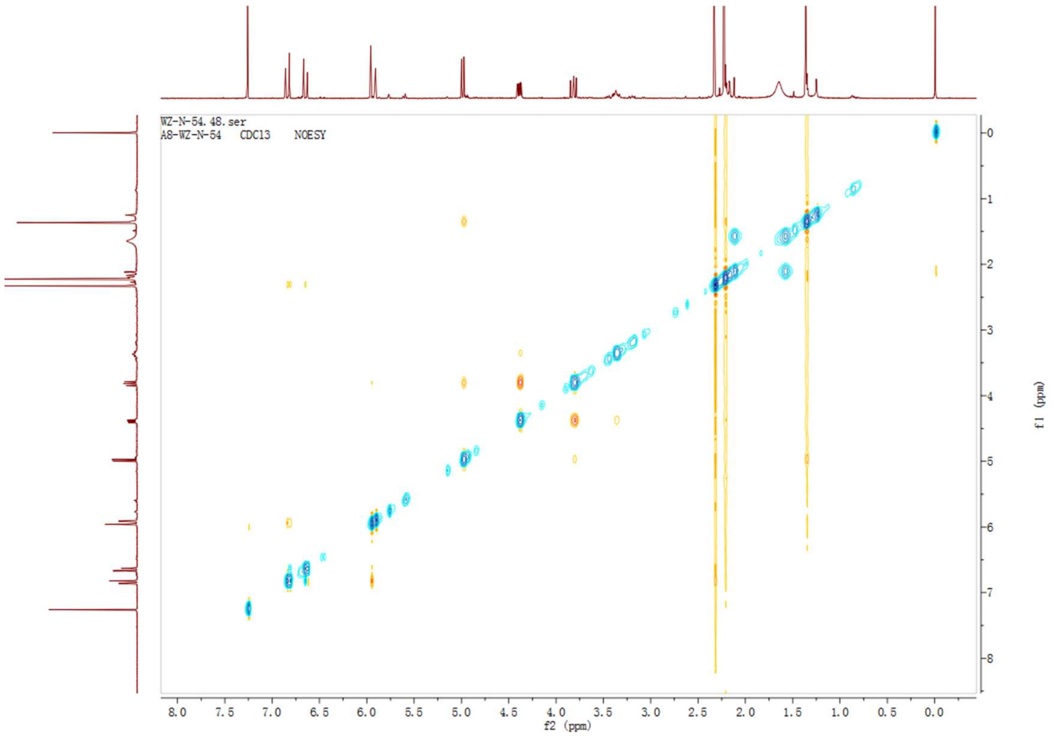

Figure S86. HRESIMS spectrum of compound **7**

Qualitative Analysis Report

**Data Filename**	ESIH202105123.d	**Sample Name**	A8-WZ-N-54
**Sample ID**		**Position**	P1-A3
**Instrument Name**	Agilent G6520 Q-TOF	**Acq Method**	20160322_MS_ESIH_POS_1min.m
**Acquired Time**	11/24/2021 13:57:00	**IRM Calibration Status**	Success
**DA Method**	small molecular data analysis method.m	**Comment**	ESIH by fangsu

User Spectra

Fragmentor Voltage	Collision Energy	Ionization Mode
175	0	ESI

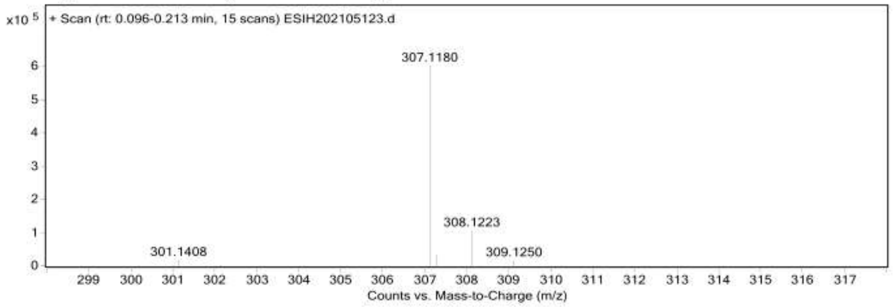

Formula Calculator Results

m/z	Calc m/z	Diff (mDa)	Diff (ppm)	Ion Formula	Ion
307.118	307.1176	-0.36	-1.16	C16 H19 O6	(M+H)+

--- End Of Report ---

Figure S87. UV spectrum of compound **7**

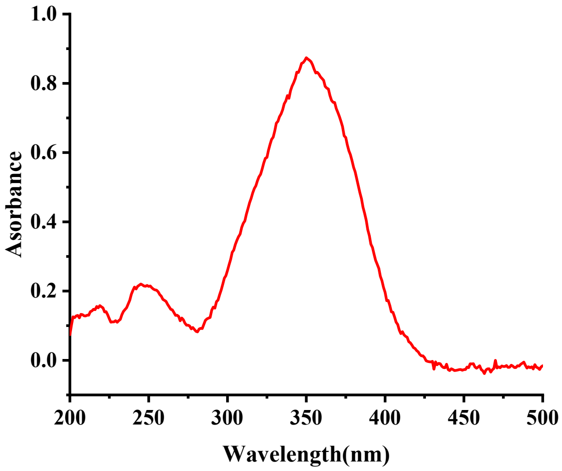

Figure S88. IR spectrum of compound **7**

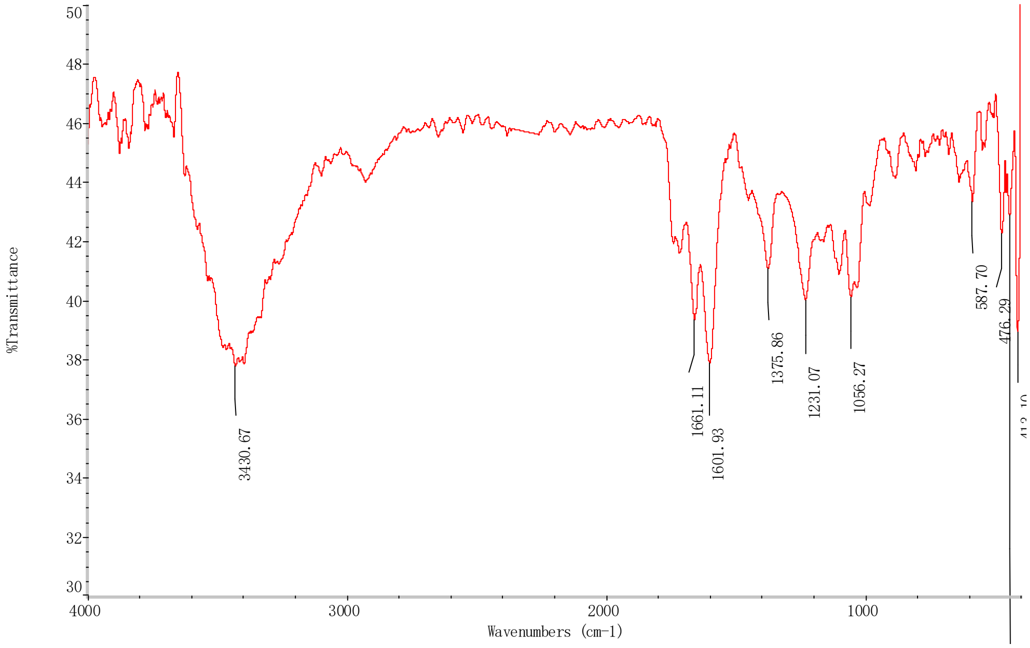

Figure S89. ^{1}H NMR (600 MHz, $CDCl_3$) spectrum of compound **8**

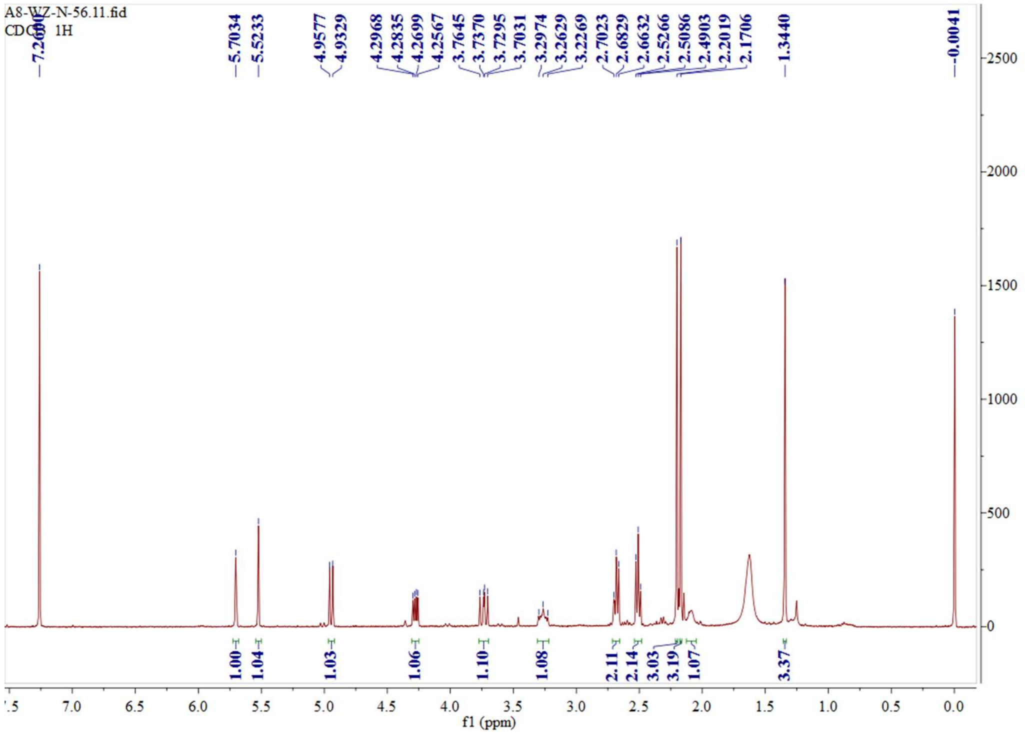

Figure S90. ^{13}C NMR (125 MHz, $CDCl_3$) spectrum of compound **8**

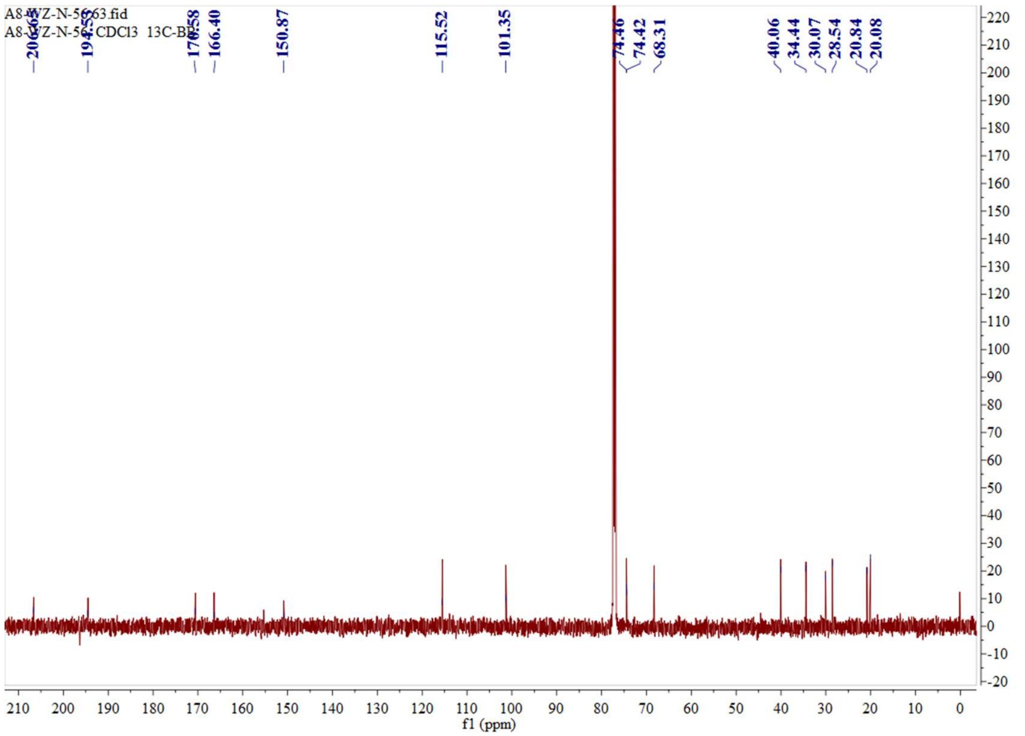

Figure S91. DEPT spectrum of compound **8**

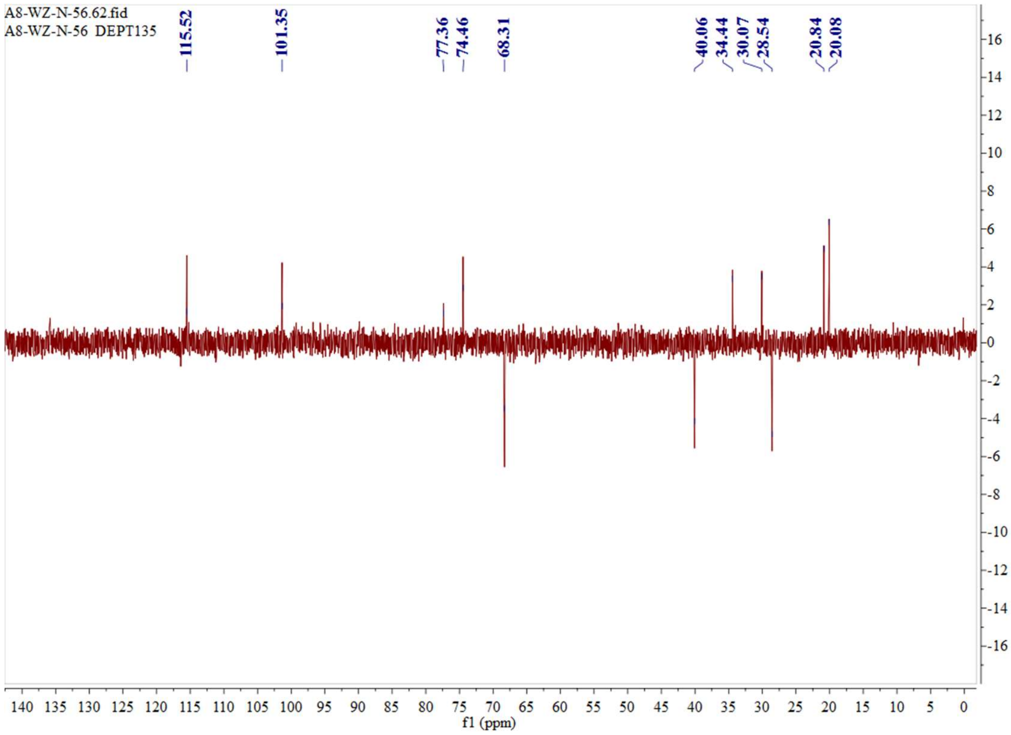

Figure S92. HSQC spectrum of compound **8**

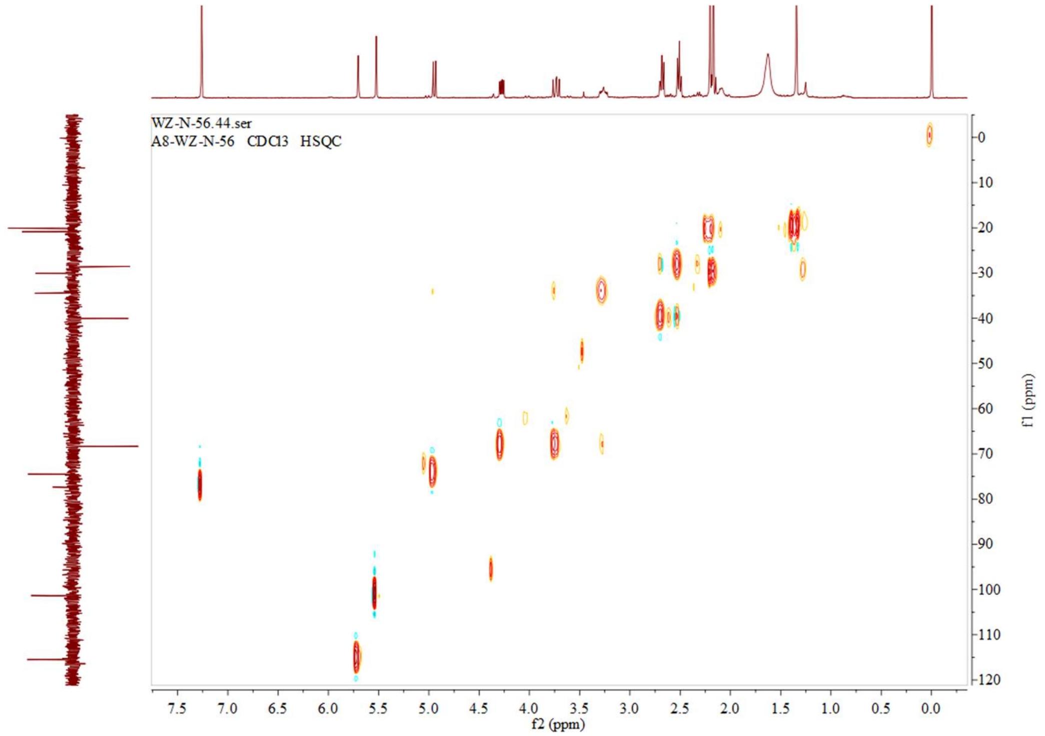

Figure S93. HMBC spectrum of compound **8**

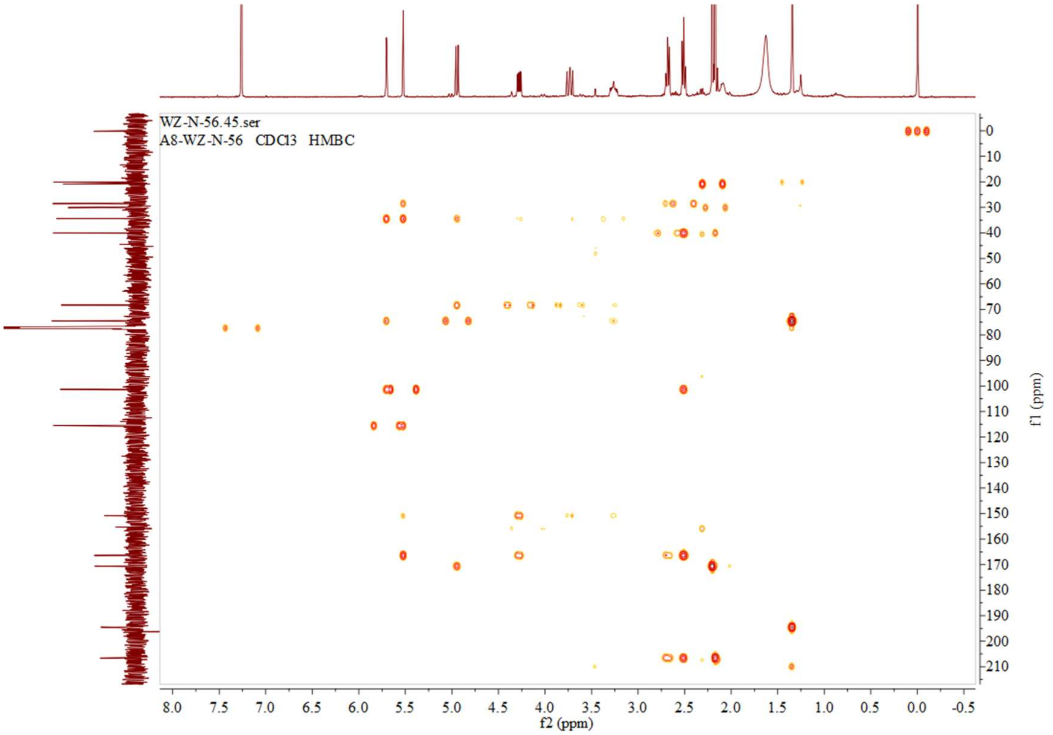

Figure S94. 1H-1H COSY spectrum of compound **8**

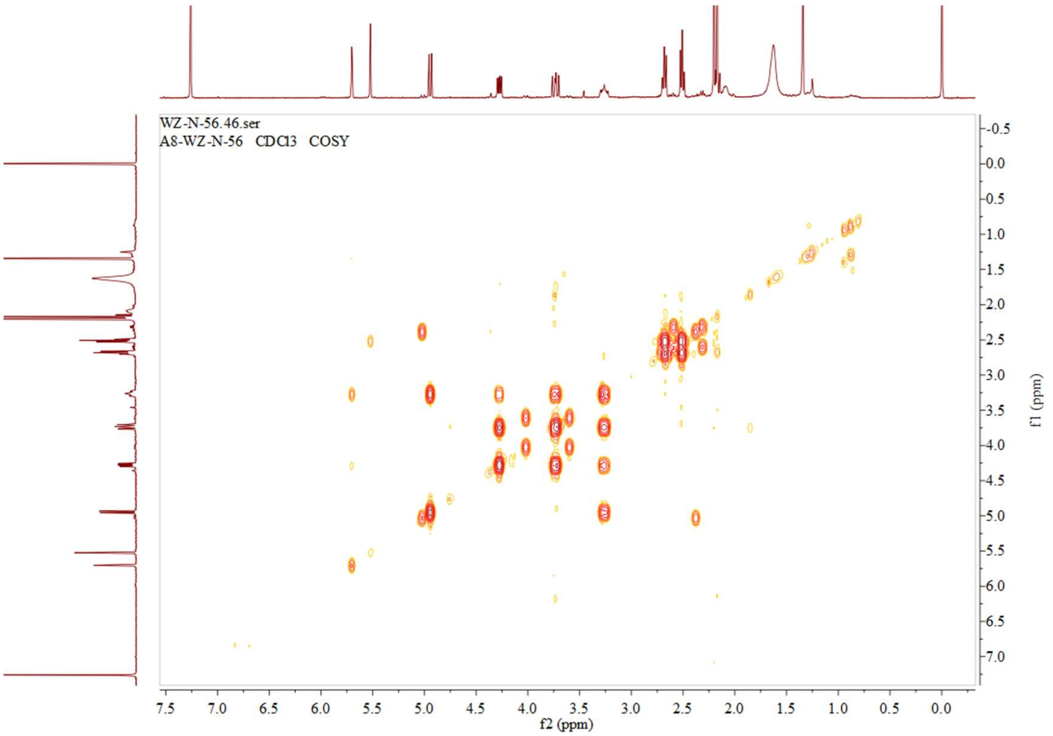

Figure S95. NOESY spectrum of compound **8**

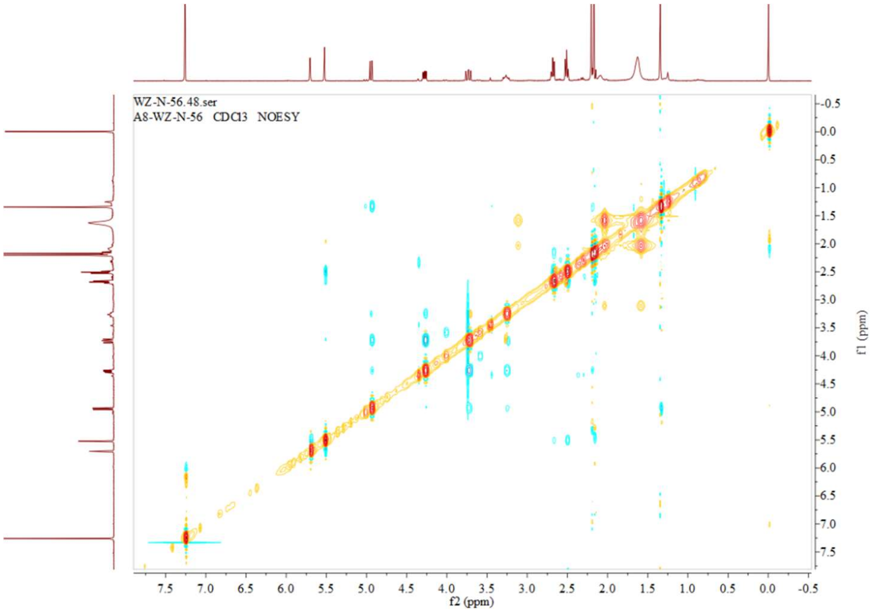

Figure S96. HRESIMS spectrum of compound **8**

Mass Spectrum SmartFormula Report

Analysis Info

		Acquisition Date	2022-05-17 10:32:19
Analysis Name	D:\Data\A501\WYL\2022\20220517\B502-WZ-N-56_GC3_01_4306.d		
Method	lc-ms_as_ms-0.5MIN.m	Operator	Demo User
Sample Name	B502-WZ-N-56	Instrument	compact 8255754.20156
Comment			

Acquisition Parameter

Source Type	ESI	Ion Polarity	Positive	Set Nebulizer	1.8 Bar
Focus	Not active	Set Capillary	3500 V	Set Dry Heater	220 °C
Scan Begin	50 m/z	Set End Plate Offset	-500 V	Set Dry Gas	4.0 l/min
Scan End	2500 m/z	Set Charging Voltage	2000 V	Set Divert Valve	Waste
		Set Corona	0 nA	Set APCI Heater	0 °C

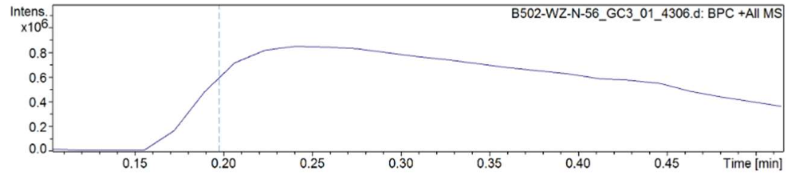

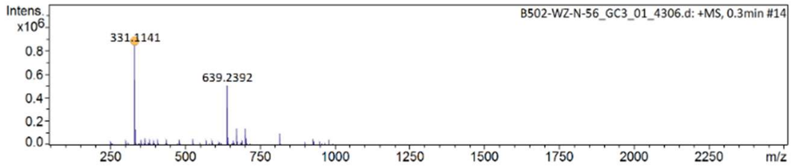

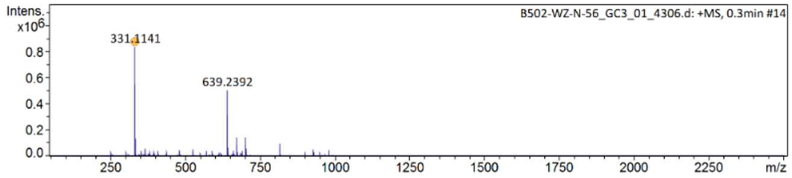

Meas. m/z	#	Ion Formula	m/z	err [ppm]	mSigma	# mSigma	Score	rdb	e⁻ Conf	N-Rule	Adduct
331.1141	1	C16H20NaO6	331.1152	3.4	4.0	1	100.00	7.0	even	ok	M+Na

Figure S97. UV spectrum of compound **8**

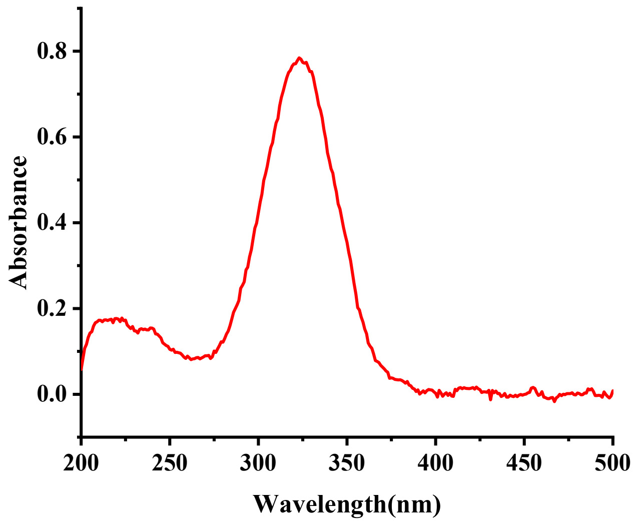

Figure S98. IR spectrum of compound **8**

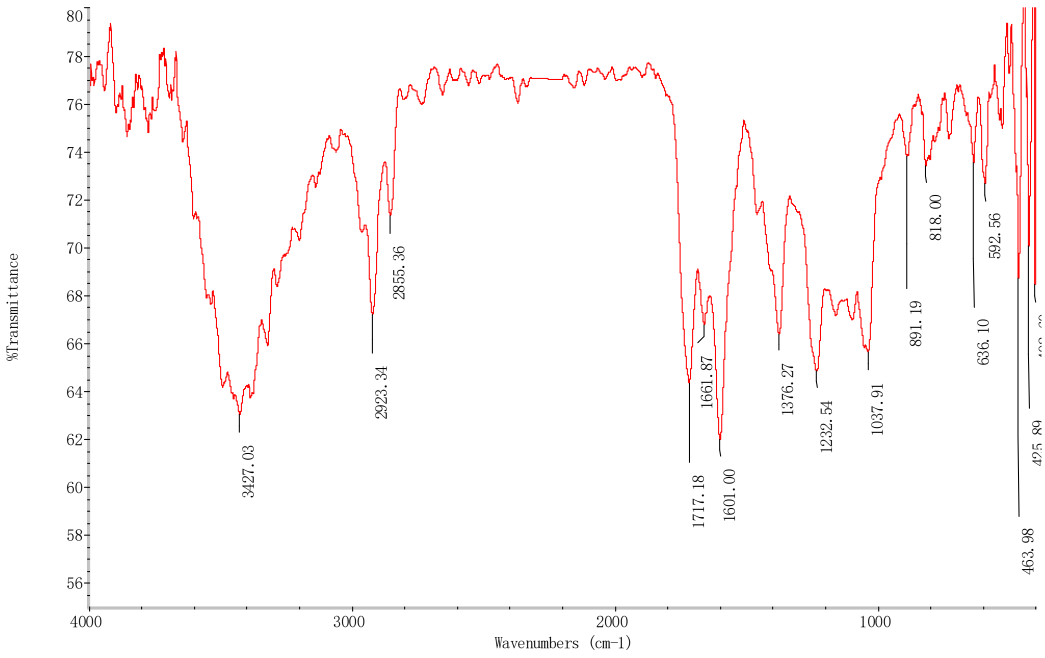

Figure S99. ^{1}H NMR (600 MHz, $CDCl_3$) spectrum of compound **9**

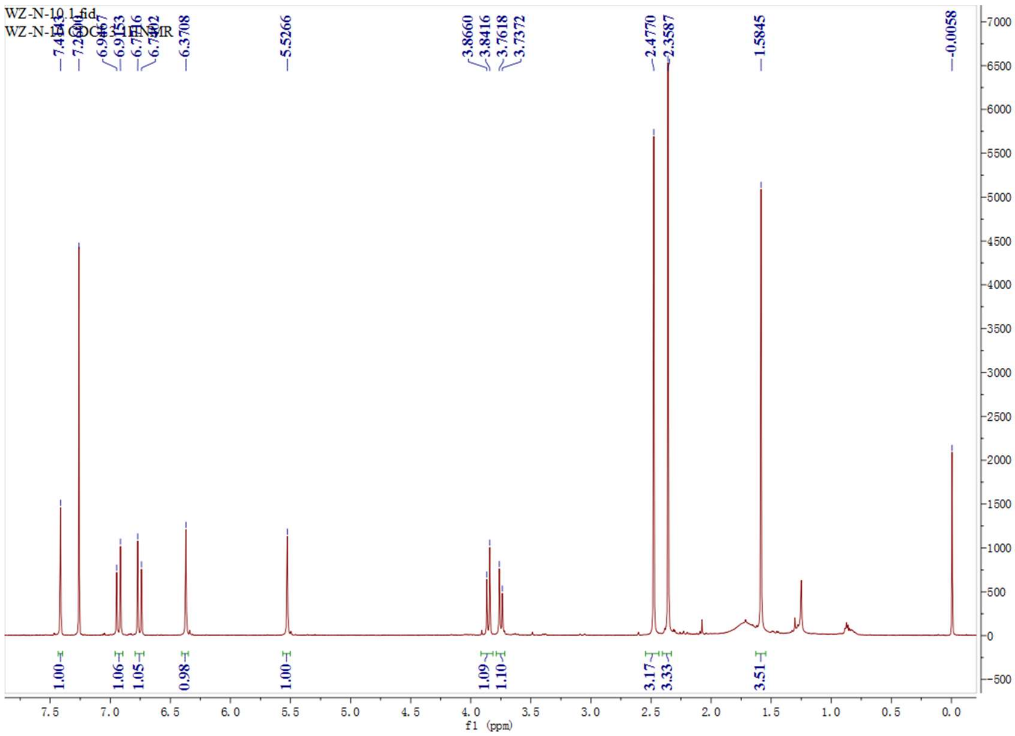

Figure S100. ^{13}C NMR (125 MHz, $CDCl_3$) spectrum of compound **9**

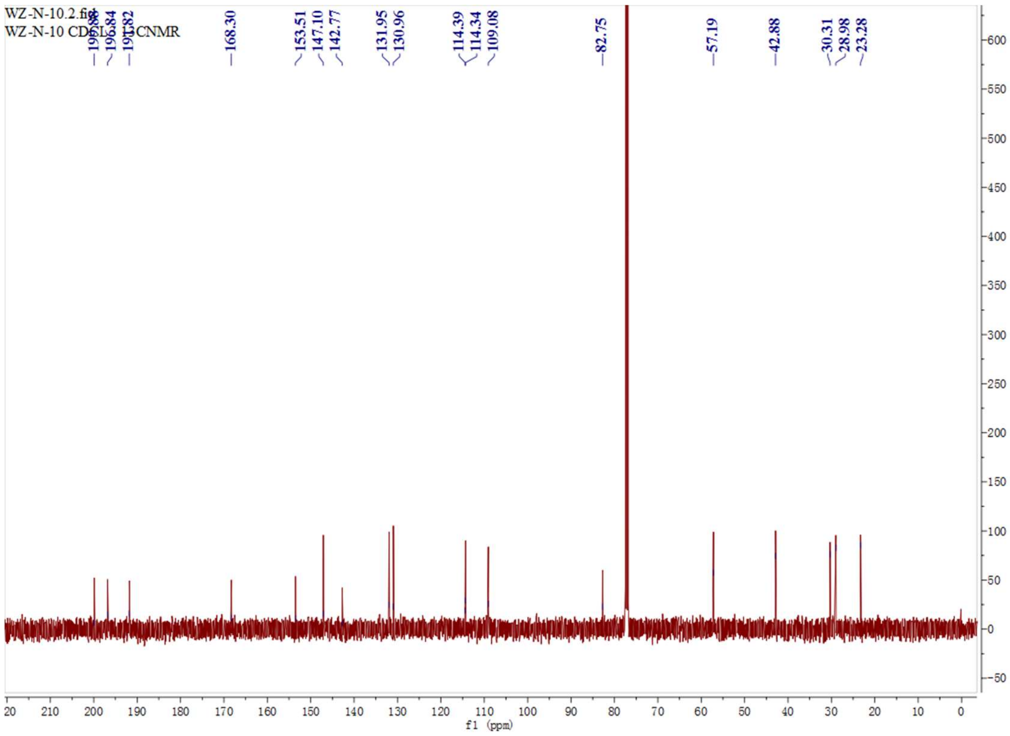

Figure S101. DEPT spectrum of compound **9**

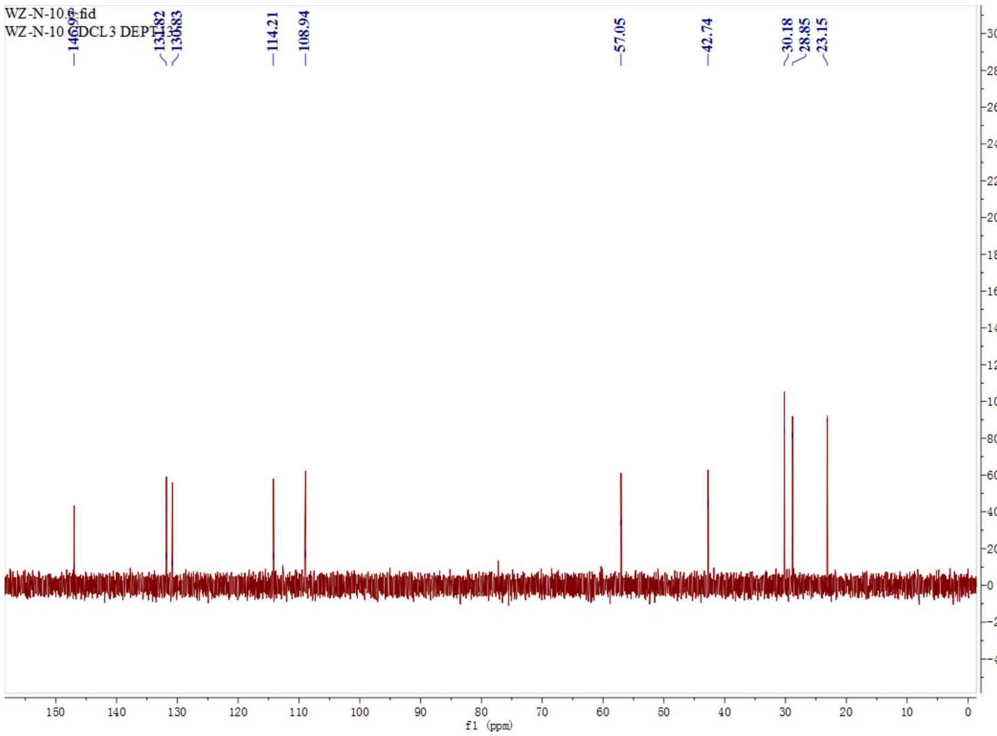

Figure S102. HSQC spectrum of compound **9**

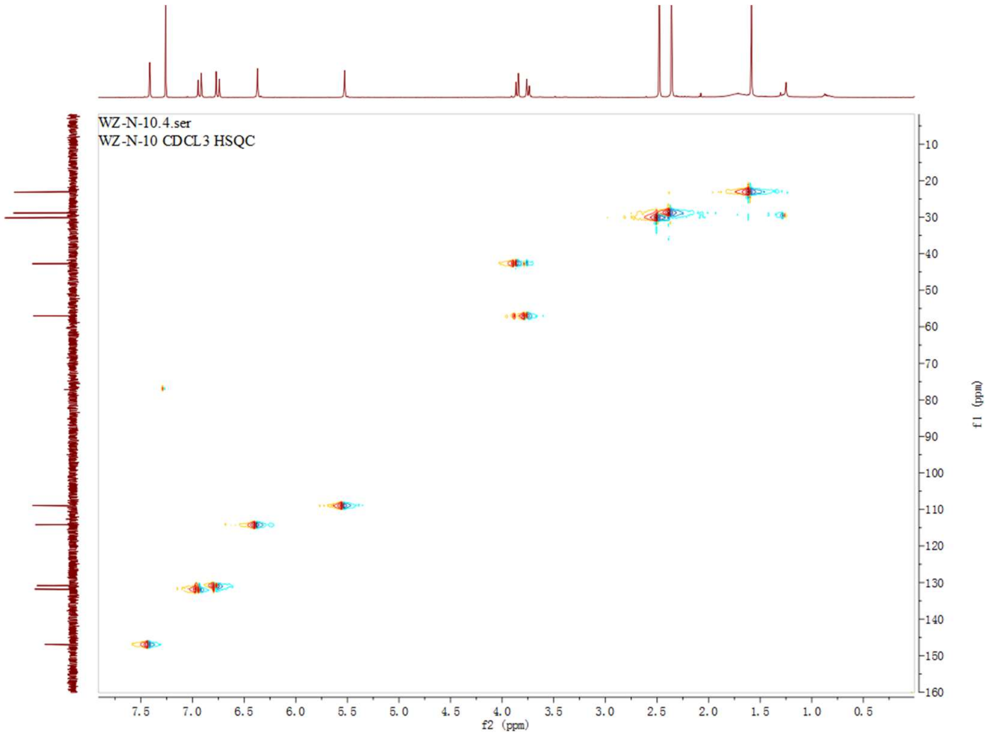

Figure S103. HMBC spectrum of compound **9**

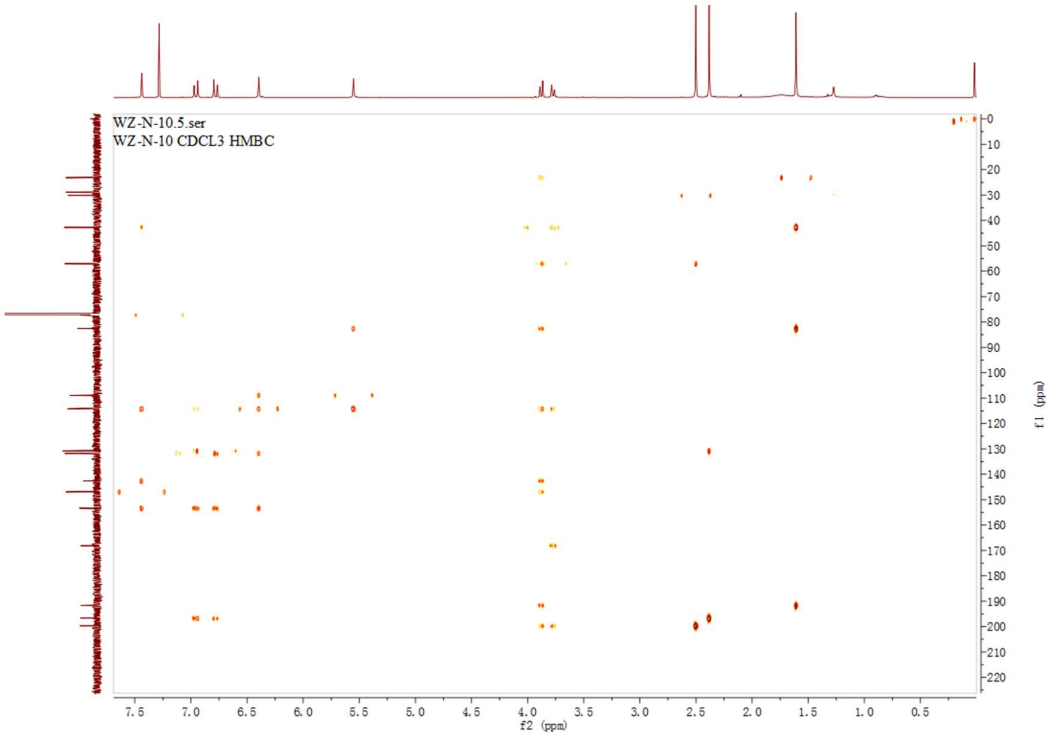

Figure S104. ^{1}H-^{1}H COSY spectrum of compound **9**

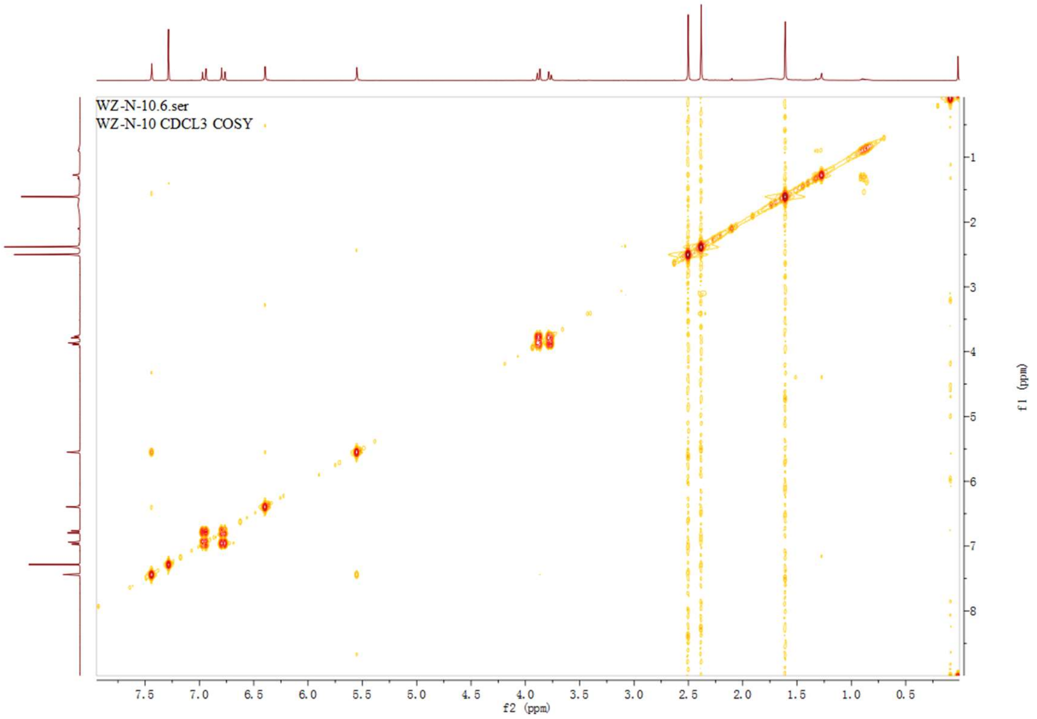

Figure S105. NOESY spectrum of compound **9**

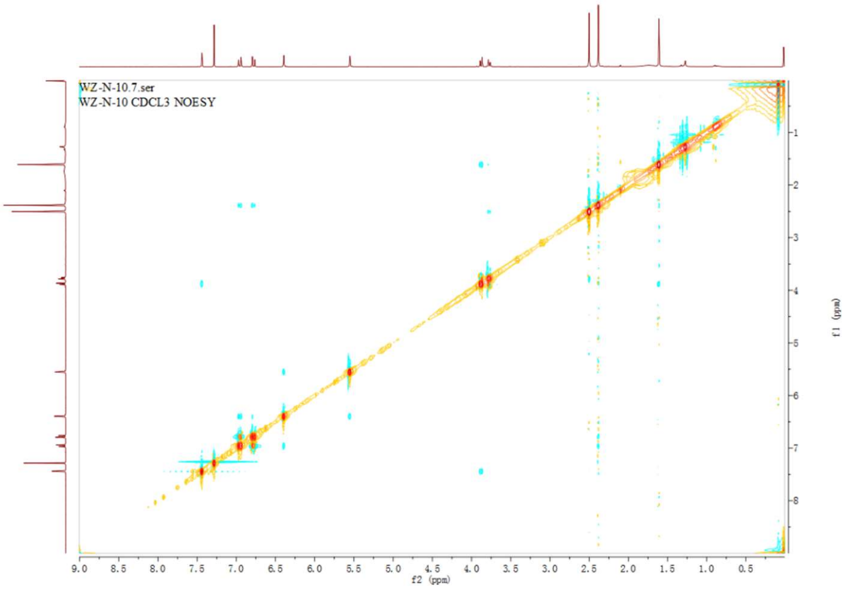

Figure S106. HRESIMS spectrum of compound **9**

Mass Spectrum SmartFormula Report

Analysis Info

		Acquisition Date	2022-05-17 10:23:47
Analysis Name	D:\Data\A501\WYL\2022\20220517\B502-WZ-N-10_GB8_01_4303.d		
Method	lc-ms_as_ms-0.5MIN.m	Operator	Demo User
Sample Name	B502-WZ-N-10	Instrument	compact 8255754.20156
Comment			

Acquisition Parameter

Source Type	ESI	Ion Polarity	Positive	Set Nebulizer	1.8 Bar
Focus	Not active	Set Capillary	3500 V	Set Dry Heater	220 °C
Scan Begin	50 m/z	Set End Plate Offset	-500 V	Set Dry Gas	4.0 l/min
Scan End	2500 m/z	Set Charging Voltage	2000 V	Set Divert Valve	Waste
		Set Corona	0 nA	Set APCI Heater	0 °C

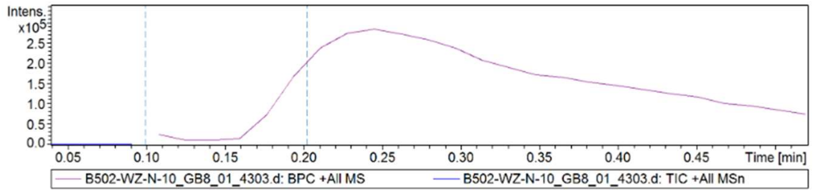

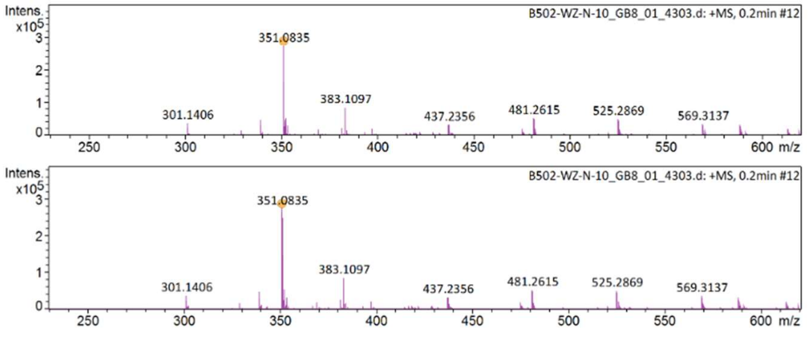

Meas. m/z	#	Ion Formula	m/z	err [ppm]	mSigma	# mSigma	Score	rdb	e⁻ Conf	N-Rule	Adduct
351.0835	1	C18H16NaO6	351.0839	1.0	1.0	1	100.00	11.0	even	ok	M+Na

Figure S107. UV spectrum of compound **9**

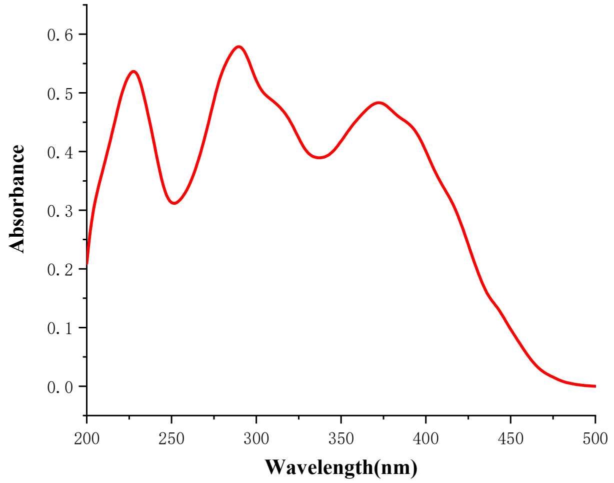

Figure S108. IR spectrum of compound **9**

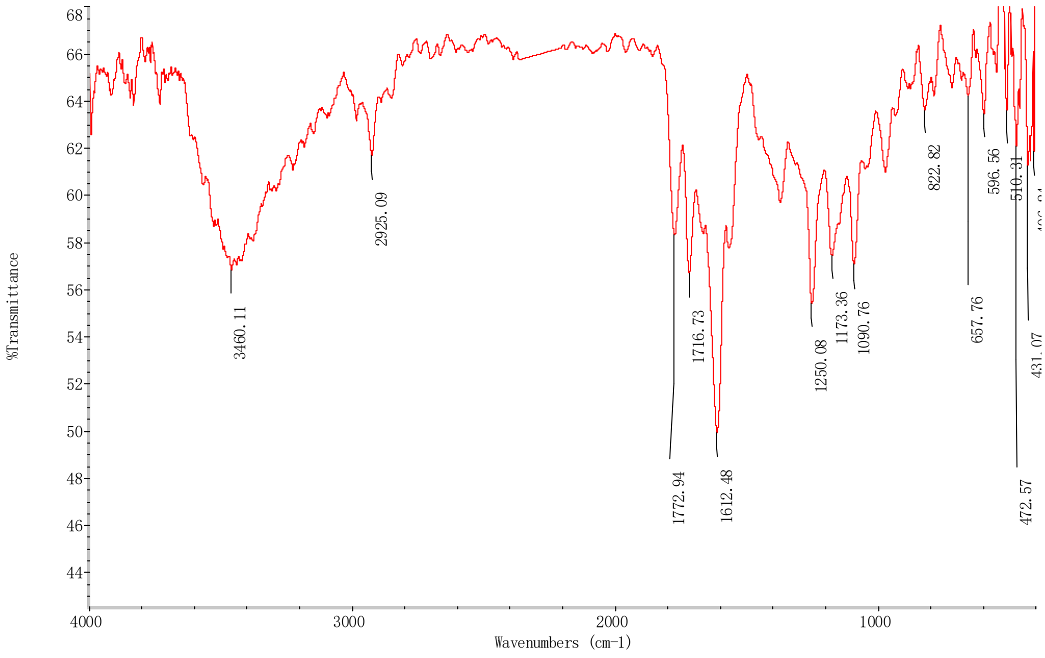

Supplement: Supplementary file 1 [file marinedrugs-21-00075-s001.zip › marinedrugs-2088913-supplementary.pdf]
